# Dynamic restrengthening and fault heterogeneity explain megathrust earthquake complexity

**DOI:** 10.1038/s41467-026-71722-3

**Published:** 2026-04-27

**Authors:** Jeremy Wing Ching Wong, Alice-Agnes Gabriel, Wenyuan Fan

**Affiliations:** 1 Institute of Geophysics and Planetary Physics, Scripps Institution of Oceanography University of California, San Diego, CA USA; 2 Department of Earth and Environmental Sciences, Ludwig-Maximilians-Universität München, Munich, Germany

**Keywords:** Seismology, Natural hazards

## Abstract

Megathrusts host Earth’s largest earthquakes. Understanding the physical conditions controlling their rupture dynamics is critical for assessing seismic and tsunami hazards. These earthquakes often display complex rupture dynamics, exemplified by the 2011 Tohoku-Oki earthquake, which exhibited multiple rupture episodes, depth-dependent seismic radiation, and substantial tsunamigenic slip near the trench. However, how such complexity arises from preexisting physical conditions remains uncertain. Here, we demonstrate that the observed rupture complexity of the Tohoku-Oki earthquake can spontaneously and self-consistently emerge, driven by rapid coseismic frictional restrengthening and data-informed fault heterogeneity. We use an ensemble of 3D dynamic rupture simulations to identify that mixed downdip pulse-like and updip crack-like rupture are driven by dynamic stress redistribution with episodic rupture reactivation. By featuring low fault strength compared to its dynamic stress drop, a preferred model can consistently reproduce the observed complex depth-dependent propagation speeds, multiple rupture fronts as imaged by back-projection, and large tsunamigenic slip at the trench. Our findings demonstrate that preexisting fault heterogeneity conjointly with dynamic frictional weakening and restrengthening drives seemingly unexpected megathrust rupture complexity, highlighting the need to include dynamic effects into physics-based seismic and tsunami hazard assessments of future earthquakes.

## Abstract (web summary)

Using 3D dynamic rupture simulations, this study shows that rapid coseismic frictional restrengthening and fault heterogeneity explain the 2011 M9 Tohoku-Oki earthquake’s rupture reactivation, depth-dependent seismic radiation, and large near-trench slip.

## Introduction

Large megathrust earthquakes propagate rapidly, rupture over hundreds of kilometers within minutes, generate strong ground shaking, and, in certain instances, cause devastating tsunamis. The resulting seismic and tsunami hazards are directly controlled by rupture dynamics and shallow fault slip behavior (e.g., ref. ^1^). However, the physical mechanisms controlling devastating earthquake rupture dynamics remain poorly understood. The 2011 *M*_*W*_9.0 Tohoku-Oki, Japan, earthquake, one of the most destructive earthquakes of the 21st century, exhibited unexpected complexities throughout its rupture process: possible reactivation at the hypocenter^2–4^, depth-dependent seismic radiation^5,6^, large slip to the trench exceeding 50–60 m^7,8^, and an unusually limited along-strike rupture extent for its magnitude^9^. Although the Tohoku-Oki earthquake is among the best-recorded megathrust events, the physical mechanisms underlying its complexity remain debated^9^, and its slip models show significant variability^10^. Previous studies attribute some of this event’s complexities to preexisting stress or frictional-strength fault asperities^11–17^. Here, we use 3D dynamic rupture simulations to show that the surprising characteristics of the Tohoku-Oki earthquake arise dynamically during rupture evolution. We analyze the interplay between preexisting fault heterogeneity and dynamically evolving rupture processes as drivers of earthquake complexity, as well as their distinct observational signatures.

The frictional properties of fault rocks and gouges govern fault strength and slip, and thus are a fundamental component of dynamic rupture models^18^. A key factor is the frictional response during fault slip, which controls earthquake nucleation, propagation, and arrest^19–21^. Rate-and-state friction laws effectively describe fault friction at interseismic to slow slip rates, capturing the dependence of friction on sliding velocity and state evolution^22,23^. Laboratory and theoretical studies show that at coseismic slip rates, fault friction likely exhibits even stronger velocity-weakening, followed by equally rapid frictional healing as slip rate decreases^24–26^. Such rapid dynamic friction evolution generates complex faulting behavior, facilitating diverse earthquake rupture styles and speeds, slip reactivation, and multi-fault interaction in laboratory experiments and numerical simulations^27–29^. However, currently, no fully dynamic rupture model of the Tohoku-Oki earthquake has accounted for fast-velocity weakening rate-and-state friction, and none in combination with data-constrained fault heterogeneity.

The temporal evolution of frictional strength governs the local stability of slip, whereas the spatial distribution of fault stress and strength is equally important (e.g., ref. ^30^). Fault heterogeneity is expected along the megathrust interface, as evidenced by the broad range of faulting behavior observed along the Japan Trench^31^ and in other subduction zones^32^. Such variability may arise from differences in prestress, fault strength, fluid content, geometry, and material properties^33^. While some fault heterogeneity reflects relatively static factors such as depth, temperature, and lithology, these alone cannot explain the full range of observed slip behavior. In addition, fault heterogeneity can evolve dynamically during slow and fast slip. For example, stress heterogeneity can spontaneously emerge even under spatially uniform frictional conditions^34–36^. Therefore, understanding megathrust rupture dynamics requires studying both pre-existing fault heterogeneity and dynamically evolving stresses and friction.

Here, we present fully dynamic 3D rupture simulations of the Tohoku-Oki earthquake incorporating fast velocity-weakening rate-and-state friction and coseismic restrengthening, constrained by observationally informed fault heterogeneity. Our simulations spontaneously reproduce the event’s reported complex rupture behavior, including repeated rupture reactivation, depth-dependent rupture styles, and realistic trench slip. The physical processes identified here, driven by rapid coseismic frictional restrengthening and fault heterogeneity, are likely fundamental controls on rupture behavior in other megathrust settings, carrying important implications for earthquake dynamics, tsunami generation, and hazard assessment globally.

## Results

3D dynamic rupture simulations can capture the nonlinear interactions between seismic wave propagation, fault friction, stress heterogeneity, and fault geometry, leveraging high-performance computing, reaching megathrust earthquake spatial and temporal scales at high resolution^37^. Dynamic rupture simulations have been applied to subduction zones worldwide (“Methods”). However, many studies have been restricted to 2D cases with imposed ad-hoc fault friction or stress heterogeneities^11,14,16^ or simplified friction laws^38^, restricting the direct integration of observational constraints and verification. Here, we present fully dynamic 3D rupture simulations of the Tohoku-Oki earthquake incorporating fast velocity-weakening and coseismic frictional restrengthening, constrained by observationally informed fault heterogeneity. Unlike previous dynamic rupture models that prescribed frictional or stress asperities to control slip distribution and steer the depth-dependent rupture behavior, our simulations spontaneously reproduce the observed complex rupture behavior, including repeated rupture reactivation, depth-dependent rupture styles, and realistic trench slip, solely through dynamic friction evolution and stress conditions.

**Fig. 1:**
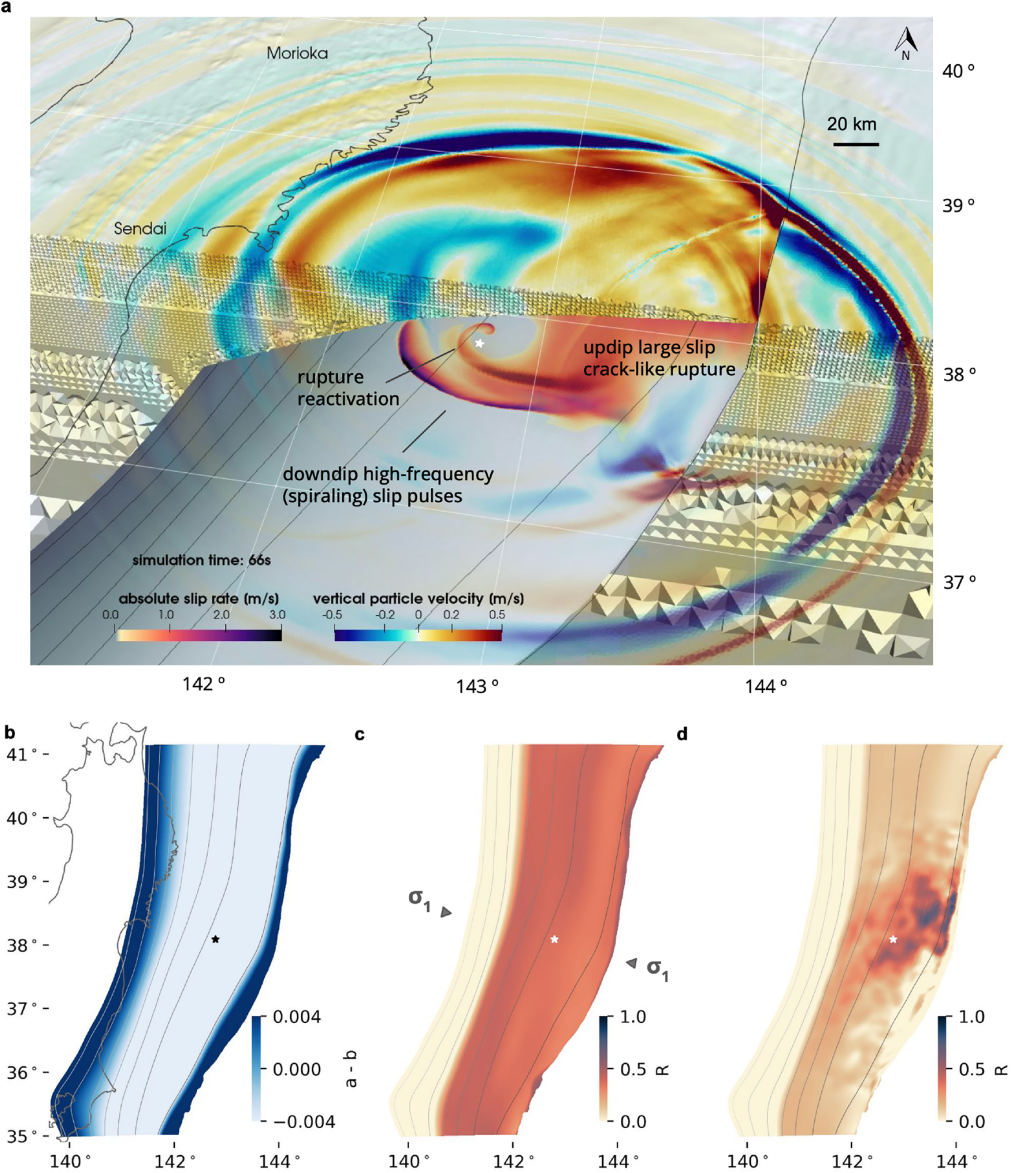
Overview of 3D dynamic rupture simulations of the 2011 Tohoku-Oki earthquake and their initial conditions. Depth contours (gray, 10 km intervals) and hypocenter location (star) are shown in all panels. **a** Snapshot of the simulated absolute slip rate and seismic wavefield evolution (vertical particle velocity) at 66 s, highlighting multiple reactivated slip pulses propagating downdip and crack-like rupture accumulating large slip near the trench. The model incorporates realistic slab geometry and high-resolution topobathymetry within an unstructured tetrahedral mesh refined near the slab and onshore region. **b** Depth-dependent frictional properties (*a* − *b*, see Methods Sec. “Fault friction”) with velocity-strengthening behavior in the shallow ( < 9 km) and deep ( > 45 km) regions, transitioning to velocity-weakening in the seismogenic zone. **c** Laterally homogeneous, depth-dependent ambient stress and frictional strength initial conditions informed by regional tectonics (see Methods Sec. “Prestress”), showing the relative prestress ratio *R* (maximum possible stress drop over frictional strength drop, Eq. (15),^138^). The principal stress direction (*σ*_1_ at an azimuth of 100° and a plunge angle of 8°,^39^) is indicated with arrows. **d** Heterogeneous stress initial conditions combining the ambient background stress shown in (**c**) and heterogeneous initial stress inferred from the median slip distribution of 32 data-constrained slip models (Supplementary Figs. S1,S2^10^). Along-dip initial shear stresses are shown in Supplementary Fig. S3.

Our 3D dynamic rupture models (Fig. 1), resolving up to 1.5 Hz of the seismic wavefield (Methods Sec. “Model geometry and mesh” and Supplementary Sec. “SM: Model domain and resolution”), investigate physical controls on complex, spontaneous rupture processes by incorporating regional-tectonic constraints^39^ (Fig. 1c), and initial stress heterogeneity (Fig. 1d). We construct an initial stress state (Methods Sec. “Prestress”) that combines the regional maximum principal stress orientation with stress heterogeneity from a median slip model that captures common slip features among 32 finite-fault slip models^10^ (Supplementary Fig. S1). The dynamic models use a realistic slab geometry and high-resolution topobathymetry (Fig. 1a), along with fast velocity-weakening rate-and-state friction^24^ (Fig. 1b and Supplementary Table S1) and off-fault plasticity (Supplementary Fig. S11).

Dynamic rupture is governed by a fast velocity-weakening rate-and-state friction law (Methods Sec. “Fault friction”), in which frictional strength is inversely proportional to slip rate at high slip rates. This formulation represents thermal weakening processes that can operate on natural faults at co-seismic slip rates, such as thermal pressurization and flash-heating^40,41^. We use the full rate-and-state friction formulation, including state evolution (Methods Sec. “Fault friction”, Equations 5 and 6). In this formulation, the steady-state friction coefficient is given as

1 $${f}_{ss}={f}_{w}+\frac{{f}_{LV,ss}(V)-{f}_{w}}{{(1+{(V/{V}_{w})}^{4})}^{1/4}}.$$

It depends on slip rate *V* and transitions from a low-velocity steady-state friction coefficient *f*_*LV*,*ss*_(*V*) to a fully weakened level *f*_*w*_ once *V* exceeds the onset of weakening velocity *V*_*w*_. The low-velocity steady-state friction coefficient is defined as

2 $${f}_{LV,ss}={f}_{0}-(b-a)\ln(V/{V}_{0}),$$

where *f*_0_ is the reference friction coefficient, *V*_0_ is the reference velocity, *a* is the direct-effect parameter, and *b* is the state-evolution parameter. At low slip-rates (*V* ≪ *V*_*w*_), friction follows the classical rate-and-state friction law, whereas for high slip rates (*V* ≥ *V*_*w*_), an additional fast velocity-weakening term is activated, producing a rapid reduction in frictional strength.

When the fault local slip rate decelerates below *V*_*w*_, the inverse slip rate dependence of *f*_*ss*_(*V*) recovers towards *f*_*LV*,*ss*_(*V*), producing rapid frictional healing in the tail of slip pulses^41,42^. This fast velocity-weakening law is capable of generating a wide spectrum of rupture styles, including sub- and super-shear, crack-like, and pulse-like ruptures^27^.

In our simulations, the frictional parameters (*a*, *b*, *f*_*w*_, and *V*_*w*_) are fixed across the ensemble (Table S1), following previous studies^27,28^. We prescribe a depth-dependent profile of the rate-and-state friction parameters (*a* − *b)* (Fig. 1b). The shallow megathrust is assigned velocity-strengthening friction (*a* − *b* > 0), consistent with laboratory constraints for clay-rich lithified rock and rock gouges at low slip velocities^43^. The seismogenic zone (10–45 km depth) is assigned velocity-weakening friction (*a* − *b* < 0). At the downdip limit of the seismogenic zone ( > 45 km), we revert to velocity-strengthening friction, consistent with inferences from repeating earthquakes and slow-slip events^9^. For more details, see the Methods Sec. “Fault friction.” Aside from the depth-dependent variation in (*a* − *b)*, we do not prescribe any additional frictional asperities. All other frictional parameters remain spatially uniform and are held constant across the model ensembles (Table S1).

### Grid search for preferred rupture model

We use a systematic grid-search approach to identify a preferred 3D dynamic rupture model that minimizes the misfit with respect to the onshore and offshore geodetic observations and SCARDEC seismic moment-rate^44^, with each dataset weighted equally. The preferred model is selected from an ensemble of simulations that systematically vary (i) the amplitude of initial stress heterogeneity informed by finite-fault slip models (*α*) and (ii) the regionally constrained ambient stress, expressed as the ratio of maximum possible stress drop over fault strength on an optimally oriented fault plane (*R*_0_). Larger *α* represents higher prestress heterogeneity informed by finite-fault slip models, whereas larger *R*_0_ reflects an ambient stress state closer to failure. Figures 1 and S3 show the resulting initial shear stress, relative prestress ratio *R* across the megathrust. The along-strike and downdip variation of *R* and initial shear stress *τ*_0_ along the megathrust reflects the resolved initial shear traction on the 3D fault geometry.

Together, *α* and *R*_0_ govern the earthquake energy balance between the available energy release rate that sustains rupture propagation and the fracture energy required for continued rupture growth^30^. We vary *α* from 1.0 to 1.2 in increments of 0.01 and *R*_0_ from 0.1 to 0.2 in increments of 0.005, generating 33 dynamic parameter sets. Each corresponding spontaneous dynamic rupture scenario is quantitatively evaluated by the variance reduction of observed onshore and offshore static displacements and seismic moment release rate (Supplementary Figs. S12 and  S13). The ensemble results suggest that *α* primarily controls peak slip amplitude, whereas *R*_0_ controls the overall rupture area. Variations in crack- or pulse-like rupture style and the occurrence of healing fronts arise from the nonlinear interaction with the initial stress conditions parameterized by *α* and *R*_0_, which control the amplitude and heterogeneity of the prestress but do not modify the frictional healing law itself.

**Fig. 2:**
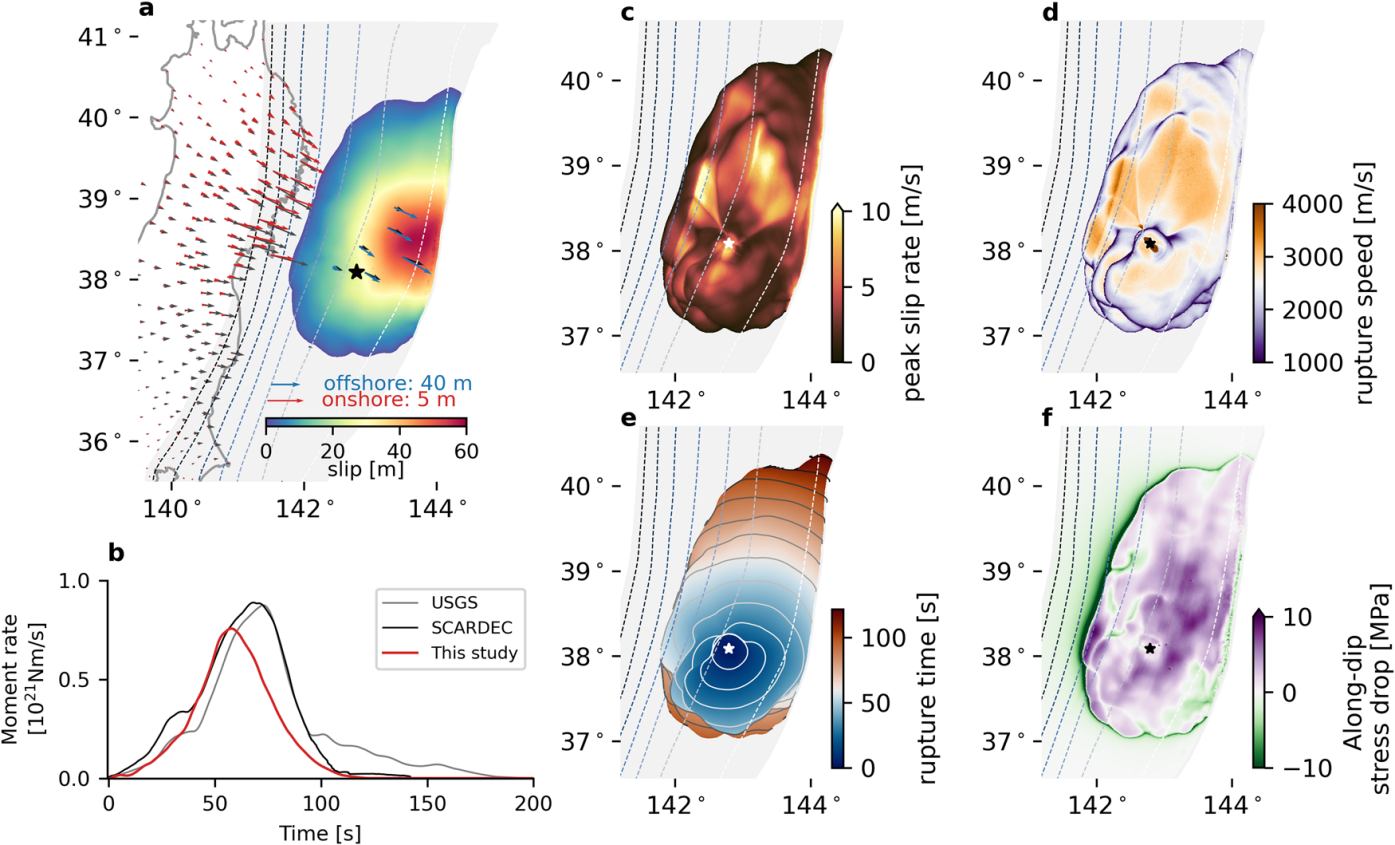
Preferred 3D dynamic rupture scenario of the Tohoku-Oki earthquake constrained by geodetic observations and seismic moment release rate. Gray contours indicate depth (10 km intervals), and the star is the hypocenter location^163^. Rupture extends 200 km along-dip and 360 km along-strike, producing a moment magnitude of *M*_*W *_8.97 and a duration of 120 s. The total radiated seismic energy is  ≈ 7.7 × 10^17^*J*, within observational estimates of 4.2 − 9.1 × 10^17^*J* for the Tohoku-Oki earthquake^3,6^. **a** Fault slip distribution with comparison between observed and simulated geodetic displacements onshore and offshore. Black arrows denote observed horizontal displacements from offshore and onshore stations. Blue and red arrows represent simulated horizontal displacements offshore and onshore, respectively, achieving variance reductions of 77% (onshore) and 55% (offshore). **b** Synthetic moment rate release compared with observational inferences from teleseismic by the USGS model^163^ and SCARDEC inversion results^44^. Heterogeneous spatial distributions of **c** peak slip rate, **d** rupture speed, **e** rupture front timing (10 s intervals, gray contours), and **f** along-dip stress drop.

The preferred model reproduces the overall slip pattern of the Tohoku-Oki earthquake. Despite not being a full-scale inversion, the model achieves variance reductions of 77% and 55% for onshore and offshore geodetic observations, respectively (Fig. 2a). It features a smooth slip distribution, with major slip concentrated updip of the hypocenter, and a triangular moment-rate function consistent with observations (Fig. 2b). We capture the gradual seismic moment release during the earthquake’s initiation phase, in contrast to previous dynamic models (e.g., refs. ^13,16^, Supplementary Sec. “SM2: Nucleation”). Despite the simple slip distribution and moment rate release and the absence of imposed frictional heterogeneity, the model produces substantial rupture complexity, including variations in peak slip rate, rupture speed, and stress drop (Fig. 2c–f), as reported in kinematic slip models (e.g., refs. ^4,45^). The model yields an average stress drop of 2.4 MPa, matching the average reported stress drop estimates^46^ (Supplementary Sec. “SM3: Dynamic stress drop”). The coseismic stress drop distribution from our model aligns with the major afterslip pattern of the Tohoku-Oki earthquake between 40–60 km depth^9^, where negative stress drop is prominent at the downdip edge of the simulated rupture area. In the following, we examine four key dynamic rupture characteristics of the preferred model and their corresponding observational signatures.

### Frictional restrengthening drives rupture reactivation

**Fig. 3:**
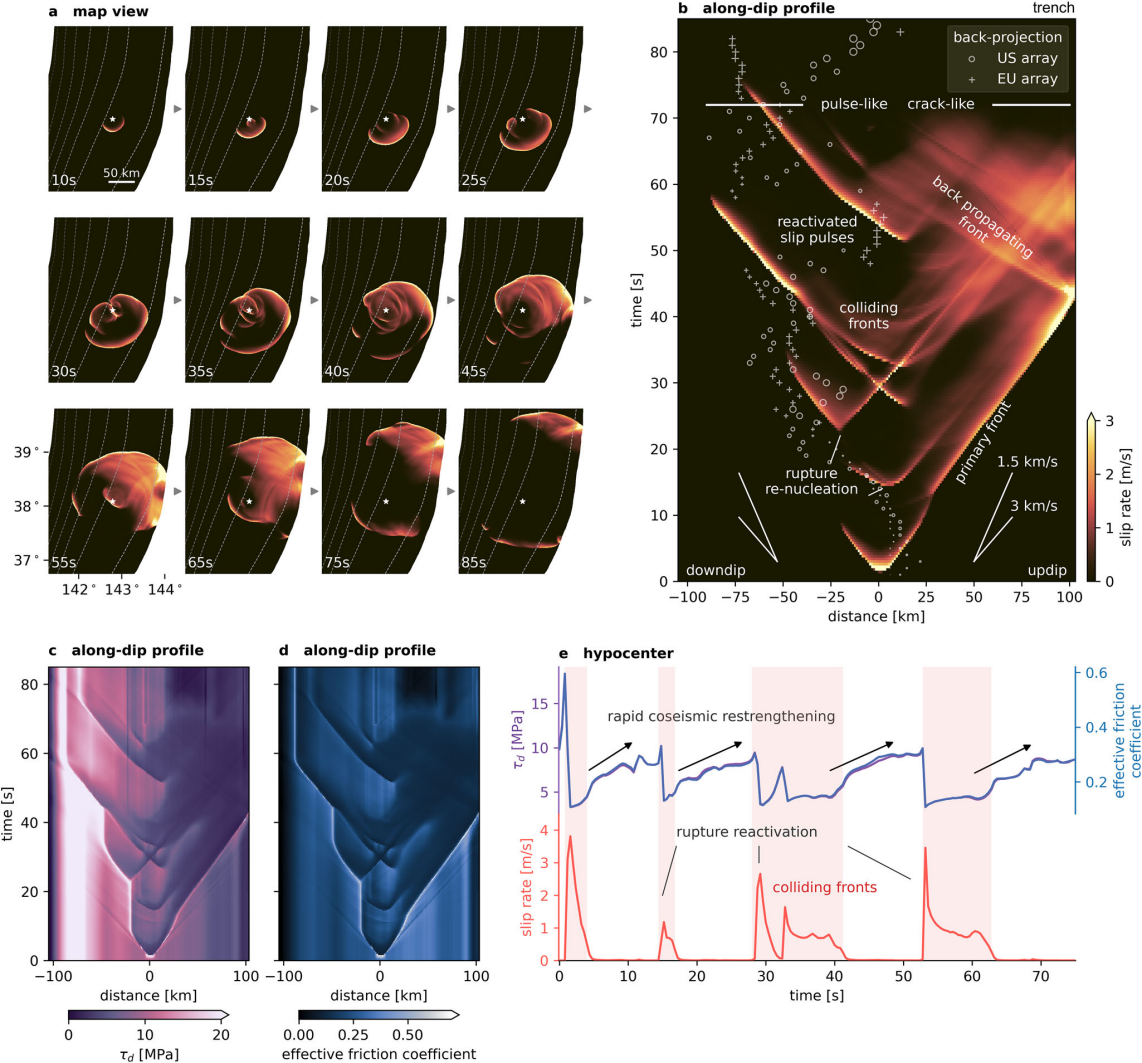
Repeated dynamic rupture reactivation enabled by rapid coseismic weakening and restrengthening during the preferred Tohoku-Oki earthquake dynamic rupture model. **a** Map-view snapshots of rupture evolution from 10 s to 85 s simulation time, showing three main re-nucleation episodes at 15 s, between 25 and 40 s, and at 50 s. The white star indicates the hypocenter. Similarly, “spiraling” rupture fronts have been observed in recent laboratory experiments^49^. Supplementary Fig. S4 and Supplementary Video S1 show the complete rupture evolution. Supplementary Fig. 5 shows the detailed evolution of “spiraling” rupture fronts. **b** Slip rate evolution along a dip profile through the hypocenter, highlighting multiple episodes of rupture reactivation. Crosses and circles indicate the locations of high-frequency radiation from back-projection using the US and European arrays, respectively^5^. Rupture propagates faster updip ( ≈ 2.5 km/s) compared to downdip ( ≈ 1.7 km/s), matching the observational results from the back-projection analyses^5^. **c**, **d** Temporal evolution of along-dip shear stress (purple) and effective friction coefficient (blue, Methods “Fault friction”) along the hypocentral dip profile, highlighting rapid variations coincident with dynamic rupture reactivation; lighter colors indicate higher values. **e** Time series at the hypocenter of slip rate (red), along-dip shear stress (purple), and effective friction coefficient (blue), showing repeated rupture reactivation (slip rate ≥ 0.05 m/s, shaded red) and rapid frictional restrengthening. Supplementary Fig. S5 shows updip and downdip time series evolution of slip rate, along-dip shear stress, and effective friction coefficient.

Our preferred dynamic rupture model spontaneously produces episodic pulse- and crack-like slip reactivation originating near the hypocenter (Fig. 3, Supplementary Fig. S4, and Supplementary Video S1). Crack-like rupture involves prolonged local slip durations (rise time) comparable to the total event duration. In contrast, self-healing pulse-like rupture is characterized by short rise times and a “healing" front following rupture front driven by rapid coseismic fault strength recovery^42,47,48^. Our preferred model has complex rupture dynamics, including multiple spiraling^49^, back-propagating^27,50^, and colliding rupture fronts driven by rapid coseismic frictional weakening and restrengthening. Figure 3a shows the complexity of slip rate evolution through multiple rupture reactivation episodes. The rupture initiates as a primary slip pulse with short local slip duration, followed by a secondary slip pulse nucleating at its healing front. Interaction between updip and downdip propagating rupture and healing fronts leads to a successive second, third, and (unsustained) fourth episodes of slip reactivation in the hypocentral region. When the updip propagating rupture fronts reach the seafloor, strong dynamic interactions with the free surface generate trench-reflected, back-propagating phases, which coalesce with secondary arriving rupture fronts to form a sustained updip crack-like rupture with prolonged slip duration. In the later stage, rupture simplifies into a bilateral slip pulse propagating along-strike, saturating the seismogenic zone width before spontaneously arresting (Supplementary Fig. S4 and Video S1).

Our model explains the observed contrast between the slow downdip rupture propagation speed^5^ and the faster updip speed^2,4,45^. Figure 3b shows how episodic pulse-like rupture reactivation successively extends the rupture duration downdip, producing a slower apparent rupture speed ( ~ 1.5 km/s) despite each reactivated front propagating at a regular rupture speed ( ~ 2.5 km/s). In contrast, the updip rupture front propagates steadily as a primary crack-like rupture front at  ~ 2.5 km/s.

Rapid coseismic restrengthening emerges as the principal mechanism controlling downdip repeated reactivation, causing a fault portion to slip and stop more than once during the same earthquake. The mechanisms driving rupture reactivation are illustrated by the along-dip evolution of shear stress and frictional strength (Fig. 3c, d), and by the corresponding time series at the hypocenter (Fig. 3e).

Initially, as slip rate increases, both along-dip shear stress and effective friction coefficient sharply rise due to the instantaneous response of rate-and-state friction to slip rate changes, quickly followed by dynamic weakening (see “Methods”), causing dynamic stress drops of up to 10 MPa (Fig. 3e). As slip rate subsequently ceases, a healing front follows. The growing slip pulse gradually concentrates shear stress in its hypocentral region, eventually overcoming local fault strength and reactivating slip, consistent with theoretical predictions for singular, self-similar pulse-like rupture^42^ and simpler 2D numerical simulations^27^. This process repeats multiple times, resulting in six distinct slip episodes at the hypocenter in our preferred rupture model. Notably, all secondary ruptures are pulse-like in the downdip direction and coalesce with the free-surface reflected front into crack-like ruptures in the updip region. The sustained crack-like slip updip and continued dynamic stressing limit the formation of healing fronts, and pulse-like rupture does not develop there. Thus, a key difference is not whether coseismic restrengthening occurs, but whether it is sufficient to arrest dynamic slip. Downdip, restrengthening more readily arrests slip, allowing self-healing pulses and repeated reactivation.

### Downdip slip pulses and updip crack-like rupture

**Fig. 4:**
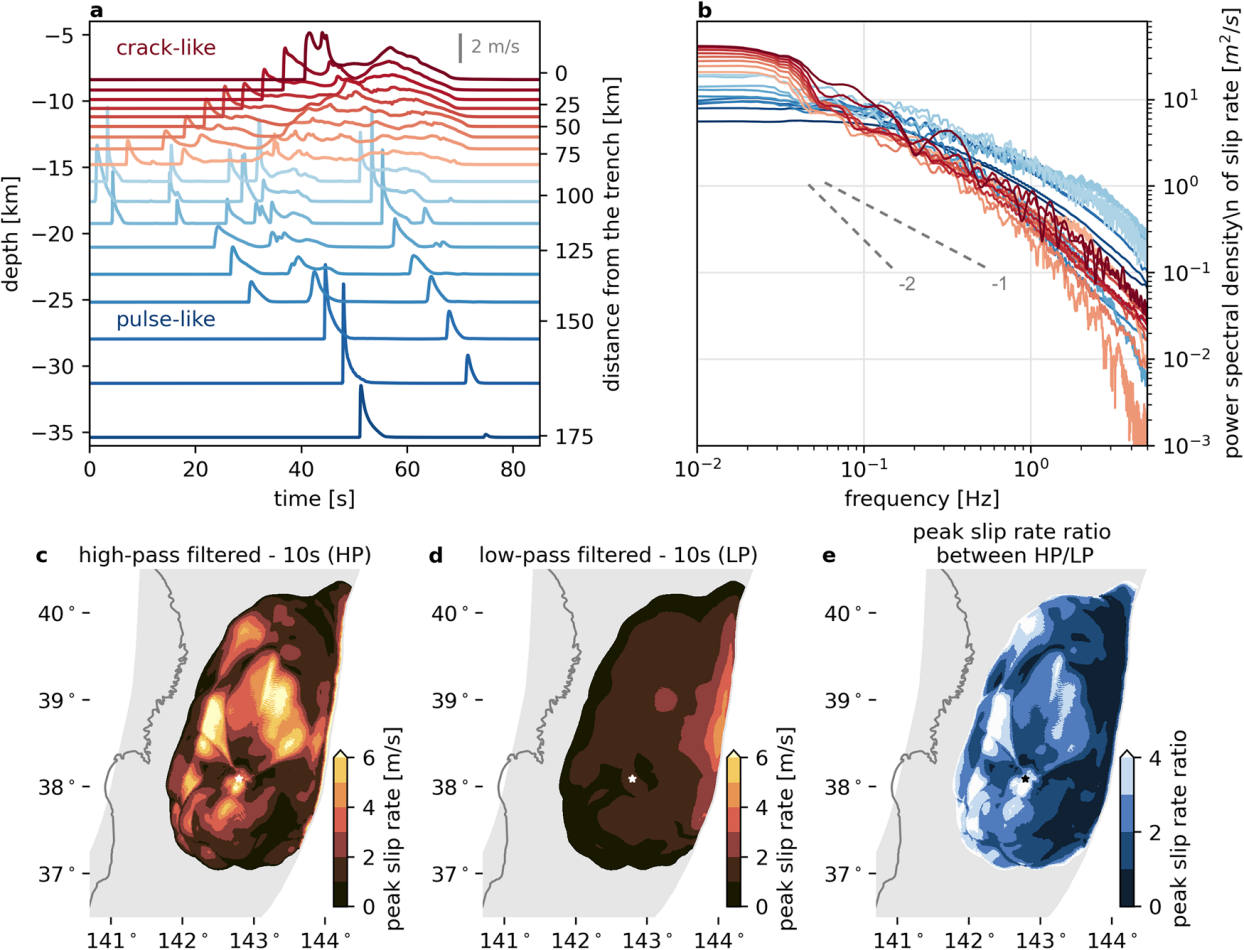
Depth-varying rupture styles featuring downdip short-duration slip pulses and updip large-slip crack-like ruptures. **a** Slip-rate evolution with depth along the same hypocenteral dip profile in Fig. 3, highlighting crack-like ruptures shallower than 15 km depth (red) and pulse-like ruptures at greater depths (blue). **b** Power spectra of the slip rates, illustrating systematically higher-frequency content in downdip pulses compared to the shallower crack-like ruptures. The shallow crack-like rupture spectra follow a -1 slope over the 0.02–1 Hz range, whereas the pulse-like rupture exhibits a shallower -0.7 slope within the same frequency band. **c**, **d** Spatial distribution of high-pass filtered (HP) and low-pass filtered (LP) peak slip rates, respectively. **e** Spatial variation in the ratio of high-frequency to low-frequency peak slip rates (HP/LP). Shallow regions ( < 15 km depth) exhibit predominantly crack-like rupture with low HP/LP ratios, while downdip and hypocentral areas show pulse-like ruptures enriched in high-frequency content, consistent with observations from back-projection and regional strong-ground motion analyses^5,51^. Supplementary Fig. S6 shows additional comparisons in three frequency ranges: 10–2 s, 2–0.5 s, and  > 0.5 s.

Observational studies, including teleseismic back-projection^5^, regional strong-ground motion analyses^51^, and finite-fault inversions^4,45^, have consistently documented depth-dependent seismic radiation characteristics in the Tohoku-Oki earthquake. In our preferred model, we observe depth-varying dynamic rupture styles, characterized by short-duration slip pulses^47^ radiating high-frequency seismic waves downdip and prolonged crack-like rupture accumulating large slip updip (Fig. 4). Short slip pulses at depth are associated with relatively small total slip but strong high-frequency radiation, whereas the shallow crack-like rupture produces large slip with relatively reduced high-frequency content. Thus, along the fault, high-frequency radiation is inversely correlated with local total slip. Figure 4a highlights contrasting slip-rate functions across different depths. At depths shallower than 15 km, rupture propagation is crack-like, characterized by continuous slip and prolonged rise times exceeding 50 s. The prolonged rupture is sustained by successive reactivation of slip triggered by free-surface reflections that generate back-propagating rupture fronts^52,53^. In contrast, rupture transitions to multiple reactivated sharp slip pulses as it propagates deeper than 15 km, each with rise times less than 10 s. These depth-dependent rupture styles become particularly evident during 30–80 s rupture time, when multiple reactivated rupture fronts are present, and the deeper ruptures manifest discrete short-duration pulses (Fig. 4a and Supplementary Fig. S5). Additional profiles from north to south show consistent behavior, with rupture durations systematically increasing towards shallower depth (Supplementary Fig. S7). This rupture style variability influences the associated seismic radiation. Figure 4b shows slip-rate amplitude spectra, revealing that shallow crack-like rupture episodes are relatively depleted in high-frequency energy than the deeper pulse-like rupture portions. However, northern profiles exhibit less contrast in seismic radiation due to a strong peak at the rupture front across all depths (Supplementary Fig. S7). This behavior reflects an intrinsic limitation of our model setup, which does not prescribe any depth-dependent frictional variations that control rupture-tip behavior^14^.

**Fig. 5:**
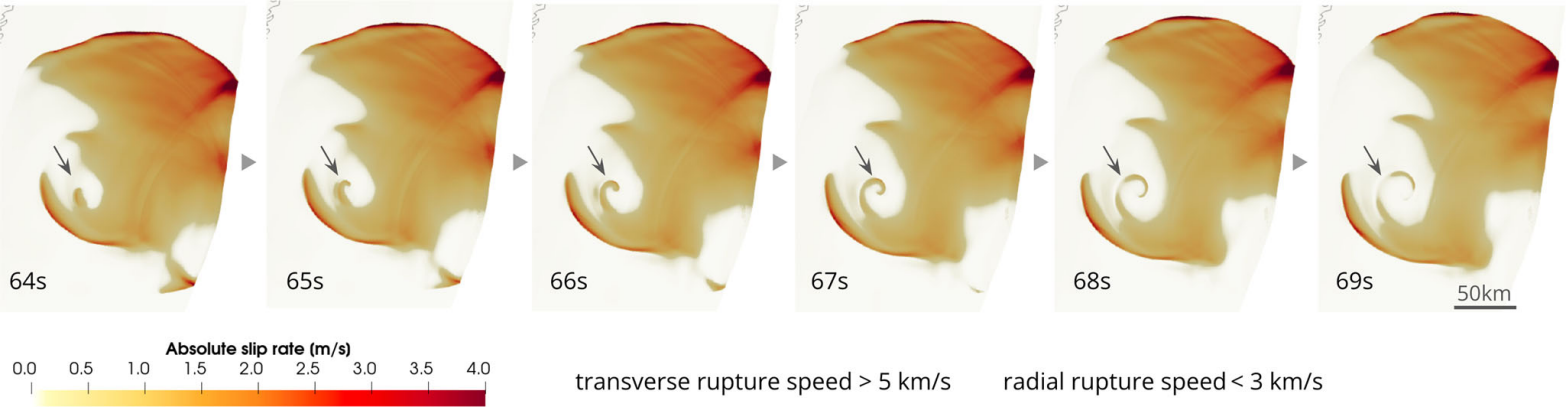
Rapid transverse expansion of a circular rupture front, resembling recent laboratory observations^49^. Snapshots show the slip rate evolution of a circular, spiraling rupture front from 64 s to 69 s. The black arrows mark the onset location where the circular rupture front forms. The spiraling rupture reaches a radius of approximately 12 km from the hypocenter and sweeps a 180° arc within 5 s. Its transverse rupture front expansion speed exceeds 7 km/s, which is higher than the local shear wave speed at the corresponding depth of  ≈ 20 km (Supplementary Table S2).

Observational studies indicate that high-frequency radiation inversely correlates with the total slip (e.g., refs. ^4,6^). Our preferred dynamic rupture model reproduces these observations, showing the amplitude ratio of high-pass to low-pass filtered peak slip-rate function at 10 s period reaching approximately 400% in the downdip and hypocentral regions (Fig. 4e). Supplementary Fig. S6 further compares peak-slip rate distribution across multiple frequency bands and shows that the high-frequency radiations are dominated within the 0.5–2 Hz frequency range used in back-projection studies^5^. Furthermore, the multiple reactivated downdip slip pulses can explain migrating downdip and hypocentral high-frequency seismic radiation imaged through back-projection methods (Fig. 3b)^5^. Downdip reactivated slip pulses also exhibit spiraling rupture. These spiraling rupture fronts are localized and expand rapidly in the transverse direction with rupture speed higher than shear wave speed ( > 7 km/s), while the radial rupture front expansion speed remains sub-shear ( < 3 km/s) (Fig. 5). Such rapid expansion of the rupture area can further enhance the short-period radiation burst at depths^51^. In contrast, shallow regions are dominated by large slip occurring primarily at low frequencies (below 10 s), consistent with crack-like behavior suggested in finite-fault models (e.g., refs. ^4,45^, Fig. 4c–e).

### Large slip to the trench

**Fig. 6:**
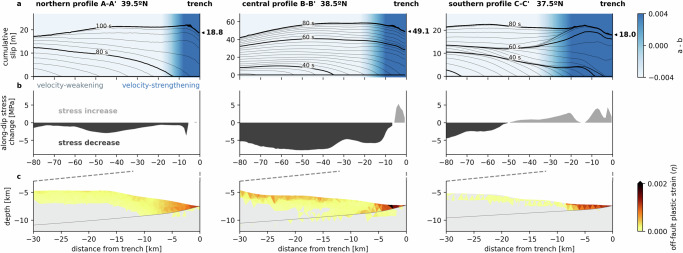
Large near-trench slip occurs despite shallow velocity-strengthening frictional behavior, shallow off-fault plastic deformation, and negative stress drop. Along-dip profiles of slip, stress drop, and off-fault deformation at northern (39.5°, first column), central (38.5°, second column), and southern (37.5°, third column) cross-sections. **a** Evolution of cumulative slip at 2 s (thin lines) and 20 s intervals (thick lines). Background color shading indicates frictional behavior, transitioning from velocity-weakening (light blue) at depth to velocity-strengthening (dark blue) near the trench. Bold values at the right indicate the fault slip amplitude at the trench. **b** Along-dip shear stress change (Δ*τ*), with dark gray representing stress decrease and light gray representing stress increase. **c** Close-up view of the distribution of off-fault plastic strain (color shading, quantified as *η*, Methods Sec. “Off-fault plasticity”) and the megathrust interface geometry (gray line).

Shallow slip reaching the trench is critical for assessing tsunami hazards associated with large megathrust earthquakes. Our simulation demonstrates substantial near-trench slip, driven by coseismic frictional weakening, despite competing effects from shallow off-fault plastic deformation, velocity-strengthening frictional behavior, and the associated negative stress drop. Fig. 6a presents three along-dip profiles of slip from south to north. The modeled trench slip magnitudes of 18.8 m, 49.1 m, and 18.0 m at the southern, central, and northern cross-sections are comparable to differential bathymetry measurements, which indicate horizontal displacements of approximately 50–70 m at the central region and up to 20 m to the northern and southern extents (Supplementary Fig. S8, refs. ^8,54^).

In our model, dynamic frictional weakening effectively sustains shallow rupture propagation, enabling up to 50 m of slip near the trench. Wave-mediated dynamic stressing, including both free-surface-reflected rupture fronts and updip-propagating fronts reactivated downdip, increases shear traction and slip rate on the shallow interface. As shallow slip rates approach and eventually exceed *V*_*w*_, fast-velocity weakening friction is activated, producing a second acceleration phase (Fig. 3b and Supplementary Fig. S5a). The velocity-strengthening friction (≤9 km depth) and fast velocity-weakening friction adopted in our model are motivated by laboratory friction measurements of borehole-recovered fault gouge samples from the shallow high-slip region of the Tohoku-Oki earthquake^26^ and by laboratory constraints indicating that clay-rich lithified rock and rock gouges commonly exhibit velocity-strengthening to transitional behavior at low effective normal stress^43^. This assumption is intended as a generalized representation of shallow fault materials and does not imply that the entire shallow megathrust segment behaves as unlithified sediment. Additionally, off-fault plastic deformation dissipates seismic energy in the uppermost 10 km (Fig 6)^17^, though its overall contribution remains limited, accounting for only 2.9% of the total on-fault seismic moment. Lastly, our assumed initial stress state (Supplementary Fig. S3b) features low to negative shear stress near the trench, implying limited near-trench strain accumulation prior to the Tohoku-Oki earthquake^55^. Nonetheless, the collective effects of velocity-strengthening friction, off-fault plasticity, and low prestress conditions can only modestly reduce trench slip, by about 10% relative to the maximum slip further down-dip (Fig. 6). This reduction in trench slip aligns with near-trench bathymetric evidence of inelastic deformation and a decrease in horizontal displacement at the trench^56^.

### Spontaneous along-strike rupture arrest

**Fig. 7:**
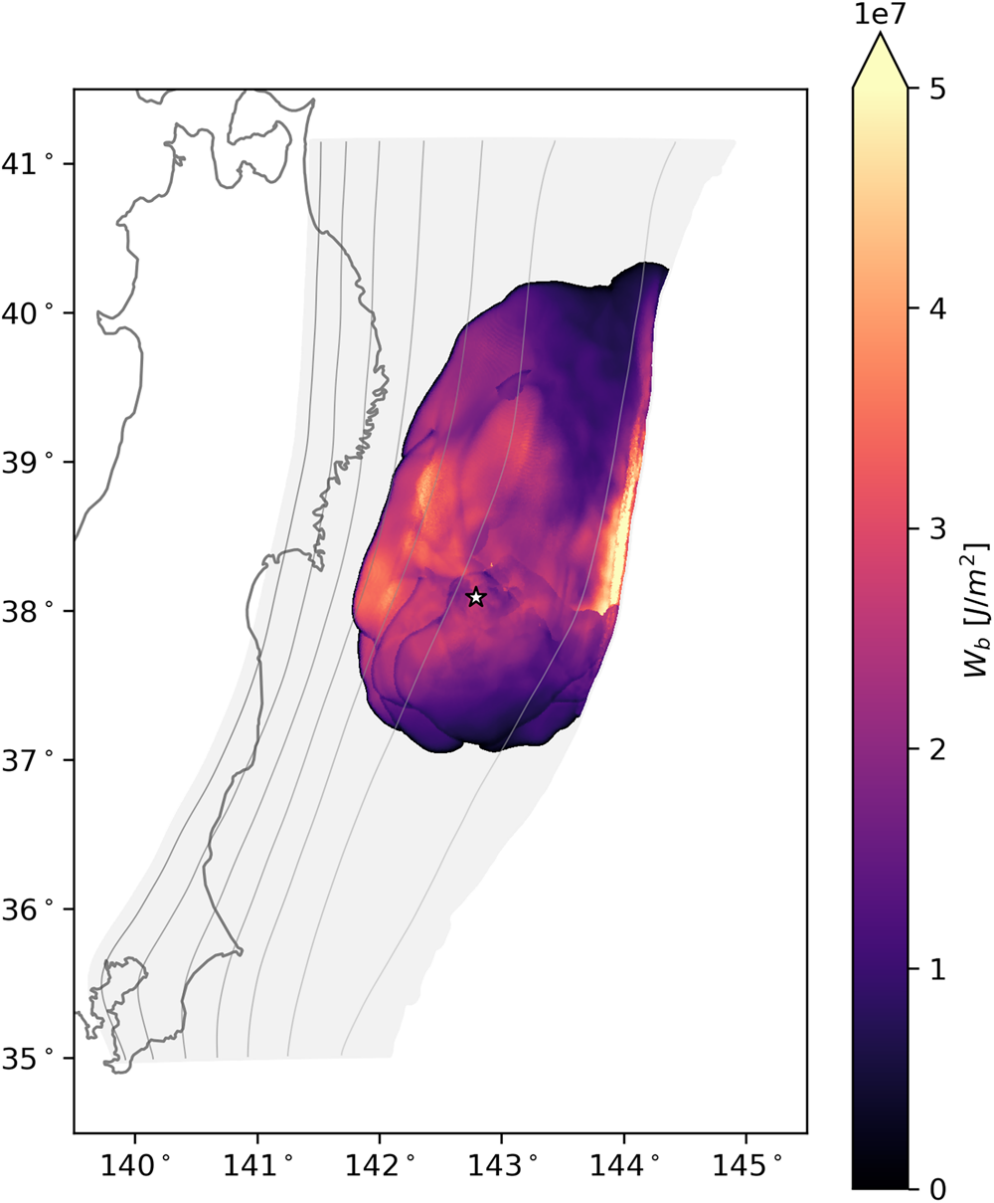
Breakdown work density ($W{^\prime_b}$, Methods Sec. “Breakdown work density”) distribution for the preferred dynamic rupture model. The preferred model shows pronounced spatial variation in breakdown work density, where downdip slip pulses increase the breakdown work density, compared to the updip region.

Our preferred model successfully reproduces the spontaneous along-strike rupture arrest of the Tohoku-Oki earthquake by incorporating data-informed stress heterogeneity rather than prescribing ad hoc frictional or structural barriers. In linear elastic fracture mechanics, dynamic rupture arrest occurs where available strain energy becomes insufficient to exceed local fracture energy, halting further rupture propagation^20,57^. In our simulation, rupture arrest results from strain energy depletion, as evidenced by a reduction in breakdown energy near the fault areas where rupture terminates (Fig. 7, Methods Sec. “Breakdown work density”). During the later stages of rupture (70–120 s), bilateral rupture pulses propagate into regions with lower relative prestress levels (*R*) (Fig. 1d), progressively decreasing in slip rate amplitude, rupture speed, and pulse width (Fig. 2d, e).

An exception occurs in the shallow northern slab section, where rupture arrest is delayed. Here, a localized region of elevated relative prestress, located at the northeast and updip of the hypocenter (Fig 1d), generates a high slip rate rupture pulse propagating toward the northern shallow margin (Fig. 3a). This interaction facilitates extended shallow rupture in the northern slab region, producing uplift patterns consistent with those derived from tsunami waveform inversions (Supplementary Fig. S8, e.g., ref. ^58^).

In contrast, alternative simulations that use solely depth-dependent prestress conditions informed by regional principal stress orientations without stress heterogeneities fail to spontaneously arrest rupture (Fig. 1b)^39^). Under comparable average prestress levels, these laterally homogeneous prestress models result in rupture of the entire megathrust, yielding an unrealistic moment magnitude of *M*_*W*_9.61 and a prolonged rupture duration of 220 s (Supplementary Fig. S14).

## Discussion

### Comparison of modeled rupture reactivation with observations

Strong-ground motion move-out patterns and finite-fault slip models of the Tohoku-Oki earthquake provide evidence for multiple rupture episodes^2–4,59^. Similar rupture complexities have been documented in other large megathrust earthquakes, including the 2010 *M*_*W*_ 8.8 Chile earthquake^60^, and the 2019 *M*_*W*_ 8.0 Northern Peru earthquake^53^.

**Fig. 8:**
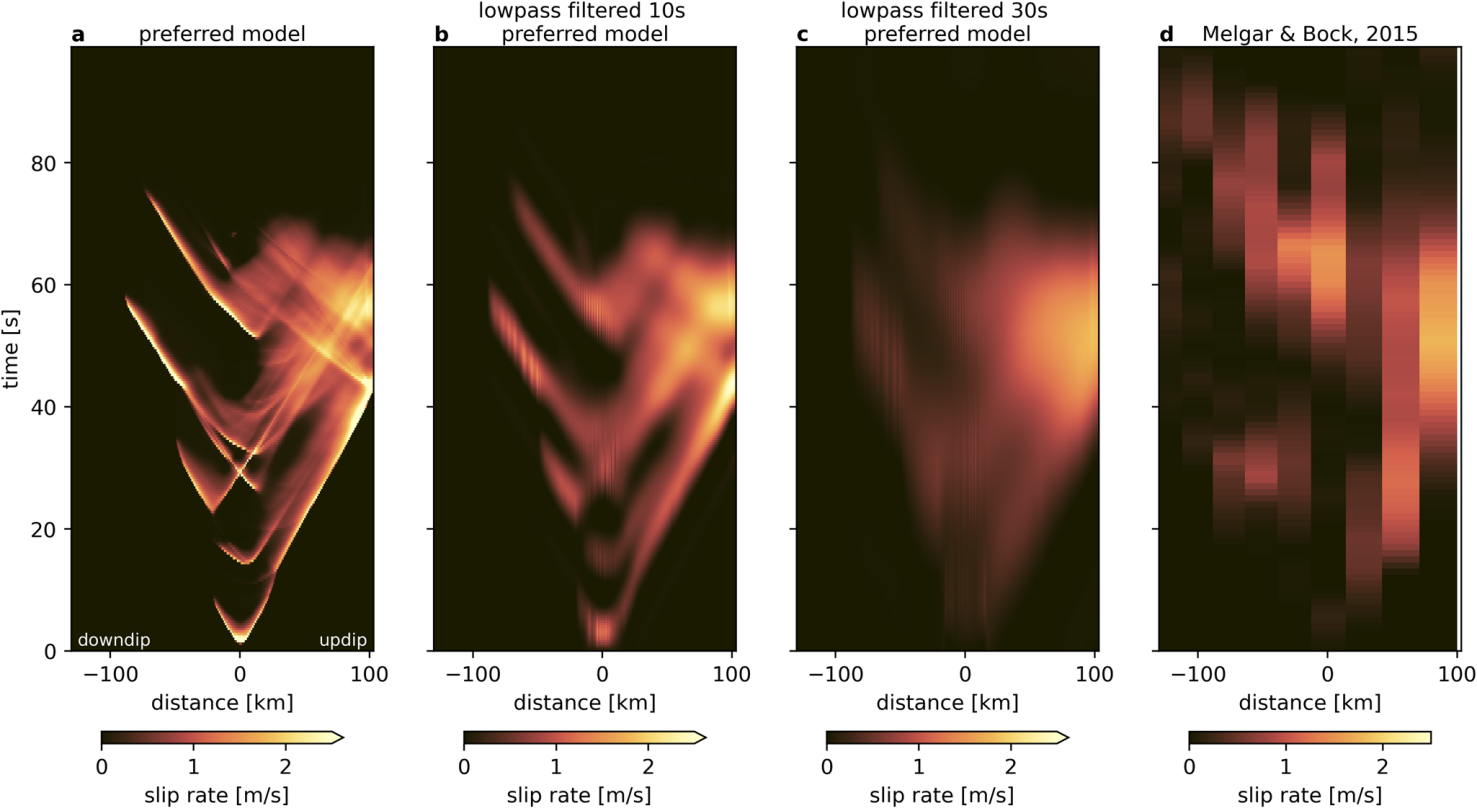
Comparison of slip-rate profile along a hypocentral dip profile between dynamic rupture models and the finite-fault model of ref. ^4^. **a** Along-dip slip-rate evolution of the preferred dynamic rupture model with heterogeneous prestress. **b** The same modeled slip-rate evolution low-pass-filtered at 10 s. **c** Modeled slip-rate evolution low-pass-filtered at 30 s period. **d** Along-dip slip-rate evolution of the ref. ^4^ finite-fault slip model. The profile is constructed from nine subfaults. Each subfault slip-rate function is represented by 20 triangular source time functions that are 10 s-long and 50%-overlapping .

Our simulations reproduce this first-order behavior, showing spontaneous rupture reactivation in the hypocentral region. However, our preferred model exhibits more reactivation episodes than the two to three inferred from most finite-fault models. These discrepancies likely reflect resolution limits in finite-fault inversions. The spectra of the slip-rate functions of the finite-fault models exhibit fast spectral decay starting at a period of 30 s^4^, whereas the slip-rate functions of our models show a more gradual spectral decay that starts between 10 and 30 s. Therefore, finite-fault models are likely to miss episodic, high-frequency slip pulses at depth. When low-pass filtered at 30 s, our simulated slip-rate evolution reproduces the reported patterns in ref. ^4^, including the updip rupture propagation during the first 50 s followed by a back-propagating rupture front between 50 and 90 s (Fig. 8). In addition, the preferred model reproduces key qualitative features of strong-motion records near the main rupture area, particularly the relative timing and spectral content up to 0.5 Hz, although long-period trends and absolute amplitudes are not fully captured (Supplementary Figs. S9–S10).

### Impact of prestress on dynamic rupture complexity

**Fig. 9:**
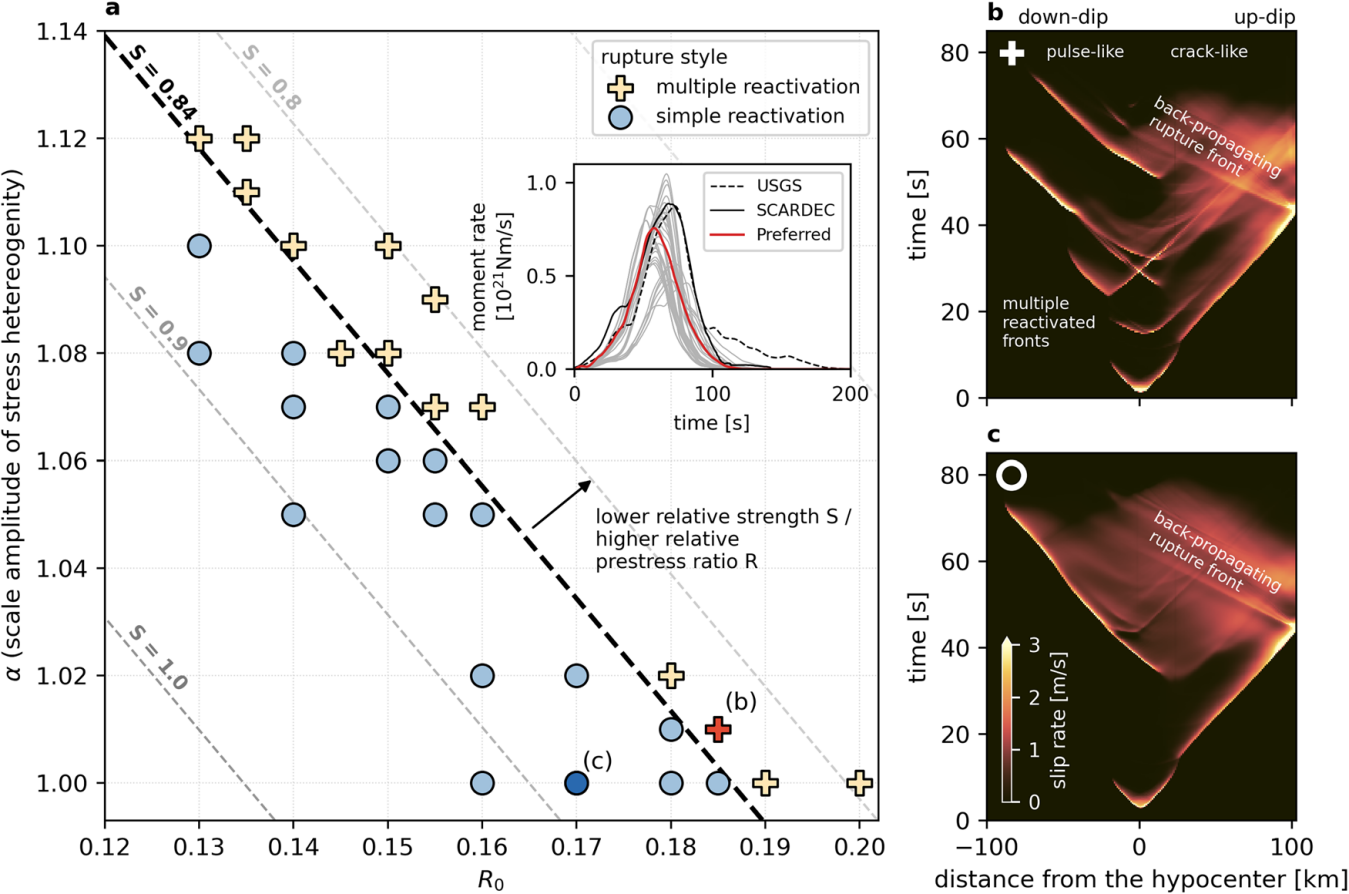
Distinct rupture styles under hypocentral friction and prestress. **a** Phase diagram showing how variations in stress heterogeneity amplitude (*α*) and regional ambient stress level (*R*_0_) control rupture style. Blue circles represent a family of dynamic rupture models dominated by prominent pulse-like rupture with free-surface reflection and back-propagating crack-like rupture, while yellow crosses denote models exhibiting repeated rupture reactivation near the hypocenter resembling the preferred model. The preferred model and an exemplary simpler model are indicated by the red cross and dark blue circle, respectively, with their corresponding along-dip slip-rate evolutions shown in (**b**, **c**), respectively. Dashed contours illustrate the variation of hypocentral seismic *S* ratio (initial strength excess over dynamic stress drop, Equation (16)), with the thick dashed line marking the transition boundary between rupture styles. We observe that the amplitude of stress heterogeneity (*α*) primarily controls peak slip magnitude, whereas the regional ambient stress level (*R*_0_) largely determines rupture extent in both families of dynamic rupture models. The inset compares the moment-rate functions of all models (gray lines), the preferred model (red), the USGS inversion^163^ (black dashed), and the SCARDEC inversion result^44^ (solid black). **b**, **c** Along-dip slip-rate evolution of the preferred model and exemplary simpler model, representing the rupture reactivation family and simpler rupture family, respectively.

Our preferred model indicates that the interplay of dynamically evolving friction and stresses gives rise to mixed rupture styles and depth-dependent rupture propagation. We next investigate how rupture evolution depends on the amplitude of stress heterogeneity and the relative prestress level by systematically varying *α* and *R*_0_, which represent the stress heterogeneity amplitude and regional ambient stress level. We find two distinct dynamic rupture styles (Fig. 9): one resembling the preferred model, and another dominated by a prominent updip-propagating pulse that reflects at the free surface, back-propagates, and transitions to crack-like rupture (Supplementary Video S2).

We find that our preferred rupture style requires low fault strength compared to the dynamic stress drop, corresponding to a low seismic *S* ratio^61^ (Equation (16), ratio of initial strength excess over dynamic stress drop, See Methods Sec."Prestress”). The *S* ratio measures the balance between fault proximity to failure and the available stress release during dynamic weakening. Lower effective normal stress and fault strength or higher initial shear stress favor reactivation-dominated scenarios. The abrupt transition in rupture behavior with small incremental changes in prestress is consistent with prior work on the interplay between evolving friction and stress (e.g., refs. ^27,62^). However, theoretical and numerical models generally predict that higher prestress downdip favors crack-like rupture, whereas lower prestress promotes updip pulse-like propagation. Consistent with the expectation, models without stress heterogeneity (Method Sec. “Regional stress rupture model without stress heterogeneity”) exhibit a pulse-to-crack rupture transition, characterized by a distinguishable rupture reactivation episode (Supplementary Figs. S15, S16, S17 and Supplementary Video S3).

In contrast, heterogeneous stress conditions in our models cause locally low prestress regions, particularly near the hypocenter and downdip, which facilitate the formation of self-healing pulse-like ruptures^62^. Subsequent slip arrest at depth reaccumulates shear stress along previously ruptured segments^42^, thereby enabling reactivation. All reactivated ruptures are pulse-like down-dip, due to the locally heterogeneous nature of the slip left behind by the primary rupture front, which correlates with the distribution of the primary residual stresses^27^. Updip, reactivated fronts coalesce with the free-surface reflected primary rupture to form a sustained crack-like rupture (Fig. 4a and Supplementary Fig. S5). Comparison with observed regional strong ground motion records shows that the preferred model with multiple reactivation shows multiple move-out branches, whereas the single reactivation model produces a single dominant pulse that is inconsistent with the observations (Supplementary Fig. S9).

### Origin and role of prestress heterogeneity

Defining initial stress conditions is a key challenge in dynamic rupture modeling. Prestress heterogeneity has been constrained in several ways, including using kinematic geodetic coupling models (e.g., ref. ^63^), Coulomb stress changes from preceding earthquakes or slow slip events (e.g., ref. ^64^), and coseismic slip distributions (e.g., ref. ^65^). Importantly, prestress heterogeneity does not uniquely determine dynamic rupture evolution, which is governed by the nonlinear interaction between frictional evolution, stress redistribution, and seismic wave propagation (e.g., ref. ^30^), rather than by static initial conditions alone, including prestress. The stress patterns inferred from finite-fault slip models capture large-scale stress variations associated with the preceding strain accumulation and coseismic stress release, but identical stress heterogeneities can lead to different rupture evolution, e.g., depending on frictional properties^65^.

### Rupture scenarios from alternative finite-fault slip models

To evaluate the effects of different prestress heterogeneity patterns, we explore dynamic rupture scenarios using three additional sets of prestress distributions derived from three finite-fault slip models:^4,66,67^. Melgar and Bock^4^ and Yamazaki et al.^66^ jointly invert seismic, geodetic, and tsunami data, whereas Kubota et al.^67^ invert using tsunami data only. In contrast to the median slip model, which features a single smooth slip patch, these slip models exhibit more heterogeneous slip. The Melgar and Bock^4^ and Yamazaki et al.^66^ models contain small-scale slip patches updip of the hypocenter, while the Kubota et al.^67^ model shows slip asperities in the southern region (Supplementary Figs. S24, S26, & S28). The corresponding stress-change patterns display greater heterogeneity and distinct spatial patterns.

Despite the contrasting prestress patterns, all alternative dynamic rupture models yield comparable rupture complexity, including multiple reactivation, large shallow slip near the trench, and spontaneous rupture arrest (Supplementary Figs. S24–S29, Videos S6–S8. Stronger prestress heterogeneity in the Melgar and Bock^4^ and Yamazaki et al.^66^ models produces more complex rupture evolution with multiple slip pulses across depth. For example, the Melgar and Bock^4^ model initiates with an updip slip pulse followed by reactivation updip of the hypocenter. Although these alternative models reproduce surface displacements well, they generate multiple peaks in moment-rate functions that are inconsistent with the observationally inferred triangular moment-rate function. In contrast, the preferred rupture model based on the median model of Wong et al.^10^ can dynamically reproduce the triangular moment-rate function.

Taken together, these alternative simulations also indicate that the key dynamic rupture complexity identified in our preferred model arises robustly from the underlying dynamic processes rather than from a particular choice of prestress distribution.

### Frictional heterogeneity

In addition to prestress heterogeneity, frictional heterogeneity is also an inherent part of the subduction megathrust heterogeneity. To compare the effects of these two types of heterogeneity, we conduct two complementary sets of dynamic rupture simulations introducing explicit frictional heterogeneity^12,65^, totaling ten models. In the following, we discuss alternative “preferred” scenarios for each ensemble, defined as the models that yield the highest variance reduction relative to the geodetic observations and the SCARDEC seismic moment-rate function^44^. These alternative models qualitatively agree with the preferred heterogeneous prestress model presented in the “Results” section. This highlights that similar dynamic rupture behavior can emerge from different forms of pre-existing heterogeneity in stress or frictional strength, when coupled with rapid frictional weakening and restrengthening.

**Fig. 10:**
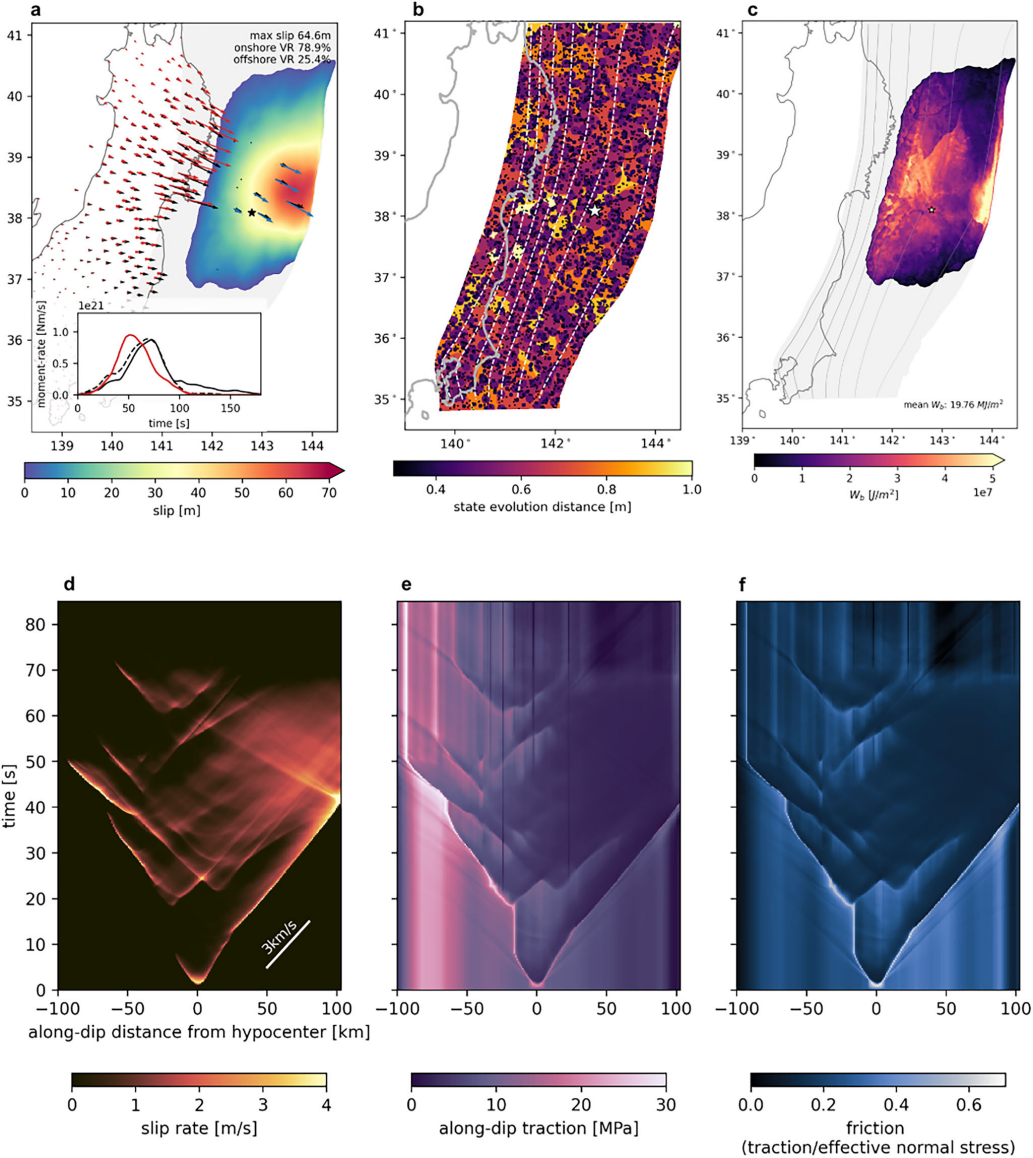
Dynamic rupture scenario incorporating heterogeneous state-evolution distance. **a** Simulated slip distribution and corresponding geodetic deformation. Red and blue arrows indicate synthetic onshore and offshore displacements, respectively. Black arrows show the observations. The inset compares the simulated moment-rate function (red), with the USGS (solid black), and SCARDEC (dashed black) source model moment-rate functions^163,164^. **b** Spatial distribution of the state-evolution distance *L* with multiscale heterogeneity ranging from 0.3 to 1.0 m. **c** Distribution of breakdown work density. **d**–**f** Along-dip profiles of slip-rate, along-dip traction, and effective friction coefficient evolution, from left to right, respectively. See also Supplementary Fig. S19 and Video S4.

In the first set of four simulations, we impose multiscale spatial variations in the state-evolution distance (*L*, Equation 5, and Methods: “Fault friction”), following the fractal parameterization of Ide and Aochi^68^ (Supplementary Fig. 10b). We superimpose heterogeneity in the *L* (0.3–1.0 m) onto the preferred model, which includes prestress heterogeneity. Despite this additional frictional heterogeneity, dynamic rupture evolution remains qualitatively consistent with the preferred model. We observe similar multiple reactivation episodes, mixed downdip pulse-like and updip crack-like rupture, and large shallow slip toward the trench (Fig. 10d). This heterogeneous friction model re-nucleates even more frequently than the depth-dependent friction case, exceeding the six reactivation episodes observed in the preferred model (Supplementary Fig. S19 and Video S4). It yields a breakdown work density comparable to the preferred model (19.8 MJ/m^2^ versus 19.6 MJ/m^2^), but a higher total radiated energy (10.1 × 10^17^*J* versus 7.7 × 10^17^*J*). The patches of shorter local nucleation length promote this frequent reactivation in the downdip and hypocentral regions.

The second set of six simulations maps heterogeneity inferred from the median slip distribution onto the fully-weakened frictional strength (${f}_{w}{\sigma }^{{\prime} }$, Equation 1, and Methods section: "Fault friction”), while maintaining a homogeneous, depth-dependent initial shear stress, following ref. ^65^ (Supplementary Fig. S21). Despite the contrasting assumptions about frictional heterogeneity, the heterogeneous-${f}_{w}{\sigma }^{{\prime} }$ preferred model reproduces key rupture complexities of the preferred model, including multiple reactivation, mixed rupture styles, and spontaneous arrest. However, unlike the preferred heterogeneous prestress model, depth-dependent rupture style variability is less clear, and rupture transitions into a pure crack-like style after 50 s simulation time (Supplementary Fig. S22) and arrests prematurely (Supplementary Figs. S22–S23 and Video S5). Because mapping heterogeneity into the fully-weakened friction coefficient decreases the local strength excess relative to the dynamic stress drop (Supplementary Fig. S20), the heterogeneous dynamic-friction model has a lower seismic *S* ratio and therefore tends toward crack-like rupture^27,62,69^. In contrast, the heterogeneous prestress model has a higher *S* ratio, which promotes self-healing pulse-like rupture and a more gradual arrest through decaying slip pulses (e.g., refs. ^42,57^).

### Comparison to previous models of the Tohoku-Oki earthquake

The observed penetration of slip into the velocity-strengthening near-trench region is consistent with the 2D dynamic simulations of ref. ^13^, which used classical rate-and-state friction and a realistic elastic structure including bimaterial contrasts. In their models, interaction with the free surface and enhanced shear loading from deeper slip sustain trench-breaching rupture despite velocity-strengthening friction. In our 3D simulations, enhanced frictional weakening and inelastic deformation reduce the near-trench peak slip by only about 10%, reproducing the large near-trench slip required by seafloor displacement measurements^7,8,56,70^. The agreement corroborates the potential for large coseismic displacements on shallow faults driven by strong coseismic weakening, despite the presence of velocity-strengthening friction and off-fault plastic deformation.

Our simulations differ from previous studies in the physical conditions required to obtain rupture complexity. Most previous Tohoku dynamic rupture models have relied on linear slip-weakening friction laws and prescribed asperities in stress or strength to promote multiple subevents and along-depth variability^14,16^. In contrast, our simulations employ a fast velocity-weakening rate-and-state friction law that incorporates rapid coseismic restrengthening and heterogeneous prestress constrained by observation-derived slip heterogeneity. Within this framework, multiple rupture reactivation and mixed pulse-like and crack-like behavior arise spontaneously from the co-evolution of friction, stress, and 3D fault geometry, without prescribing depth-dependent frictional patches or time-dependent reactivation schemes.

While our simulations reproduce pronounced downdip complexity and a systematic reduction of rise time at depth, back-projection studies of the 2011 Tohoku-Oki earthquake show comparatively weak high-frequency radiation from the shallow rupture^5,6^. Reconciling the discrepancy with our models may require additional mechanisms that may damp shallow peak slip-rates and high-frequency radiation, such as inelastic yielding of the accretionary wedge^17,38^ and depth-dependent frictional variations^14^.

### Limitations

We do not evaluate the effects of geometrical complexity and 3D velocity structure on rupture dynamics. Our simulations use a 1D velocity model and a smoothed megathrust geometry and therefore do not capture bimaterial contrasts across the plate interface or detailed upper-plate and sedimentary structure. Whereas these simplifications enable systematic exploration of how frictional and prestress parameters control dynamic rupture, additional processes proposed to influence rupture complexity in large megathrust earthquakes are not represented here, including along-strike variations in shallow material properties and sediment heterogeneity^71^, accretionary wedge geometry^72^, interface roughness and upper-plate structural heterogeneity^73^, bimaterial effects across the plate interface^13,74,75^, and spatial variations in pore-fluid pressure^76^. Incorporating these 3D effects is an important direction for future work.

The goal of this study is to identify dynamically viable rupture scenarios that reproduce the key source characteristics and illuminate the governing physical mechanisms, rather than to perform an inversion of all available observations. However, characterizing dynamic parameters, including shear stress and frictional properties, remains challenging because of strong trade-offs (e.g., ref. ^77^). Whereas this work focuses on coseismic dynamic rupture, future work may integrate observations across time-scales to help constrain the governing faulting conditions^78,79^.

Using 3D dynamic rupture simulations, we demonstrate that the complex rupture behavior of the Tohoku-Oki earthquake can spontaneously arise from dynamic rupture processes. We highlight that fault heterogeneity, although challenging to quantify, can be informed by existing observational data and fundamentally controls the complexity and scale of dynamic rupture. Our models capture episodic re-nucleation in the hypocentral region, the co-existence of short-duration, spiraling slip pulses at depth and shallow crack-like rupture with large slip near the trench, and spontaneous rupture arrest. To this end, this work provides a robust, self-consistent framework applicable to other megathrust settings towards physics-based earthquake and tsunami hazard assessment worldwide.

## Methods

We perform 3D dynamic rupture simulations of the 2011 *M*_*w*_ 9.0 Tohoku-Oki earthquake that simultaneously solve for seismic wave propagation, on-fault frictional failure, and off-fault inelastic deformation. 2D and 3D dynamic rupture simulations have been applied to subduction zones worldwide (e.g., refs. ^38,63,80–85^), including the Tohoku-Oki earthquake^11–14,16,17^. We use the open-source software SeisSol (https://seissol.org) for all dynamic rupture simulations on two supercomputers, SuperMUC-NG at the Leibniz Supercomputing Center, Germany, and Frontera at the Texas Advanced Computing Center, United States. SeisSol employs the Arbitrary High-order Derivative (ADER) Discontinuous Galerkin (DG) method^86^, which enables higher-order accuracy in space and time on unstructured tetrahedral meshes, which are well-suited to capture geometric complexities, including shallowly dipping megathrust interfaces in subduction zones [e.g., refs. ^38,82^]. SeisSol is optimized for high-performance computing [e.g., refs. ^37,87,88^] and verified in dynamic rupture community benchmarks^89–92^. We employ SeisSol with sixth-order accuracy in time and space, i.e., the polynomial order of the basis functions is *p* = 5.

### Model geometry and mesh

Our 3D dynamic rupture models incorporate realistic megathrust geometry, high-resolution topobathymetry, and velocity-aware adaptive mesh refinement to accurately capture rupture processes and seismic wave propagation up to 2 Hz^93^. Our megathrust geometry is adapted from the 3D Japan Integrated Velocity Structure Model geometry (JIVSM)^94,95^, which is based on seismic imaging, waveform inversion, and seismicity studies. We extract the top layer of the oceanic plate as the megathrust interface. To ensure the interface connects to the trench, we extend and smooth the interface to the USGS trench with a 12° extension from the surface following^10^. The constructed megathrust interface spans a region from 35°N to 41°N and from 139.5°E to 145°E, extending approximately 700 km along strike, 250 km along dip, and reaching a depth of 80 km (Fig. 1). This large extent of the megathrust geometry can prevent model boundary effects on dynamic rupture arrest.

Our model incorporates realistic topobathymetry using the Geobco dataset at 15 arcs (380 m) resolution^96^. We use a 1D velocity structure consisting of five layers, which we modify from ref. ^97^ (Supplementary Table S2) by prescribing a 20% shear modulus reduction in the two uppermost layers. This adjustment accounts for the presence of lower-rigidity materials in shallow subduction zone regions and closely matches the average shear modulus derived from the 3D JIVSM velocity model at equivalent depths.

Our structure model is refined near the fault interface to accurately resolve the process zone at the rupture tip, near the free surface to capture topobathymetry, and near Honshu Island to resolve the seismic wavefield up to 1.5 Hz (Supplementary Sec.: “SM: Model domain and resolution,” Fig. S10). The process zone width Λ^98^ is defined as the area behind the rupture front in which the shear stress decreases from the static value to the dynamic value. The resulting unstructured tetrahedral mesh consists of 50.3 million elements, and one simulation requires 58,000 CPU hours on Frontera and 64,000 CPU hours on SuperMUC-NG. The mesh uses the following Cartesian projection: WGS84/UTM transverse mercator centered at (143°E, 39°N).

### Fault friction

We use a fast velocity-weakening rate-and-state friction law that replicates the severe coseismic friction reduction observed in high slip-rate laboratory experiments^25,99^, including studies of using drilled samples from the Japan subduction zone^26,100^. Such pronounced weakening at elevated slip rates can result from flash heating of highly stressed, short-lived contact asperities and thermal pressurization due to shear heating of pore fluids [e.g., refs. ^40,41,101,102^].

This friction law allows the megathrust fault interface to operate under low average shear stress while producing realistic fault slip and stress drop during dynamic rupture^24,28^, and promoting complex rupture styles including cascading ruptures across multi-fault systems [e.g., refs. ^27,76,103–107^]. Low average shear stress conditions align with the limited thermal signature of the Tohoku-Oki earthquake, which may imply rupture under low ambient stress levels^108^. Although this friction law has been extensively used in 2D simulations to examine its control on rupture dynamics [e.g., refs. ^24,109,110^] and in 3D models of crustal earthquakes [e.g., refs. ^28,78^], it has not yet been explored in a 3D full-scale model of a large megathrust earthquake.

We use the formulation suggested in the community benchmark problem TPV104 of the Southern California Earthquake Center^91^, which is similar to the friction law introduced by ref. ^109^. All frictional parameters are listed in Supplementary Table S1.

In the rate-and-state friction framework, frictional strength depends on both the state of the slipping surface and the current slip rate^22,23,111^. The shear traction *τ*, is assumed to equal fault strength, and is given by

3 $$\tau=f(V,\theta ){\sigma }_{n}^{{\prime} }.$$

*f* is the effective friction coefficient, *V* is the slip rate, *θ* is the state variable, and ${\sigma }_{n}^{{\prime} }$ is the effective normal stress.

The frictional coefficient *f* depends on *V* and *θ*, as

4 $$f(V,\theta )=a \; {\sinh }^{-1}\left[\frac{V}{2{V}_{0}}\exp \left(\frac{\theta }{a}\right)\right],$$

where *a* is the direct-effect parameter, and *V*_0_ is the reference velocity. The evolution of *θ* is governed by

5 $$\frac{d\theta }{dt}=-\frac{V}{L}(\theta -{\theta }_{ss}),$$

where *L* is the characteristic slip distance, *t* is time, and *θ*_*ss*_ is the steady-state value of the state variable, which is given by

6 $${\theta }_{ss}(V)=a \; \ln\left[\frac{2{V}_{0}}{V}\sinh \left(\frac{{f}_{ss}V}{a}\right)\right].$$

The steady-state friction coefficient *f*_*ss*_ is given by

7 $${f}_{ss}(V)={f}_{w}+\frac{{f}_{L{V},{ss}}(V)-{f}_{w}}{{(1+{(V/{V}_{w})}^{4})}^{1/4}},$$

where *V*_*w*_ is the onset of the weakening velocity, *f*_*w*_ is the fully weakened friction coefficient, and the steady-state low-velocity friction coefficient is:

8 $${f}_{L{V},{ss}}={f}_{0}-(b-a)\; \ln(V/{V}_{0}),$$

with *b* as the state-evolution parameter, and *f*_0_ as the reference friction coefficient. The steady-state friction behavior is asymptotic, such that ${f}_{ss}(V)\approx {f}_{L{V},{ss}}(V)\,{{{\rm{for}}}}\,V\ll {V}_{w}$ and *f*_*ss*_(*V*) ≈ *f*_*w*_ for *V* ≫ *V*_*w*_. This behavior aligns with laboratory observations, capturing classic rate-and-state frictional behavior at low sliding velocities and pronounced frictional weakening at high sliding velocities.

Velocity-strengthening, (*a* − *b*) > 0, friction describes materials whose frictional strength increases with rising slip rate, thus stabilizing fault slip. Conversely, velocity-weakening, (*a* − *b*) < 0, friction characterizes materials that decrease in strength with increasing slip rate, facilitating the nucleation and propagation of unstable slip^19,112^.

In our models, we prescribe a depth-dependent distribution of (*a* − *b*) to represent realistic frictional behavior along the megathrust interface. The shallow portion ( < 10 km depth) of the Japan subduction zone is characterized by velocity-strengthening friction, consistent with laboratory measurements of frictional behavior of clay-rich accretionary wedge material at low slip velocities^26,43,113,114^. This velocity-strengthening region is constrained by the transition depth from accretionary wedge to bedrock, as defined by the JIVSM^94,95^. Between 10–45 km depth, we define the seismogenic zone by parameterizing velocity-weakening friction. Further downdip, between 40–50 km depth, friction gradually transitions back to velocity-strengthening, consistent with observed downdip limits of seismicity and diverse faulting behavior^31,33^. We assign uniform frictional parameters along strike within each depth interval, except near the hypocenter, where a modified state evolution distance is imposed for smooth rupture nucleation (Supplementary Section “SM2: Nucleation”). We emphasize that our assumptions of depth-dependent frictional properties do not imply a frictionally homogeneous megathrust. Our assumptions on depth-dependent frictional properties are sought to explore rupture dynamics driven solely by dynamic processes and without introducing ad-hoc frictional asperities and barriers. We acknowledge that the Japan trench subduction zone hosts diverse faulting behavior, including slow-slip events, tremors, low-frequency earthquakes, and moderate-to-large thrusting earthquakes^33^.

### Depth-dependent effective normal stress

Pore fluid pressure plays an important role in controlling the effective normal stress and thus the shear stress conditions governing earthquake rupture dynamics [e.g., ref. ^115^]. Drilling observations and seismic reflection studies [e.g., refs. ^116,117^] as well as stress orientation analyses^118^ suggest elevated pore-fluid pressure in the Japan subduction zone, with measurements within the accretionary wedge and along the shallow fault interface reaching 80–95% of lithostatic stress. Drilling observations and seismic reflection studies in the Japan subduction zone have documented elevated pore fluid pressure within the accretionary wedge and along the shallow fault interface, reaching 80–95% of lithostatic stress^116,117^. Stress orientation analyses support elevated ambient pore fluid pressure at seismogenic depths^119^. We assume that pore fluid pressure reaches 90% of lithostatic stress in all layers. The lithostatic stress is defined as ${P}_{litho}(z)={\int }_{0}^{z}({\rho }_{i}g{h}_{i})dz$, where the subscript *i* refers to the respective layer in the velocity model (Supplementary Table S2) and *g* = 9.81*m*/*s*^2^ is the gravitational acceleration. This assumption results in depth-dependent effective normal stress and relatively low fault strength everywhere (Supplementary Fig. S3c).

### Off-fault plasticity

We account for off-fault inelastic energy dissipation using a Drucker-Prager visco-elasto-plastic rheology^120,121^. Models incorporating off-fault plasticity require specifying initial stress, bulk friction, and cohesion throughout the entire simulation domain. We employ a depth-dependent cohesion model following^38^ and motivated by laboratory-inferred shallow low cohesion^43,113^, where bulk cohesion *C*(*z*) varies linearly with effective confining pressure:

9 $$C(z)={C}_{0}+{C}_{1}(z){\sigma }_{c}^{{\prime} },$$

where *C*_1_(*z*) represents rock hardening with depth and ${\sigma }_{c}^{{\prime} }={\sigma }_{litho}-{P}_{f}$ is the effective confining stress. We set *C*_0_ to 1.0 MPa to represent partially consolidated sediments, while *C*1 linearly reduces from 1 to 0 at depths shallower than 18 km.

The Drucker-Prager yield criterion is given by

10 $${\tau }_{c}=C(z)\cos (\Phi )-{\sigma }_{m}\sin (\Phi ),$$

where $\Phi=\arctan (f^{\prime} )$ is the internal angle of friction and ${\sigma }_{m}={\Sigma }_{n=1}^{3}{\sigma }_{ii}/3$ as the mean stress.

The closeness-to-failure (CF) metric^122^ is defined as the ratio between the magnitude of the deviatoric shear stress (*J*_2_) and *τ*_*c*_:

11 $$CF=\frac{\sqrt{{J}_{2}}}{{\tau }_{c}}.$$

This parameterization results in shallow regions (above 10 km depth) being close to yielding (*CF* ≈ 0.8) under both preferred and regional stress conditions (Supplementary Fig. S11).

The total seismic moment *M*_0,*t*_ is the sum of the moment due to the slip on fault, *M*_0,*e*_, and the moment due to off-fault plastic strain, *M*_0,*p*_^38,123^, as:

12 $${M}_{0,p}={\sum}_{i=1}^{N}\mu V\eta,$$

where *μ* is the rigidity, *V* is the volume of each tetrahedral element *i*, and *η* is a scalar quantity measuring the accumulated off-fault plastic strain at the end of the dynamic rupture simulation. Following ref. ^124^, *η* is defined as:

13 $$\eta (t)={\int }_{0}^{t}\sqrt{\frac{1}{2}{\dot{\epsilon }}_{ij}^{p}{\dot{\epsilon }}_{ij}^{p}}dt,$$

with ${\dot{\epsilon }}_{ij}^{p}$ as the 3D inelastic strain rate tensor.

The contribution of plastic strain to the total moment is small for our rupture models. Ratios of *M*_0,*p*_/*M*_0,*e*_ for both the heterogeneous and regional relative prestress rupture scenarios are on the order of a few percent (preferred model: 2.9%), consistent with 2D dynamic-rupture simulations and large-scale megathrust simulation^38,123,125^ at comparable relative prestress levels.

### Prestress

Variations in prestress and fault strength significantly influence rupture style and complexity^27,62,69,109^. However, the prestress and strength conditions that govern earthquake rupture are challenging to directly constrain by observations^46,126^. Multiple approaches have been proposed to parameterize friction and/or stress conditions from locking models^80,83,127,128^, finite-fault slip distributions^65,129–132^, and stress change from prior events^85,133^. Previous dynamic rupture models of the Tohoku-Oki earthquake often assume rupture complexity from frictional properties and have relied on prescribed frictional asperities [e.g., refs. ^12,17,76^] and, in some cases, additional stress asperities [e.g., refs. ^11,14,16,134–136^], both of which require ad-hoc assumptions.

Here, we use a data-informed framework to explore the prestress conditions. We define the initial stress tensor *s*_*ij*_ as a linear combination of the regional-tectonically constrained stress tensor *b*_*ij*_ and the stress changes inferred from finite-fault slip models *c*_*ij*_, following^131,137^. The initial full stress tensor *s*_*ij*_ is defined as:

14 $${s}_{ij}(x,y,z)=\Omega (z)({b}_{ij}(x,y,z)+\alpha {c}_{ij}(x,y,z))+(1-\Omega (z)){\sigma }_{n}^{{\prime} }(x,y,z){\delta }_{ij},$$

with *Ω*(*z*) as a depth-dependent modulation function smoothly tapering deviatoric stresses below the seismogenic zone (45 km), *α* as a scaling factor controlling the amplitude of stress heterogeneity, and *δ*_*ij*_ is the Kronecker Delta.

Dynamic rupture simulations often exhibit strong trade-offs between friction and initial stress conditions [e.g., ref. ^65^]), which can be characterized by the relative prestress ratio *R* between the maximum potential stress drop and frictional strength drop^138^. Following ref. ^28^, to define *R* in our velocity-weakening rate-and-state friction framework, we approximate peak shear strength as ${f}_{0}{\sigma }_{n}^{{\prime} }$ and residual strength as the fully weakened frictional state, ${f}_{w}{\sigma }_{n}^{{\prime} }$. During rupture, the shear stress level typically approaches this fully weakened frictional state (Supplementary Fig. S5). *R* is then defined as

15 $$R=\frac{{\tau }_{0}-{\mu }_{d}{\sigma }_{n}^{{\prime} }}{({\mu }_{s}-{\mu }_{d}){\sigma }_{n}^{{\prime} }}\approx \frac{{\tau }_{0}-{f}_{w}{\sigma }_{n}^{{\prime} }}{({f}_{0}-{f}_{w}){\sigma }^{{\prime} }s},$$

where *τ*_0_ is the initial shear traction projected from *s*_*ij*_ on the 3D megathrust interface.

Alternatively, initial stress and fault strength can be characterized by the seismic ratio *S*^61^, which represents the ratio of initial strength excess to maximum dynamic stress drop:

16 $$S=\frac{{\mu }_{s}{\sigma }_{n}^{{\prime} }-{\tau }_{0}}{{\tau }_{0}-{\mu }_{d}{\sigma }_{n}^{{\prime} }}\approx \frac{{f}_{0}{\sigma }_{n}^{{\prime} }-{\tau }_{0}}{{\tau }_{0}-{f}_{w}{\sigma }_{n}^{{\prime} }},$$

with a direct relationship between *R* and *S*:

17 $$R=\frac{1}{1+S}.$$

The *R* and *S* ratios capture different aspects of the balance between available strain energy and fracture energy, thus influencing dynamic stress drop and acceleration or deceleration of the rupture front. For non-planar fault geometries and spatially variable prestress and initial fault strength, these ratios vary across the fault interface(s). As detailed below (Methods Sec.: “Ambient Prestress”), prescribing a regionally uniform *R*_0 _≥ *R*, defined as the *R*-value for an optimally oriented fault segment, allows us to constrain the amplitude of deviatoric stresses relative to the frictional strength drop, while naturally incorporating stress variability due to the megathrust geometry.

#### Ambient prestress

The ambient prestress tensor *b*_*ij*_ is constrained using observed regional stress orientations, and assumed fault-fluid pressure and Mohr-Coulomb frictional failure criteria, following^38^. We prescribe a uniform regional stress field orientation based on the inferred principal stress orientations along the Japan subduction zone from the World Stress Map^39^, with the maximum principal stress oriented at an azimuth of 100° and a plunge angle of 8°. The magnitudes of the principal stresses *s*_*i*_ are determined through the stress shape ratio *ν* as:

18 $$\nu=\frac{{s}_{2}-{s}_{3}}{{s}_{1}-{s}_{2}}.$$

We use *ν* = 0.5 in all simulations, again based on the World Stress Map^39^.

Following the notation of ref. ^138^, the Mohr-Coulomb failure criteria is defined as:

19 $$P=({s}_{1}+{s}_{3})/2\,\;\;{{{\rm{and}}}}\;\;\,ds=({s}_{1}-{s}_{3})/2.$$

with (*P*, 0) being the center of the Mohr-Coulomb circle and *ds* as its radius. Principal stresses *s*_*i*_ are related to *P*,  *ds* and *ν* as

20 $${s}_{1}=	 P+ds,\\ {s}_{2}=	 P-ds+2\nu ds,\\ {s}_{3}=	 P-ds.$$

The effective mean confining stress ${\sigma }_{c}^{{\prime} }=({s}_{1}+{s}_{2}+{s}_{3})/3$ is given by:

21 $${\sigma }_{c}^{{\prime} }=P+(2\nu -1)ds/3.$$

The shear and normal stresses (*τ* and *σ*_*n*_) acting on a fault plane oriented at an angle *Φ* relative to the maximum principal stress are:

22 $$\tau=	 ds\sin 2\Phi,\\ {\sigma }_{n}=	 P-ds\cos 2\Phi,$$

In this framework, an optimally oriented fault plane is defined as the orientation that, under uniform initial stress and loading rate, reaches frictional failure first, maximizing the shear-to-normal stress ratio to equal the static friction coefficient *μ*_*s*_. Its optimal orientation relative to the maximum principal stress direction is thus:

23 $$\Phi=\pi /4-0.5\arctan ({f}_{0}{\sigma }_{n}^{{\prime} }).$$

The deviatoric stress magnitude *ds* is derived by combining Eqs. (15), (21), and (22):

24 $$ds=\frac{{\sigma }_{c}^{{\prime} }}{\sin 2\Phi /({f}_{w}+({f}_{0}-{f}_{w}){R}_{0})+(2\nu -1)/3+\cos 2\Phi }\,.$$

Based on a given regional optimal relative prestress ratio *R*_0_, we can compute the principal stress amplitude *s*_*i*_ using Eqs. (20), (21), and (24). The orientations of the principal stress axes are constrained by the azimuth *SH*_*max*_ = 100° and the plunge angle *θ* = 8°. We systematically vary *R*_0_ from 0.1 to 0.2 to identify the preferred rupture model. This range corresponds to an initial shear stress of 3.12-4.05 MPa at hypocentral depth.

#### Data-informed shear stress heterogeneity

Prestress heterogeneity may arise from past rupture on the same or nearby fault, unmodelled fault geometrical complexities, local variations of fault strength or pore-fluid pressures, or variations in tectonic loading. The coseismic slip distributions reflect such heterogeneities and have therefore been widely used to constrain the initial stress distribution for dynamic models (e.g., refs. ^65,131,132,139^).

Here, we adopt the median slip distribution derived from 32 published finite-fault models of the Tohoku-Oki earthquake^10^ to inform the heterogeneity pattern. This median slip model has a simple slip distribution with a smooth, circular patch up-dip of the hypocenter, showing significant slip extending to the trench. The model robustly captures large-scale slip features common across these models and can successfully reproduce key geodetic and seismic observations when combined with appropriate slip-rate functions. We compute volumetric stress tensor changes *c*_*ij*_ resulting from this imposed slip distribution on the megathrust interface using SeisSol in a dynamic relaxation calculation^65,128^, utilizing the same computational mesh and slab geometry as in our subsequent dynamic rupture simulations. We impose a regularized Yoffe slip-rate function as an internal boundary condition to compute the stress changes across the slab interface. This approach leverages the discontinuous finite-element discretization of SeisSol, accurately capturing displacement discontinuities along the fault interface. We perform dynamic relaxation for 200 s, sufficient for all seismic waves to exit the computational domain and achieve steady-state stress conditions. In contrast to previous methods, which used finite-fault slip models primarily to estimate fault-interface stresses [e.g., refs. ^127,140–142^], our calculation simultaneously estimates both fault-interface and surrounding volumetric stress changes. High slip gradients can lead to unrealistic stress concentrations, particularly in shallow regions. To mitigate this, we include inelastic off-fault plastic yielding during the dynamic relaxation step, employing the same parameters as during dynamic rupture simulations (Methods Sec.: “Off-fault plasticity”). The resulting stress changes on the megathrust interface are shown in Supplementary Fig. S2.

### Regional stress rupture model without stress heterogeneity

When only using the regionally constrained stress tensor *b*_*ij*_ (i.e., *α* = 0), we obtain a laterally homogeneous prestress model with a uniform relative prestress ratio *R* across the entire megathrust (Fig. 1b and Supplementary Fig. S3a). This homogeneity results from the principal stress orientations and overall geometry of the Japan subduction zone being largely uniform along strike. To systematically explore dynamic rupture scenarios without imposed stress heterogeneity, we vary the regional relative prestress level *R*_0_ within the range 0.56-0.64 in increments of 0.02, consistent with the average relative prestress value within the rupture area of the preferred model (Fig. 1d).

In all five homogeneous stress scenarios, dynamic rupture propagates along the entire megathrust interface. The rupture model with *R*_0_ = 0.58 yields an unrealistic magnitude of *M*_*w*_ 9.61 and an extended rupture duration of 180 s (Supplementary Fig. S14). In contrast to the preferred model, the laterally homogeneous prestress model exhibits crack-like rupture reactivation (Supplementary Fig. S15, Fig S17, and Video S2), occurring at the downdip healing front of the growing pulse. This simulation does not reproduce the distinct updip and downdip rupture propagation speeds and complex rupture evolution documented in back-projection studies^5,143–145^ (Supplementary Fig. S15b).

### Breakdown work density

Breakdown work is defined as the frictional work that provides an estimate of the irreversible part of the total strain energy change, which does not go into radiated energy^30^. The breakdown work combines fracture energy and restrengthening work^141,146,147^,^148–157^. Since multiple rupture episodes occur during most of our simulations, we sum the breakdown work of each rupture episode into the total breakdown work *W*_*b*_ (red-shaded areas in Supplementary Fig. S5). We then define the breakdown work density $W{^\prime_b}$ per unit area, defined as the excess of work over the minimum shear stress level achieved during total slip:

25 $${W}_{b}^{{\prime} }={\int }_{0}^{{t}_{f}}(\tau (t)-{\tau }_{min})\dot{\delta }(t)dt,$$

where $\dot{\delta }(t)$ is the slip velocity and *t*_*f*_ is the end time of the rupture defined as the absolute slip-rate decrease less than 0.01*m*/*s*. The calculated breakdown work density of the preferred model is shown in Supplementary Fig. 7. The breakdown work density exhibits significant spatial variability and depends on the rupture process^158^. Multiple slip pulses downdip and in the hypocentral region generally increase the breakdown work density, compared to updip regions. The average breakdown work density of the preferred model is 19.6 *MJ*/*m*^2^, consistent with the estimated and expected average breakdown work density for an *M*_*w*_ 9 event^6,102,146^.

### Dynamic rupture scenarios with prestress heterogeneity informed by alternative finite-fault slip models

We map the heterogeneity from each kinematic slip model to the initial stress distribution using the same approach as in the preferred model, and retain the same friction parameterization. We vary the amplitude of initial stress heterogeneity (*α*) and the ambient regional stress, expressed by the relative prestress level (*R*_0_). To save computational cost, we run six simulations per prestress heterogeneity distribution. For each ensemble, the preferred scenario is chosen by maximizing the variance reduction relative to the geodetic observations and SCARDEC seismic moment-rate^44^. The corresponding three alternative dynamic rupture model results are presented in Supplementary Figs. S24–S29.

## Supplementary information

Supplementary Information

Description of Additional Supplementary File

Supplementary Video S1

Supplementary Video S2

Supplementary Video S3

Supplementary Video S4

Supplementary Video S5

Supplementary Video S6

Supplementary Video S7

Supplementary Video S8

Transparent Peer Review file

## Data Availability

All data and model parameter files required to reproduce the dynamic rupture scenarios can be downloaded from https://zenodo.org/records/17382787. The onshore geodetic data are provided by the Geospatial Information Authority (GSI)^160^. The K-NET strong-ground motion data is provided by the National Research Institute for Earth Science and Disaster Resilience, Japan^161^.

## Code availability

We use the open-source software package SeisSol version 1.2.0^162^, available at https://github.com/SeisSol/SeisSol, commit 8*c**d**d**b*43*c* on the master branch, to perform all dynamic rupture simulations presented in this study. Instructions for downloading, installing, and running the code are available in the SeisSol documentation at https://seissol.readthedocs.io/.
