# Supplementary material for: Dynamic restrengthening and fault heterogeneity explain megathrust earthquake complexity
Source: Nat Commun. 2026 Apr 27;17:5777. doi: 10.1038/s41467-026-71722-3 (PMC13323747; doi:10.1038/s41467-026-71722-3)
Supplement: Supplementary file 1 — Supplementary Information [file 41467_2026_71722_MOESM1_ESM.pdf]

Supplementary Information

Dynamic restrengthening and fault heterogeneity explain megathrust earthquake complexity

Jeremy Wing Ching Wong[1*], Alice-Agnes Gabriel[1,2], Wenyuan Fan[1]

[1] Institute of Geophysics and Planetary Physics, Scripps Institution of Oceanography, University of California San Diego, La Jolla, CA, USA

[2] Department of Earth and Environmental Sciences, Ludwig-Maximilians-Universität München, Munich, Germany

[*] Correspondence to: jeremywong@ucsd.edu

March 11, 2026

Supplementary Information

List of supplementary figures, videos and corresponding simulations.

- Preferred dynamic rupture model with prestress heterogeneity:
  - Suppl. Fig. S1: Median slip distribution computed from 32 finite-fault slip distributions of the Tohoku-Oki earthquake compiled by [10] and projected onto our new slab geometry.
  - Suppl. Fig S2: Stress changes resulting from the median finite-fault slip distribution on the megathrust interface.
  - Suppl. Fig. S3d-e: Initial shear stress and effective normal stress distributions along the megathrust interface.
  - Suppl. Fig. S4: Dynamic rupture evolution of the preferred model with slip rate evolution snapshots.
  - Suppl. Fig. S5: Temporal evolution of stress, frictional strength, and slip rate at updip, hypocentral, and downdip regions.
  - Suppl. Fig. S6: Comparison of the peak-slip-rate distributions across three frequency ranges.
  - Suppl. Fig. S8: Simulated seafloor displacement field.
  - Suppl. Fig. S9: Comparison of observed and synthetic regional strong-ground motion records.
  - Suppl. Fig. S10: Velocity spectra of modeled waveforms at onshore strong-motion K-net stations using the preferred model.
  - Suppl. Fig. S11: Depth-dependent cohesion (red) and closeness-to-failure profiles.
  - Suppl. Video S1: Video of slip rate, along-dip shear stress, and friction.
- Dynamic rupture model with prestress heterogeneity and simple reactivation rupture style:
  - Suppl. Fig. S9: Regional strong-ground motion comparison.
  - Suppl. Fig. S12: Comparison of moment-rate functions for two distinct rupture styles shown in Figure 9.
  - Suppl. Fig. S13: Comparison of onshore and offshore geodetic displacement misfits across models with varying prestress heterogeneity amplitude α and regional relative prestress level R_0.
  - Suppl. Video S2: Video of slip rate, along-dip shear stress, and friction.
- Dynamic rupture model with homogeneous regional prestress condition:
  - Suppl. Fig. S3a-b: Initial shear stress conditions.
  - Suppl. Fig. S14: Comparison of fault slip distributions.
  - Suppl. Fig. S15: Slip rate, friction, and shear stress evolution along the hypocentral dip profile.

  - Suppl. Fig. S16: Depth-dependent slip rate evolution and corresponding amplitude spectra.
  - Suppl. Fig. S17: Slip rate evolution snapshots.
  - Suppl. Video S2: Video of slip rate, along-dip shear stress, and friction.
- Dynamic rupture model with heterogeneous prestress and uniform state-evolution distance:
  - Suppl. Fig. S18: Alternative dynamic rupture model using a uniform weakening distance L of 0.3 m.
- Dynamic rupture model with prestress heterogeneity and multiscale heterogeneity in the state-evolution distance:
  - Fig. 10: Model setup and overview.
  - Suppl. Fig. S19: Slip-rate evolution of the heterogeneous-friction dynamic rupture model with multiscale variations in state-evolution distance.
  - Suppl. Video S4: Video of slip rate, along-dip shear stress, and friction.
- Dynamic rupture model with heterogeneous distribution of fully-weakened dynamic frictional strength and homogeneous depth-dependent initial stress:
  - Suppl. Fig S20: Illustrative diagram of fault-local frictional evolution for heterogeneous friction or prestress setup.
  - Suppl. Fig S21: Depth-dependent variation of frictional strength and initial stress conditions along the hypocentral dip profile.
  - Suppl. Fig. S22: Model setup and overview.
  - Suppl. Fig. S23: Slip-rate evolution of the dynamic rupture model with heterogeneous distribution of fully- weakened dynamic frictional strength and homogeneous, depth-dependent initial stress.
  - Suppl. Video S5: Video of slip rate, along-dip shear stress, and friction.
- Dynamic rupture model using the stress-change pattern derived from the finite-fault model of Kubota et al. (2022):
  - Suppl. Fig. S24: Model setup and overview.
  - Suppl. Fig. S25:Slip-rate evolution of the dynamic rupture model using stress-change pattern derived from the finite-fault slip model of Kubota et al., 2020.
  - Suppl. Video S6: Video of slip rate, along-dip shear stress, and friction.
- Dynamic rupture model using the stress-change pattern derived from the finite-fault model of Melgar et al. (2015):
  - Suppl. Fig. S26: Model setup and overview.

- Suppl. Fig. S27: Slip-rate evolution of the dynamic rupture model using stress-change pattern derived from the finite-fault slip model of Melgar et al. (2015).
- Suppl. Video S7: Video of slip rate, along-dip shear stress, and friction.

• Dynamic rupture model using the stress-change pattern derived from the finite-fault model of Yamazaki et al. (2018):

- Suppl. Fig. S28: Model setup and overview.
- Suppl. Fig. S29: Slip-rate evolution of the dynamic rupture model using stress-change pattern derived from the finite-fault slip model of Yamazaki et al. (2018).
- Suppl. Video S8: Video of slip rate, along-dip shear stress, and friction.

Table S1: Fault frictional properties assumed in this study. VW: velocity-weakening, VS: velocity-strengthening.

Parameter	Symbol	Values	Unit
Direct-effect parameter*	a	VW: 0.01 (0–9 km, > 45 km)<br>VS: 0.018 (9–45 km)	
Evolution-effect parameter	b	0.014	
Reference slip rate	V_0	10^{-6}	m/s
Steady-state low-velocity friction coefficient at slip rate V_0	f_0	0.5	
Weakened slip rate	V_W	0.1	m/s
State evolution distance †	L	0.6	m
Fully weakened friction coefficient	f_w	0.1	
Initial slip velocity	V_i	10^{-16}	m/s

* The a parameter smoothly transitions from a velocity-strengthening (VS) value at depths shallower than 9 km and deeper than 45 km to a velocity-weakening (VW) value within the seismogenic zone (9–45 km).
† State evolution distance L is initially set to 0.2 m within 6 km radius of the hypocenter location, increases linearly to 0.6 m within a 12 km radius, and remains constant of 0.6 m elsewhere.
(See Supplementary Section "SM2: Nucleation" for details)

SM1: Model resolution

Numerical convergence of dynamic rupture simulations is governed by the resolution of the process zone [101]. We follow Wollherr *et al.* [124] to determine the required on-fault resolution of our SeisSol dynamic rupture simulations, which use basis functions of polynomial order $p = 5$. Our mesh features an element size of 1000 m everywhere along the slab. This ensures that we resolve the average process zone width, which we measure to be Λ=4,500 m in our preferred model.

Off-the slab, we employ a velocity-aware adaptive mesh refinement approach [96], focusing resolution along the slab interface and in onshore regions. The target frequency resolved by the mesh is determined by:

$$f \approx V_s/(\Delta x \times \text{elements per wavelength}), \tag{26}$$

with Δx defining the tetrahedral element size, V_s as the S wave speed. We follow the analysis by [154] and require at least two elements per wavelength, suitable for polynomial basis functions of order $p = 5$ in space and time. While our mesh is conservatively designed to resolve seismic wave propagation throughout the domain at frequencies up to 1 Hz, it resolves seismic wavefields recorded at seismic stations at frequencies up to 2 Hz (Supplementary Fig. S10).

SM2: Nucleation

The Tohoku-Oki earthquake began with a low initial moment-release rate [45, 48, 155], which is challenging to capture in dynamic rupture simulations that cannot account for long-term fault slip evolution. Our models capture the slow initiation behavior using a smooth nucleation procedure and scale-dependent fracture energy in the hypocentral region [149, 156, 157].

Following common practice from community dynamic rupture benchmarks [94], we define an overstressed nucleation region with a radius of r_{nuc} of 7 km and an additional shear stress perturbation of 10 MPa to locally reach the yielding stress level. We position the nucleation patch at the hypocenter location provided by the USGS (142.7897°E, 38.0919°N) [48]. The stress perturbation is smoothly imposed spatially and temporally, using an exponential spatial function $f(r)$ and a smooth temporal function $g(t)$:

$$f(r) = \exp[r^2/(r^2 - r_{crit}^2)]\,, \tag{27}$$

$$g(t) = \exp[(t-T)^2/t(t-2T)]\,, \tag{28}$$

with $T = 3s$.

To ensure a realistic, gradual rupture initiation, we impose a spatially variable slip-weakening distance [40]. The state evolution distance is set to $L = 0.2$ m within a 6 km radius from the hypocenter, increases linearly to 0.6 m within a 12 km radius, and remains constant at $L = 0.6$ m and beyond. To quantify the effects of varying slip-weakening distances, we perform an additional simulation using a uniform slip-weakening distance of 0.3 m. This uniform nucleation model reproduces the overall dynamic complexity seen in our preferred model, including multiple rupture reactivation, depth-dependent rupture characteristics, substantial slip to the trench, and

spontaneous rupture arrest (Supplementary Fig. S18). However, it results in the peak slip rate being reached early, at 50 s. This dynamic rupture model also does not match the geodetic deformation as closely as our preferred model, with an onshore and offshore geodetic data variance reduction of 76.1% and 34.1%, respectively.

SM3: Dynamic stress drop

To quantify the modeled spatially varying dynamic stress drop ($\Delta\tau$), defined as the difference between initial and final shear stresses during the rupture, we compute the slip-weighted mean stress drop across the ruptured area:

$$\Delta\sigma_E = \frac{\int_\Sigma \Delta\tau\delta \mathrm{dS}}{\int_\Sigma \delta \mathrm{dS}}\,, \tag{29}$$

where Σ is the rupture area and δ denotes the slip amplitude. Our preferred rupture model yields a slip-weighted average dynamic stress drop of 2.37 MPa, comparable to the estimated stress drop of finite-fault slip models [47].

Table S2: 1D velocity model, modified from [100]

Depth[km]	Thickness [km]	P-wave velocity [km/s]	S-wave velocity [km/s]	Density [kg/m^3]
3	3	5.5	3.14	2300
18	15	6.0	3.55	2400
33	15	6.7	3.84	2800
100	67	7.8	4.46	3200
∞	∞	8.0	4.57	3300

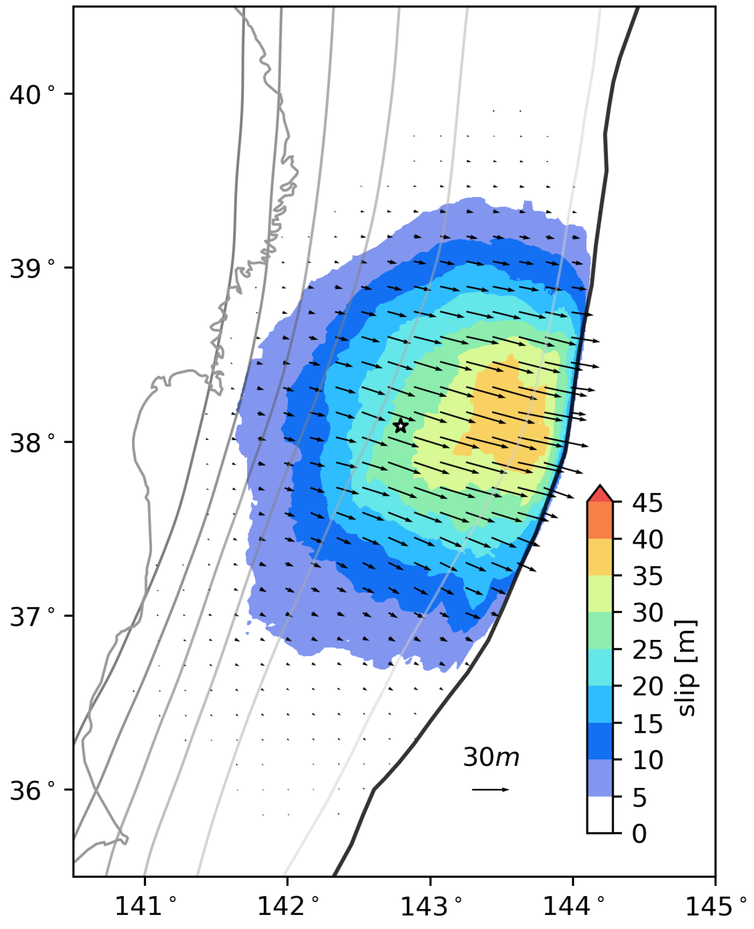

Figure S1: **Median slip distribution computed from 32 finite-fault slip distributions of the Tohoku-Oki earthquake compiled by [10] and projected onto our new slab geometry** (Methods Sec.'Model geometry and mesh'). Colors and vectors represent the amplitude and direction of slip. Gray contour lines indicate the slab geometry at 10 km depth intervals. The USGS hypocenter is indicated as the star [48]. The median slip model reveals a smoothly distributed circular slip patch predominantly updip from the hypocenter, confined mostly along strike. Large slip extends toward the trench, reaching a maximum amplitude of approximately 38.0 m roughly 5 km away from the trench axis. This major slip feature has been recognized in [9, 158, 159], although previous discussions have been largely qualitative.

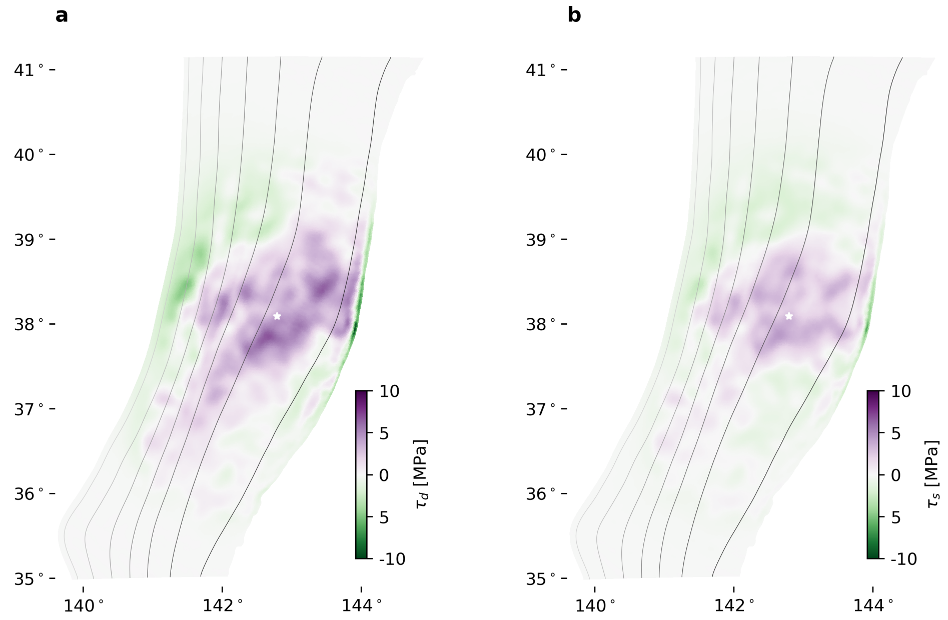

Figure S2: **Stress changes resulting from the median finite-fault slip distribution on the megathrust interface.** (a) Along-dip shear stress change. (b) Along-strike shear stress change. These stress changes serve as the basis for constructing the observationally informed initial stress conditions for dynamic rupture modeling.

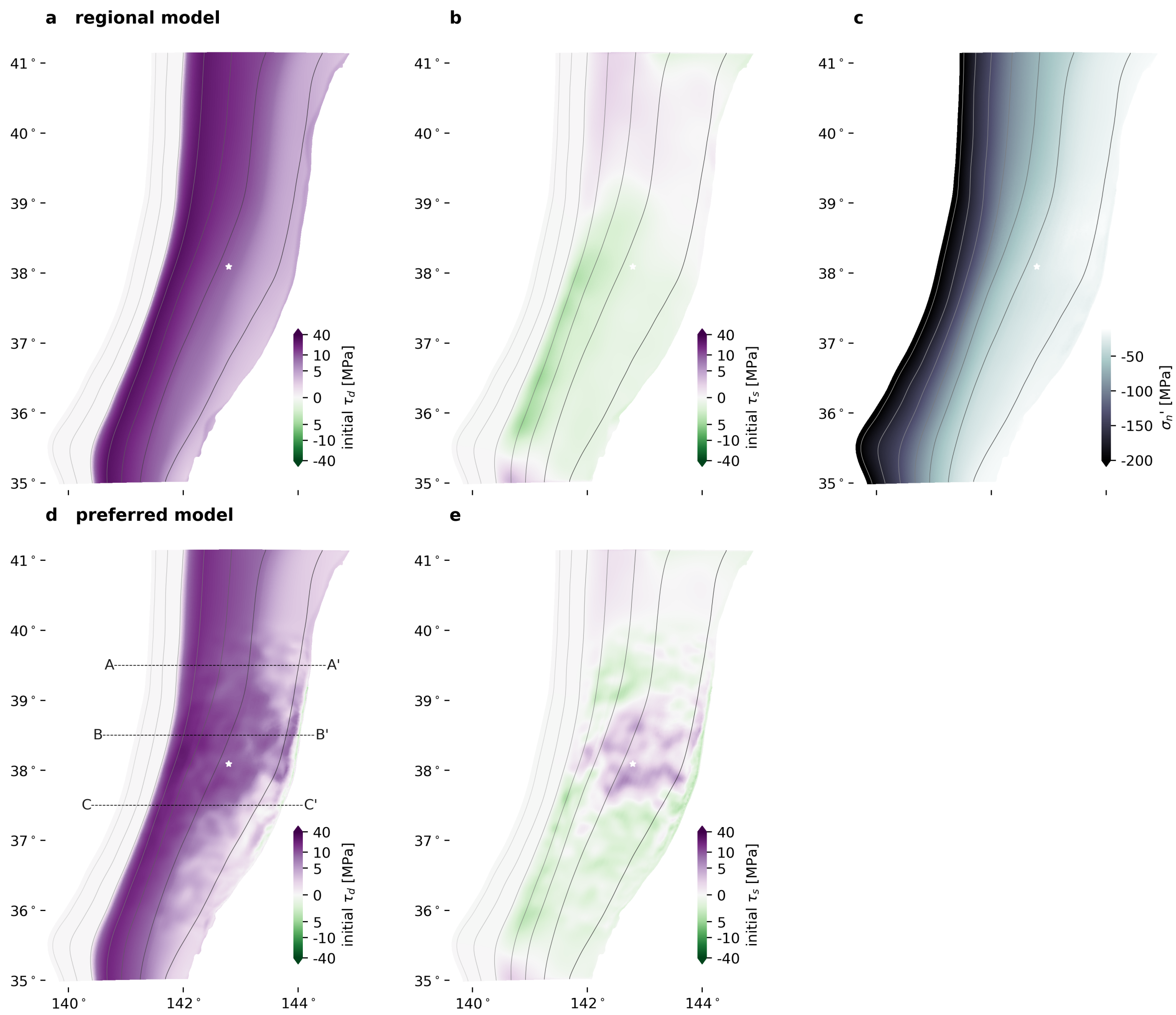

Figure S3: **Initial shear stress and effective normal stress distributions along the megathrust interface.** (a, b) Initial shear stress (τ_d, τ_s) distribution for the homogeneous regional stress dynamic rupture model (shown in Fig. 1b). (c) Depth-dependent distribution of effective normal stress (σ_n'). (d, e) Initial shear stress distribution for the preferred model incorporating stress heterogeneity from the median finite-fault model in Wong *et al.* [10]. Hypocenter location (star) and depth contours (gray lines, 10 km intervals) are shown in all panels.

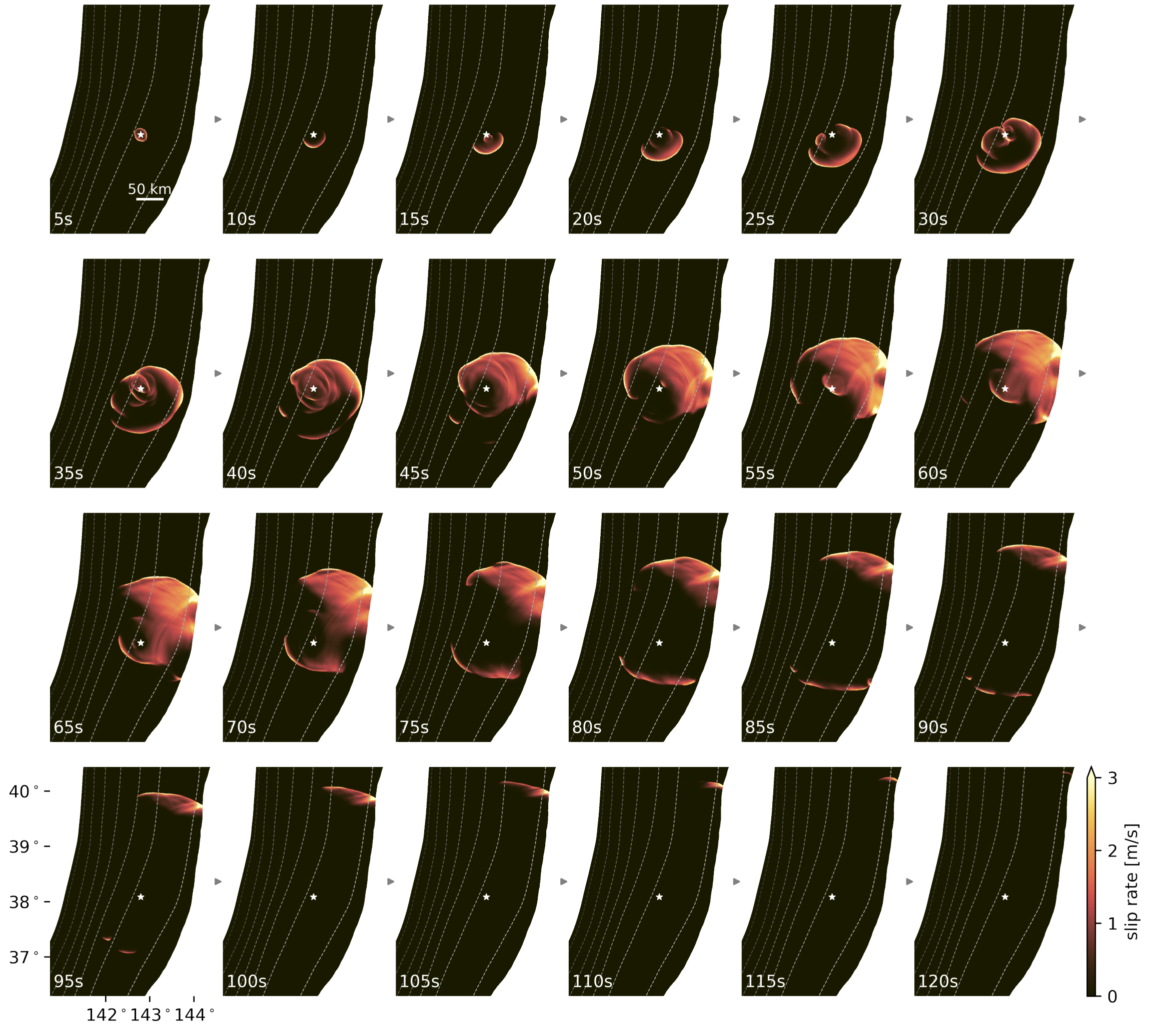

Figure S4: **Dynamic rupture evolution of the preferred model.** Snapshots of slip rate shown in 5 s intervals, see also Supplementary Video S1. Earthquake rupture initiates as a growing pulse within the first 15 s, followed by a first rupture reactivation initiating at the primary pulse' healing front between 15–25 s. Between 25–40 s, reactivated rupture fronts coalesce, "spiral" and back-propagate, resulting in complex slip rate patterns and a second major hypocentral slip reactivation at 40 s, taking again the form of a growing pulse. Between 40 s and 50 s rupture time, the primary updip rupture front reaches the seafloor interface, resulting in strong dynamic interactions with the free surface and generating reflected phases. The third episode of hypocentral rupture reactivation occurs at around 50 s, initiating at the healing front of the secondary propagating pulse-like rupture. A fourth reactivated pulse emerging at 65 s is not sustained. Subsequently, after around 75 s, the rupture simplifies and propagates pulse-like bilaterally along strike, featuring extended shallow rupture in the northern portion of the megathrust between 100–120 s, consistent with slip models inferred from tsunami inversion studies [60, 69, 70]. The white star denotes the hypocenter location.

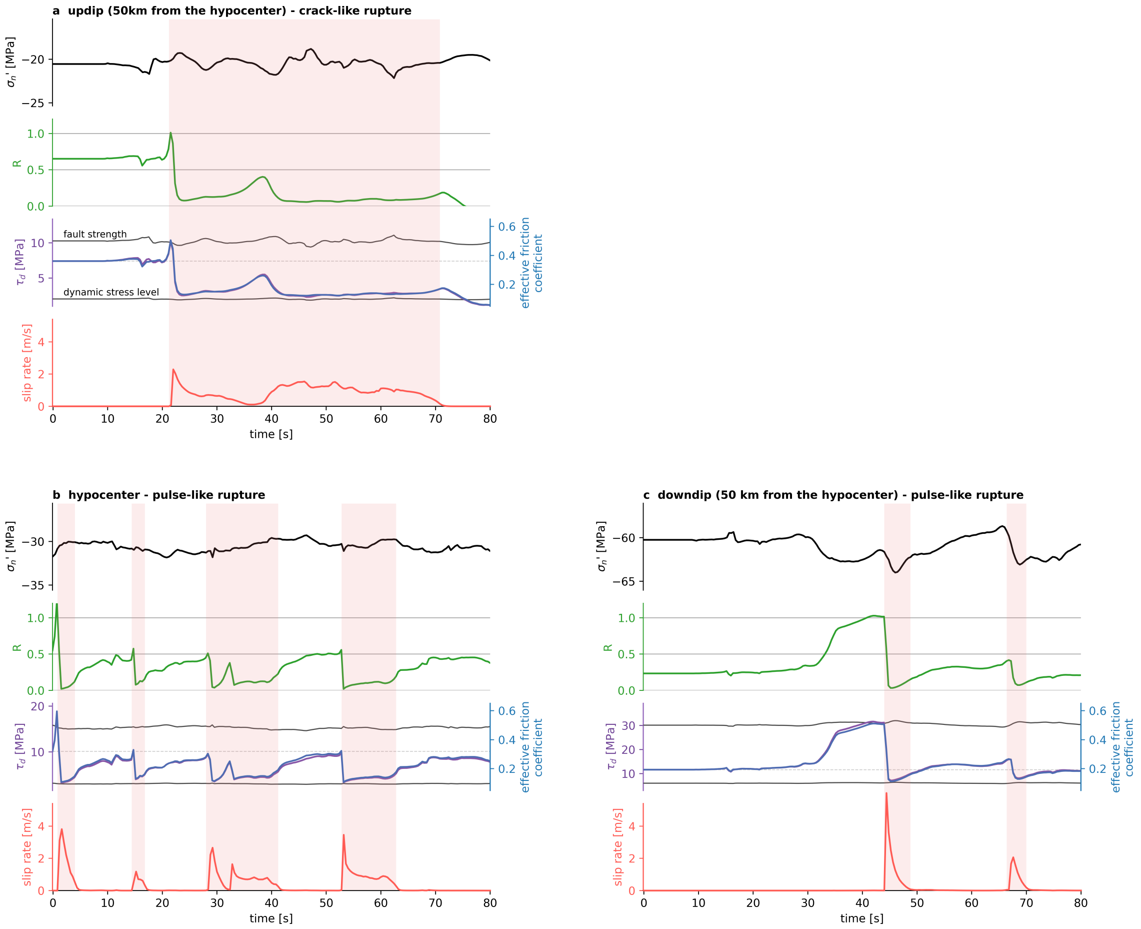

Figure S5: **Temporal evolution of effective normal stress σ_n' (black), relative prestress ratio R (green), along-dip shear stress τ_d (purple), effective friction coefficient (blue), and slip rate (red) of the preferred model.** (a) Evolution in the updip, (b) hypocentral, and (c) downdip regions. The shaded red areas denote periods when the slip rate exceeds 0.05 m/s. In the along-dip shear stress panels, the light gray dashed lines represent the initial shear stress, while the solid black lines indicate the fault strength ($f_0\sigma_n'$) and dynamic stress level ($f_w\sigma_n'$).

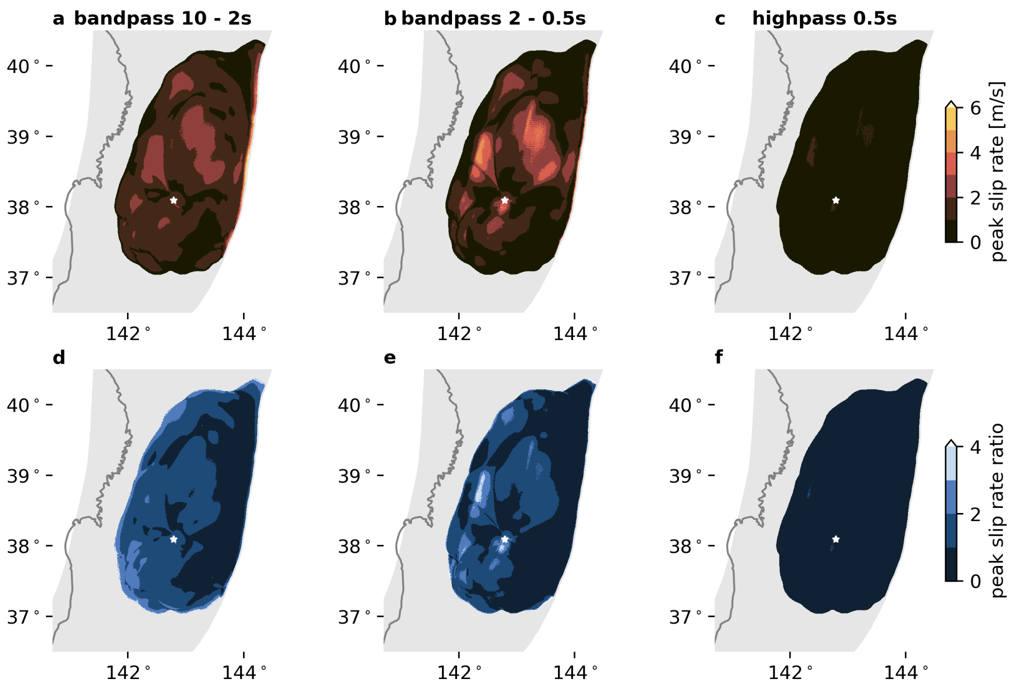

Figure S6: **Comparison of the peak-slip-rate distributions of the preferred model at three frequency ranges.** Top row: filtered peak slip-rate distribution with the same plotting style as Fig.4c. Bottom row: Ratio of peak-slip-rate distribution between the top row and the low-pass filtered at 10 s with the same plotting style as Fig. 4e. (a, d) Comparison with band-pass filtered between 10 and 2 s. (b,e) Band-pass filtered between 2 and 0.5 s. (c,f) High-pass filtered at 0.5 s. The downdip high-frequency radiation is mostly dominated in the back-propagation study seismic frequency range of 2 to 0.5 s.

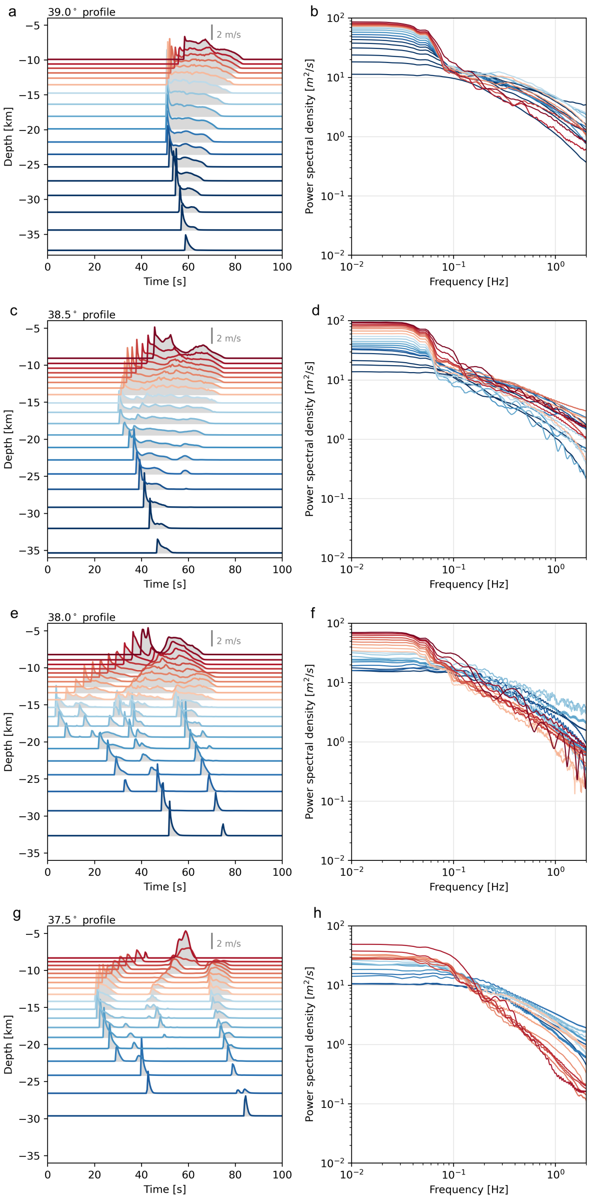

Figure S7: **Along-dip slip-rate time histories and corresponding power spectral densities for the preferred model.** Left column (a,c,e,g): slip-rate evolution with depth along four dip profiles. Right column (b,d,f,h): power spectral density of the slip-rate time series for each profile. Across all profiles, local slip duration (rise time) increases toward shallower depths; the contrast is most pronounced along the 38.0° profile.

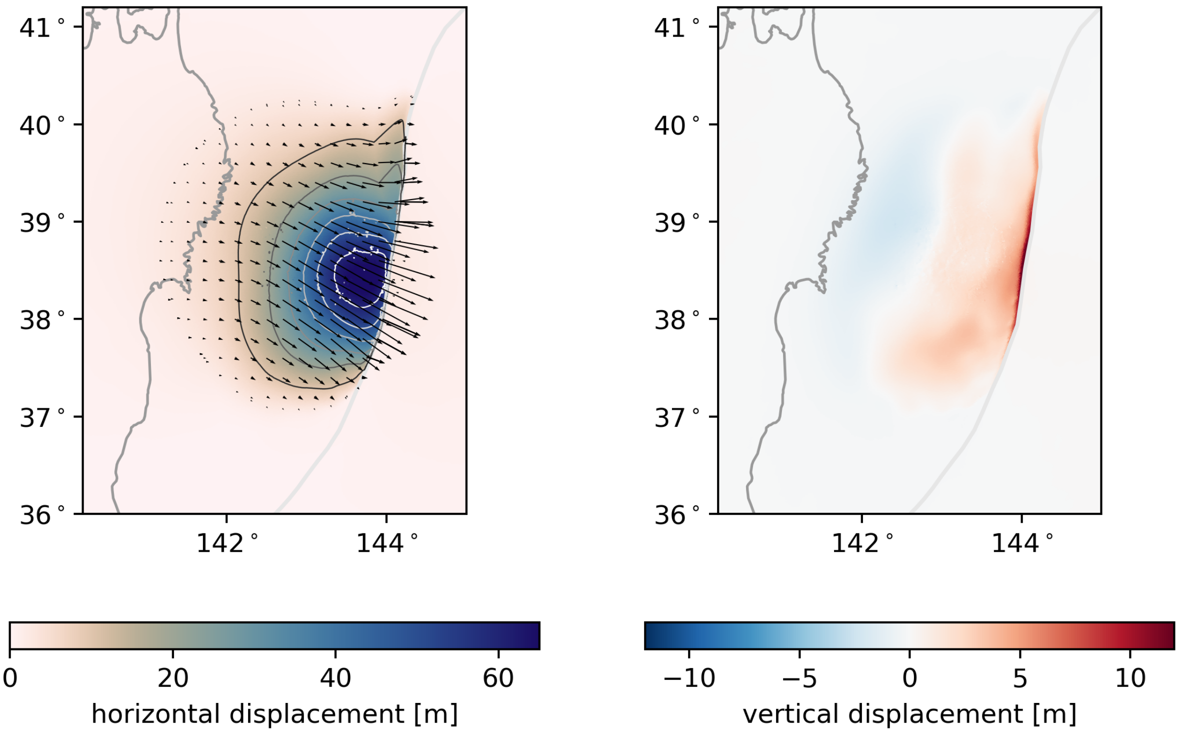

Figure S8: **Simulated seafloor displacement from the preferred model.** (a) Horizontal and (b) vertical displacement fields. In (a), contours show horizontal displacement amplitudes at 10 m intervals, the gray line denotes the trench location. The near-trench modeled horizontal and vertical displacements are broadly consistent with differential bathymetry observations [8, 56, 58, 160, 161]. The pronounced uplift in the northern near-trench region (in b) agrees with deformation inferred from tsunami waveform inversion [60, 69, 70, 162, 163].

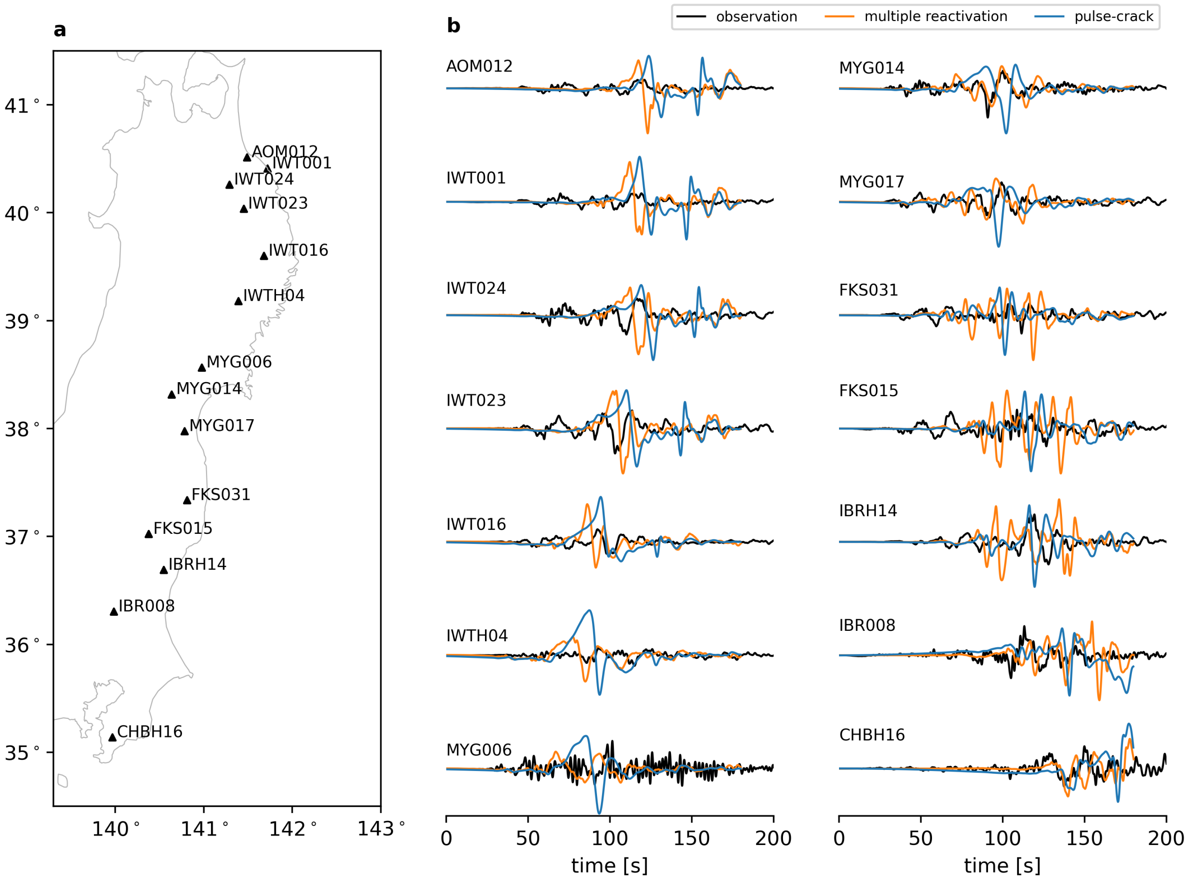

Figure S9: **Comparison of the observed regional strong-ground motion with synthetics from the preferred multiple-reactivation model (orange) and the simple reactivation model (blue)**. (a) Map of the KiK-net and K-NET strong-ground-motion stations used. (b) Waveform comparison between the observations (black) and synthetics from the preferred multiple-reactivation model (orange) and the simple pulse–crack model (blue). The vertical components are shown in velocity, bandpass-filtered between 100 and 1 s period. The synthetics from the preferred model with multiple reactivation exhibit multiple move-out branches, whereas the simple pulse–crack model synthetics display a single dominant phase. The preferred model synthetics match the waveforms at the MYG014 and MYG017 stations, located near the major rupture area.

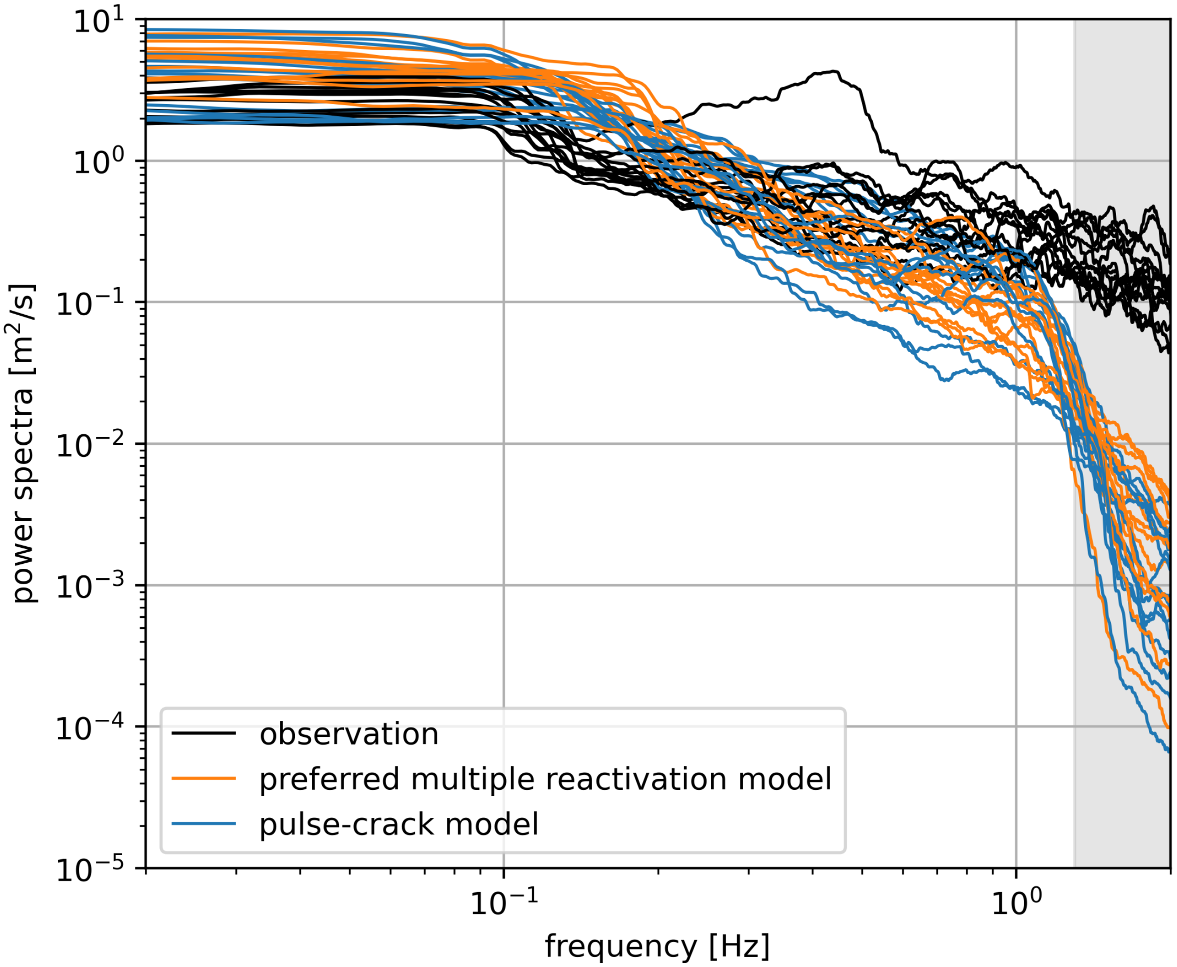

Figure S10: **Comparison of power spectra of modeled waveforms at onshore strong-motion K-net stations with observations (black), preferred model (orange), pulse-crack model (blue).** The shaded area shows the effective maximum frequency resolved in the simulated seismic wavefield at 1.5 Hz.

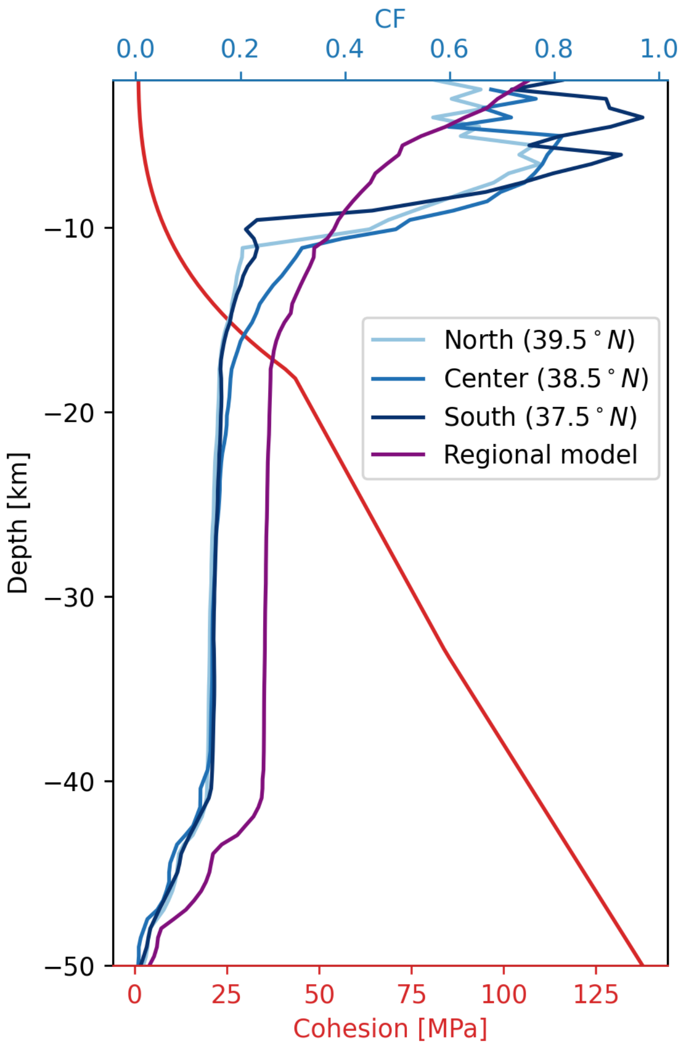

Figure S11: **Depth-dependent cohesion (red) and closeness-to-failure** (CF) **profiles** across the north (light blue), center (blue), and south (dark blue) cross sections as of Fig 6 of the preferred model, and the laterally homogeneous prestress model (purple). See Methods Sec. “Off-fault plasticity”.

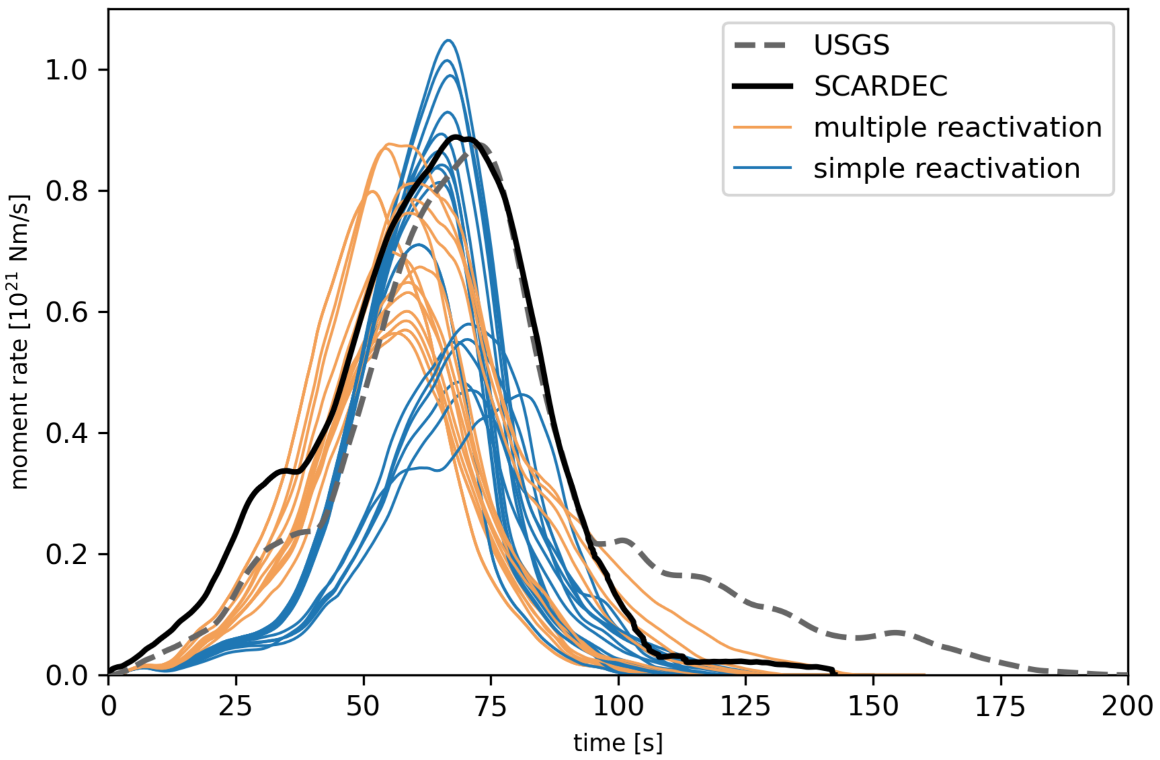

Figure S12: **Comparison of moment-rate functions for two distinct rupture styles shown in Figure 9.** Yellow lines represent the moment-rate functions of dynamic rupture models characterized by repeated rupture reactivation near the hypocenter, while blue lines correspond to models dominated by single pulse-like ruptures with free-surface reflection. The reactivation model captures the early moment-rate evolution within the first 40 s, while the simple reactivation rupture model underestimates moment release during the 0–40 s rupture time interval.

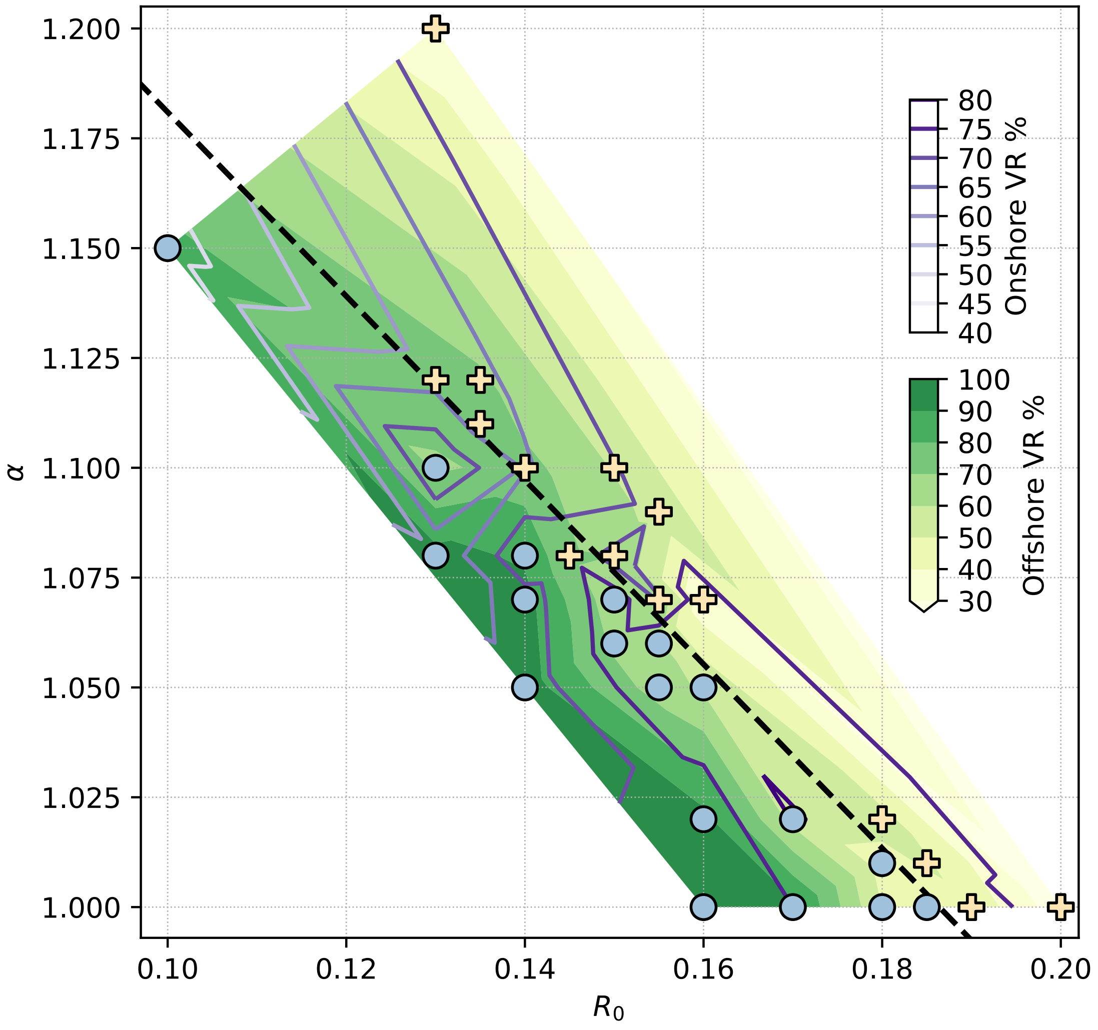

Figure S13: **Comparison of onshore and offshore geodetic displacement misfits across models with varying prestress heterogeneity amplitude** (α) **and regional relative prestress level** R_0**.** Green-filled contours indicate variance reduction for offshore geodetic data, while purple contour lines represent variance reduction for onshore data. Blue circles represent a family of dynamic rupture models dominated by single pulse-like ruptures driven by free-surface reflection, while yellow crosses denote models exhibiting repeated rupture reactivation near the hypocenter. Our results illustrate that stress heterogeneity amplitude (α) primarily controls peak slip magnitude, whereas the regional relative stress level R_0 predominantly determines rupture extent.

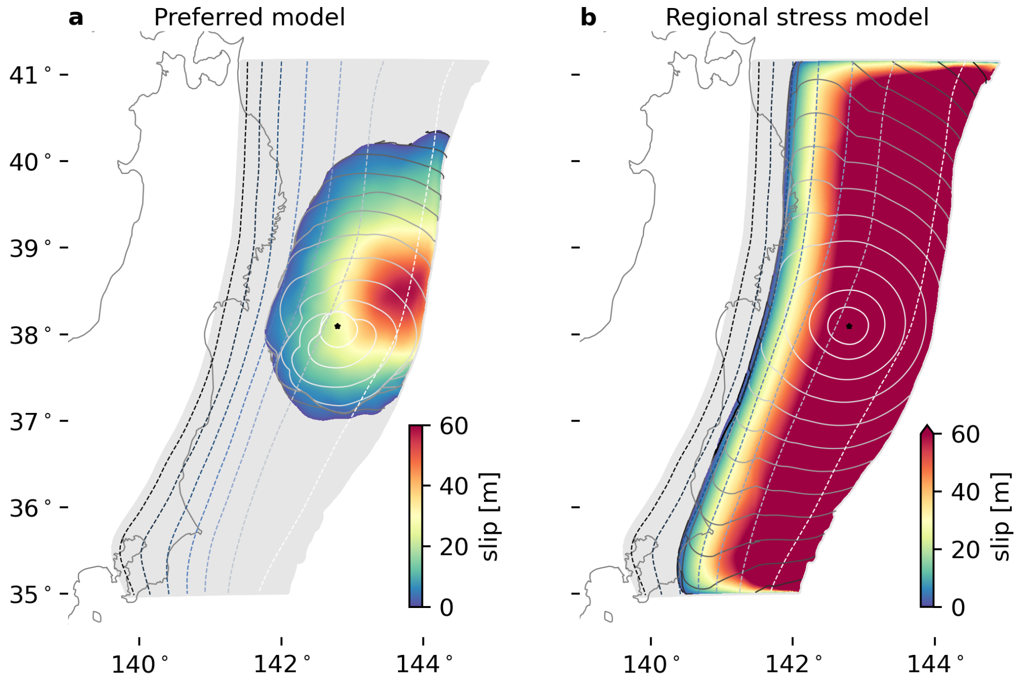

Figure S14: **Comparison of fault slip distribution and rupture evolution between (a) the heterogeneous prestress model (the preferred model) and (b) the laterally homogeneous prestress model.** Solid contour lines indicate rupture front location at 10 s intervals. The preferred model spontaneously arrests with M_w=8.96, whereas the laterally homogeneous prestress model fails to arrest and ruptures the entire fault, reaching M_w=9.61. Depth contours (dotted lines, 10 km intervals) and the hypocenter location (star, [48]) are shown in both panels.

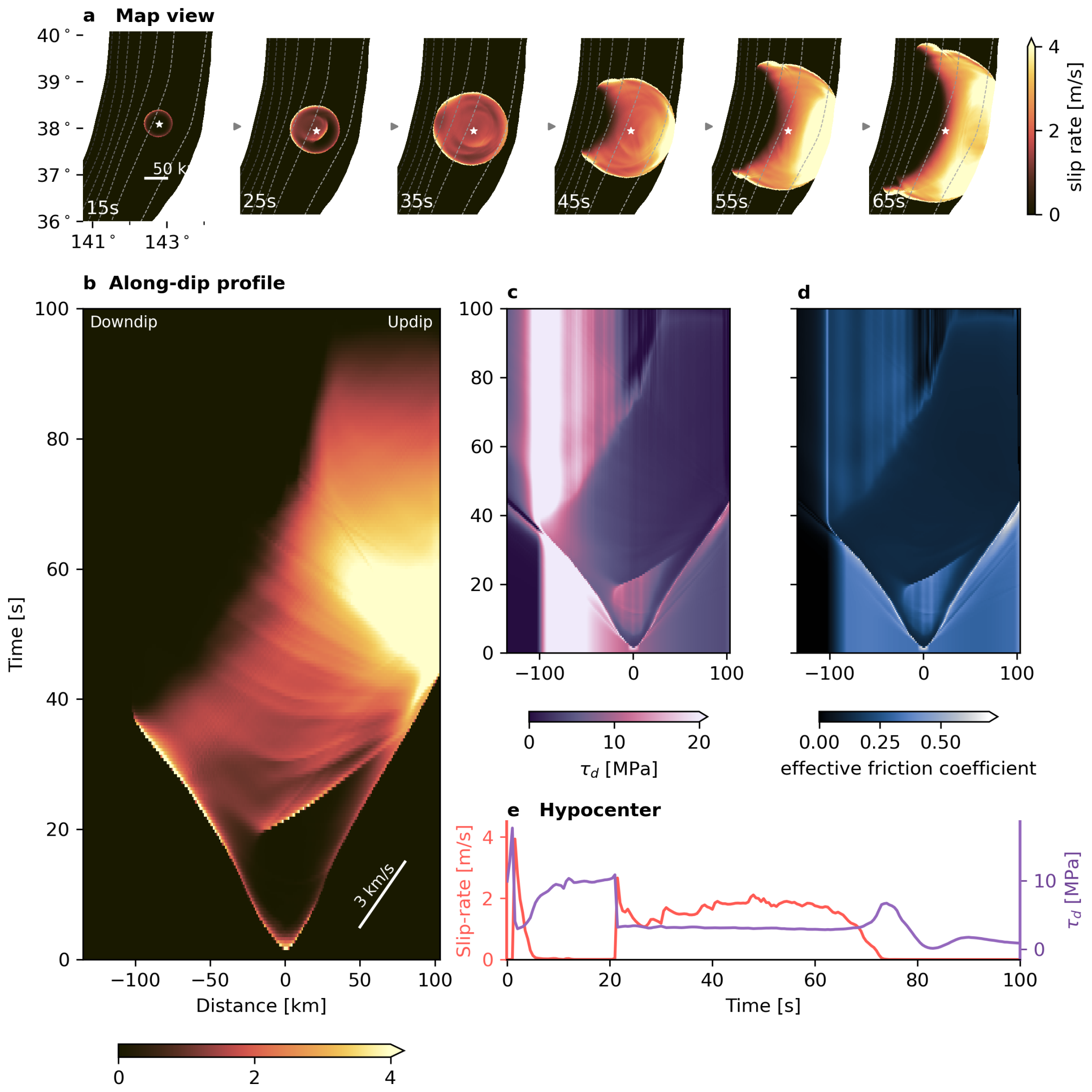

Figure S15: **Rupture dynamics of the laterally homogeneous prestress model**, see also Supplementary Fig. S17 and Supplementary Video S2. (a) Map-view snapshots of slip rate evolution at 10 s intervals. A primary growing pulse is followed by crack-like slip reactivation at approximately 20 s, propagating updip. Primary and secondary rupture fronts subsequently merge into sustained crack-like rupture without clear healing fronts distinguishing separate slip episodes. The white star indicates the hypocenter location. (b)-(d) Temporal evolution of slip rate, along-dip shear stress τ_d (purple), and effective friction coefficient (blue) along a hypocentral dip profile, highlighting rapid coseismic restrengthening and subsequent rupture reactivation. (e) Time series of hypocentral slip rate (red) and along-dip shear stress τ_d (purple).

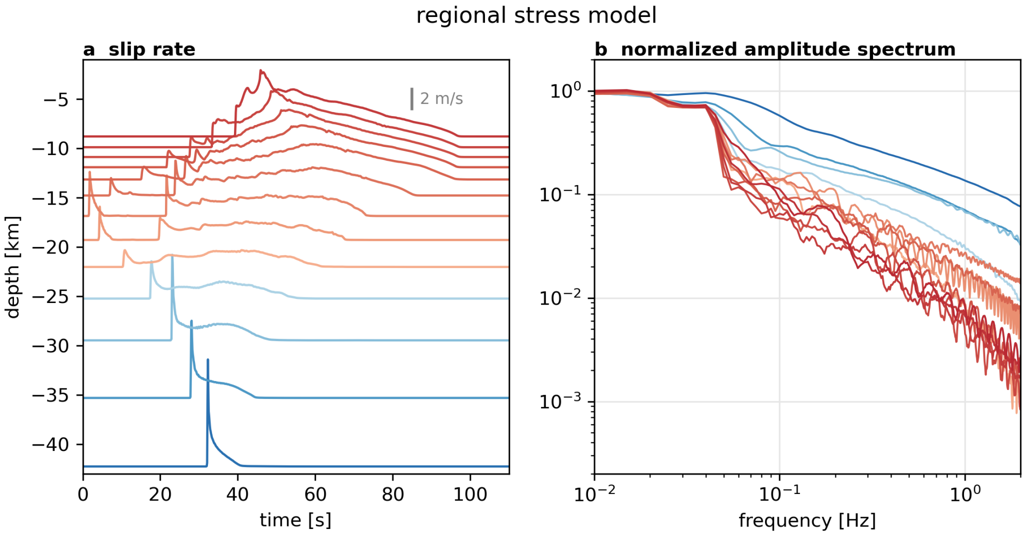

Figure S16: **Depth-dependent slip rate characteristics in the laterally homogeneous prestress model.** (a) Slip rate functions along-dip through the hypocenter at various depths. Downdip pulse-like ruptures are highlighted in blue, while updip crack-like ruptures are indicated in red. (b) Normalized amplitude spectra of the corresponding slip rate functions shown in (a), illustrating distinct frequency content between downdip pulse-like and updip crack-like rupture styles.

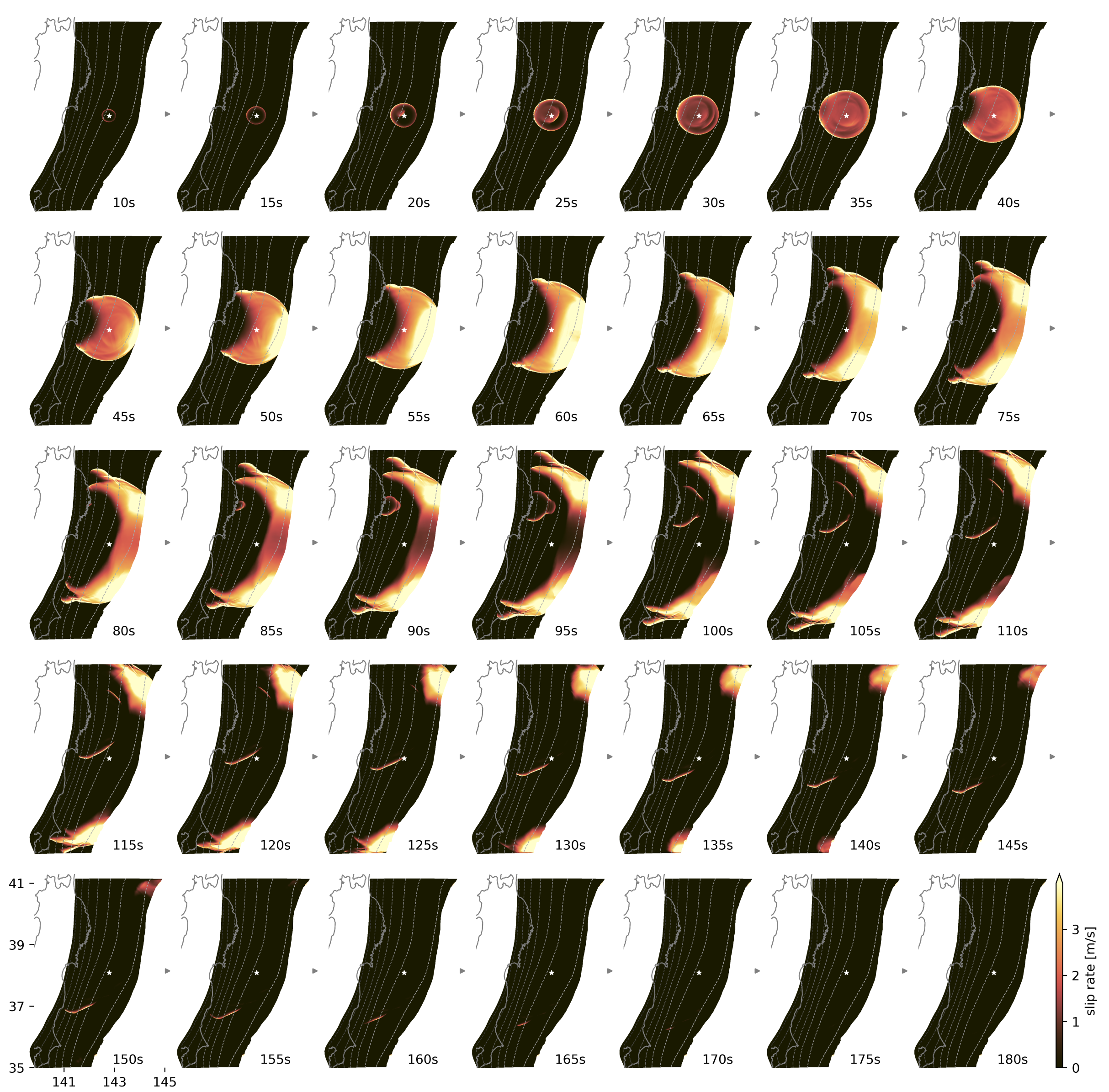

Figure S17: **Dynamic rupture evolution of the laterally homogeneous prestress model** (see also Fig. S15 and Supplementary Video S2). Snapshots of slip rate are shown in 5 s intervals. Earthquake rupture initiates as a growing pulse, followed by rupture reactivation initiating at the downdip healing front of the primary growing pulse at 20 s. Between 20–40 s, primary and secondary rupture fronts subsequently merge into a sustained, crack-like rupture without clear healing fronts separating slip episodes. At 40 s, rupture reaches the downdip limit of the seismogenic zone, forming a healing front that propagates updip and progressively shortens central slip rise times toward shallower depths as the rupture expands along strike. At the same time, bilateral deep supershear rupture is initiated ahead of the primary rupture front via the "daughter crack" mechanism [164] and likely due to higher effective normal stress and a relatively sharp transition to velocity-strengthening friction at depth [165]. This local supershear rupture remains confined to depths between 30–50 km. At 45 s rupture time, about 5 s later compared to the preferred model, the primary updip rupture front reaches the seafloor interface, resulting in reflected phases. Between 70–80 s, two secondary sub-Rayleigh rupture fronts re-rupture the down-dip part of the slab, including spiraling rupture dynamics and initiating backward-propagating fronts at 76 s (north) and 79 s (south). The northern reactivated front expands, triggering a local up-dip supershear rupture at about 90 s and re-rupturing the central slab, while the southern front decays. Updip ruptures arrest at the slab boundary around 140 s, whereas the downdip rupture front continues propagating until approximately 180 s simulation time.

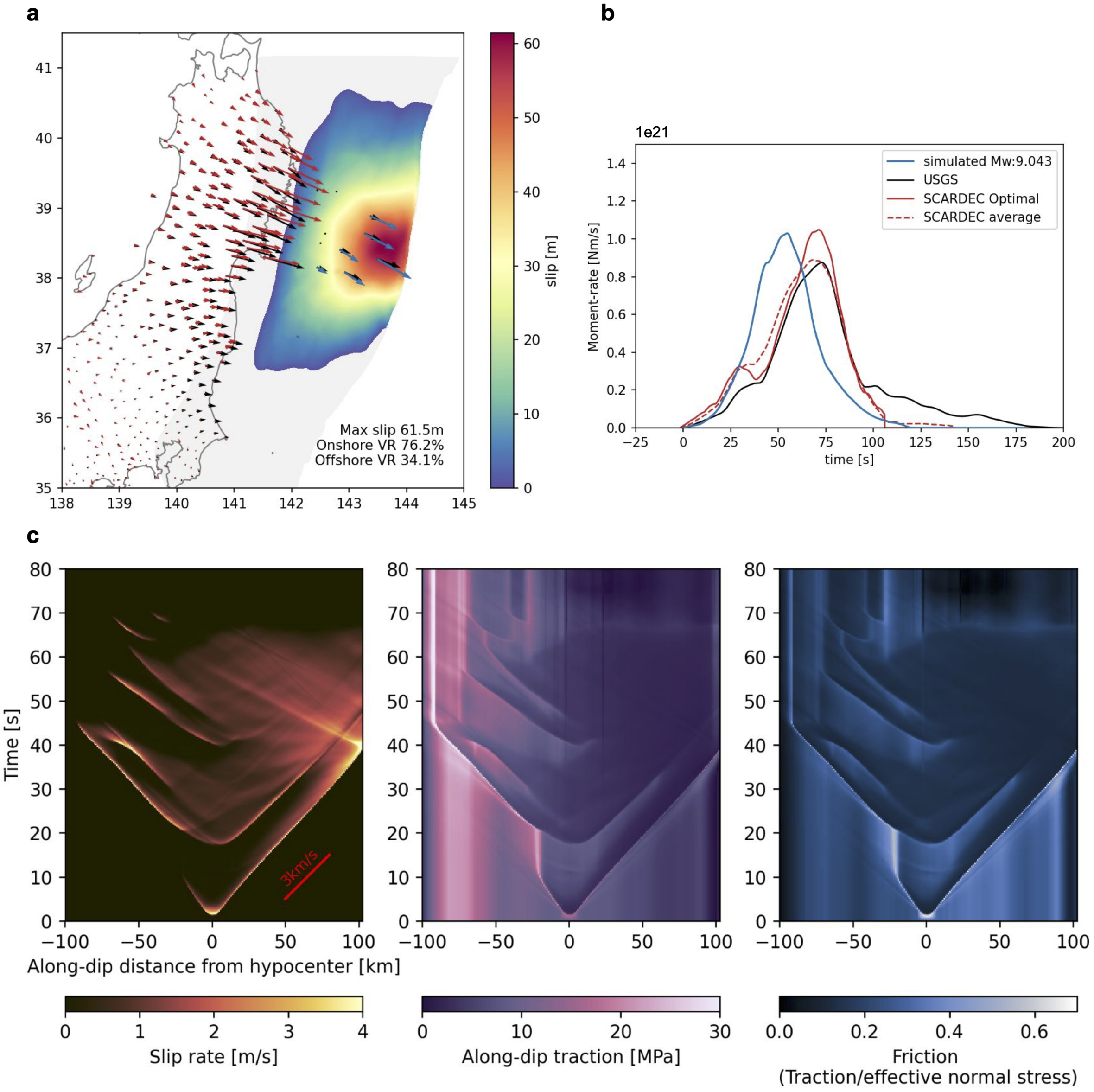

Figure S18: **Alternative dynamic rupture model using a uniform weakening distance L of 0.3 m** (See Supplementary section - SM2: Nucleation for details). (a) Fault slip distribution with geodetic data fit. Observed geodetic displacements are shown as black arrows. Onshore and offshore modeled displacements are shown as red and blue arrows, respectively. The model achieves a variance reduction of 76.2 % (onshore) and 34.1 % (offshore). (b) Comparison of modeled moment-rate function and moment-rate estimates from USGS [48] and SCARDEC [45]. (c) Temporal evolution of slip rate (red), along-dip shear stress (purple), and effective friction coefficient (blue) along the hypocentral dip profile, highlighting rapid variations coincident with dynamic rupture reactivation. This model features downdip pulse-like rupture and updip crack-like rupture characteristics.

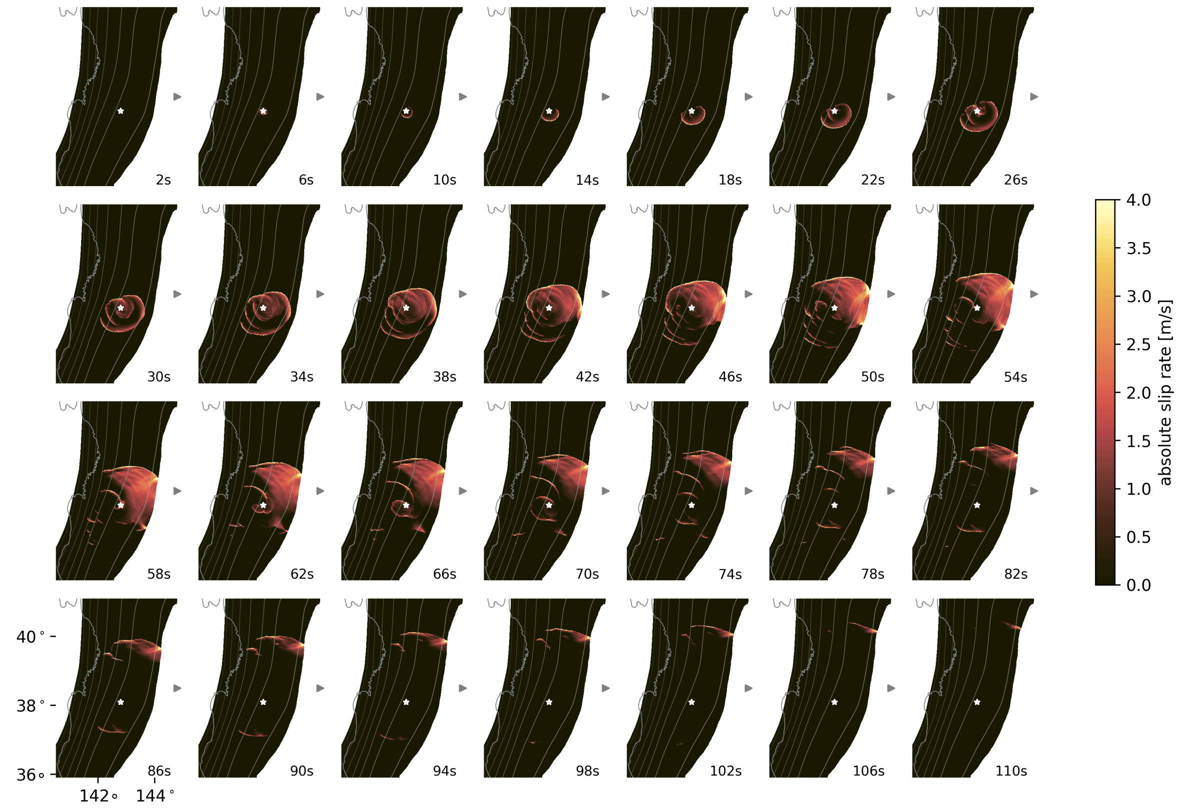

Figure S19: **Slip-rate evolution of the heterogeneous-friction dynamic rupture model with multiscale variations in state-evolution distance.** Snapshots are shown at 4 s intervals from 2 s to 110 s (left to right, top to bottom). Colors indicate absolute slip rate. Contours outline the slab depth at 10 km intervals. The simulation reproduces repeated slip reactivation and mixed downdip pulse-like and updip crack-like rupture styles. This simulation exhibits more frequent re-nucleation than the six episodes in the preferred model. See also Figure 10 and Video S4.

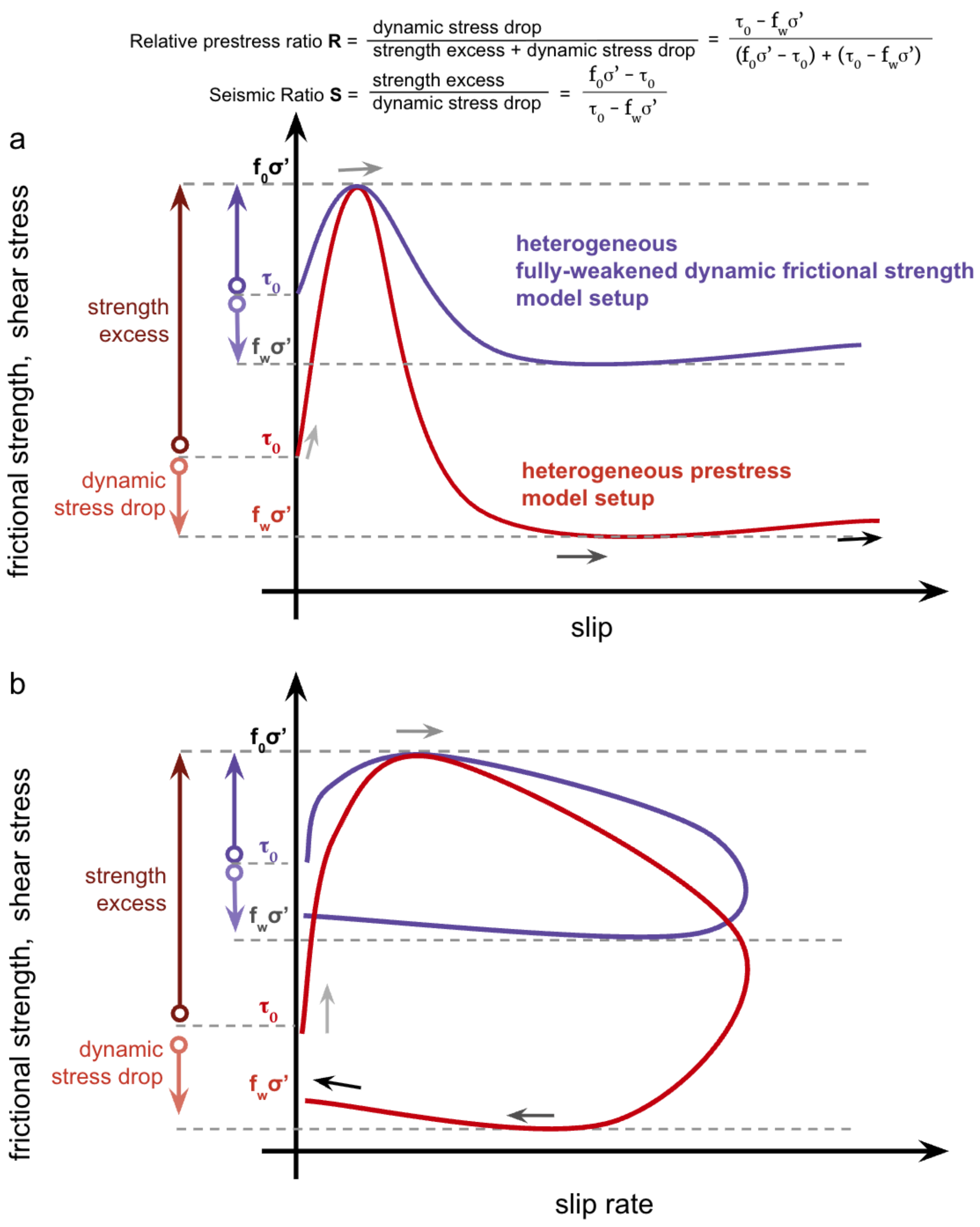

Figure S20: **Modeled fault-local frictional evolution in two alternative model setups with heterogeneity either in friction or in prestress.** a) friction evolution with slip. b) frictional evolution with slip rate. The purple curve shows the frictional strength evolution for the setup with a heterogeneous fully-weakened dynamic frictional strength. The red curve shows the frictional strength evolution for the preferred model with heterogeneous prestress. The grey arrows in both panels show the stages of frictional evolution correspondingly. The darker arrows in the y-axis labels indicate the frictional strength excess, and the lighter arrows indicate the dynamic stress drop. We added the definitions of the seismic ratio S and relative prestress level R. Given the same dynamic stress drop value, the heterogeneous fully-weakened dynamic frictional strength model has a lower ratio of strength excess to dynamic stress drop (S ratio, equation 16) than the heterogeneous prestress model.

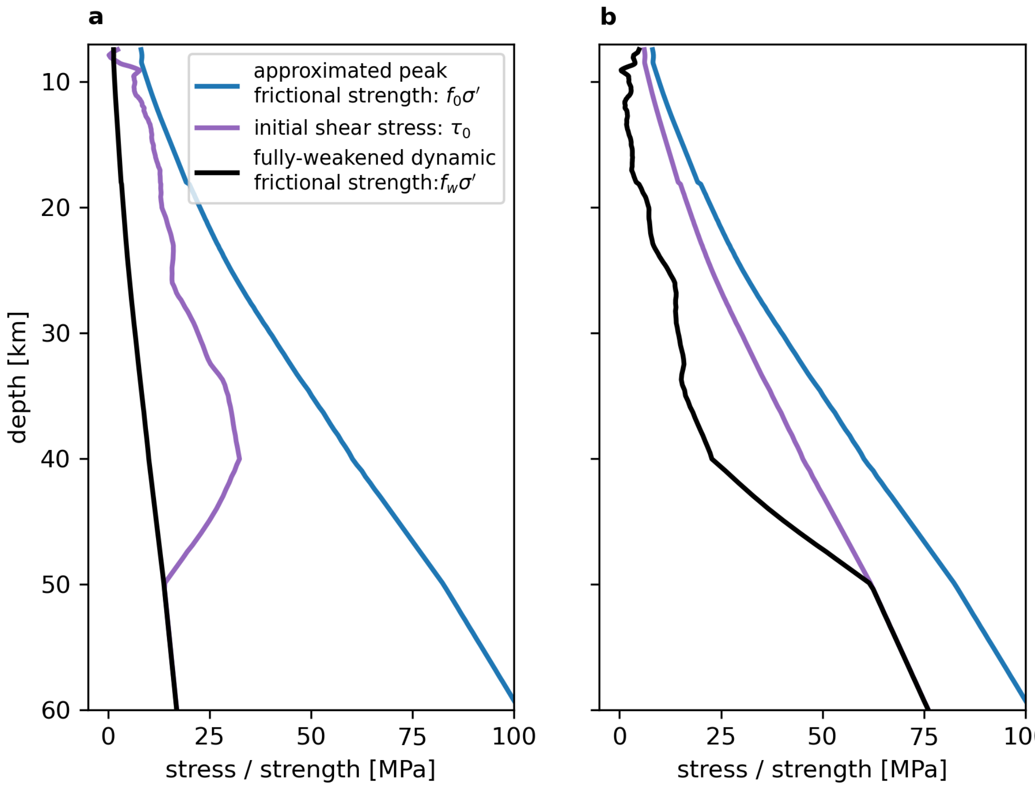

Figure S21: **Depth-dependent variation of frictional strength and initial stress conditions along a hypocentral dip profile using two alternative model setups with heterogeneity in dynamic parameters.** Blue, purple, and black lines represent the approximated peak frictional strength ($f_0\sigma'$), initial shear stress (τ_0), and fully-weakened dynamic frictional strength ($f_w\sigma'$), respectively (Methods section: **??**). (a) Heterogeneity in pretress. This model setup assumes depth-dependent frictional strength. The initial stress level follows the stress-change pattern inferred from the median slip distribution. (b) Heterogeneity in the fully-weakened dynamic frictional strength. This setup maps the heterogeneity onto the fully-weakened dynamic frictional strength while using a homogeneous depth-dependent initial stress.

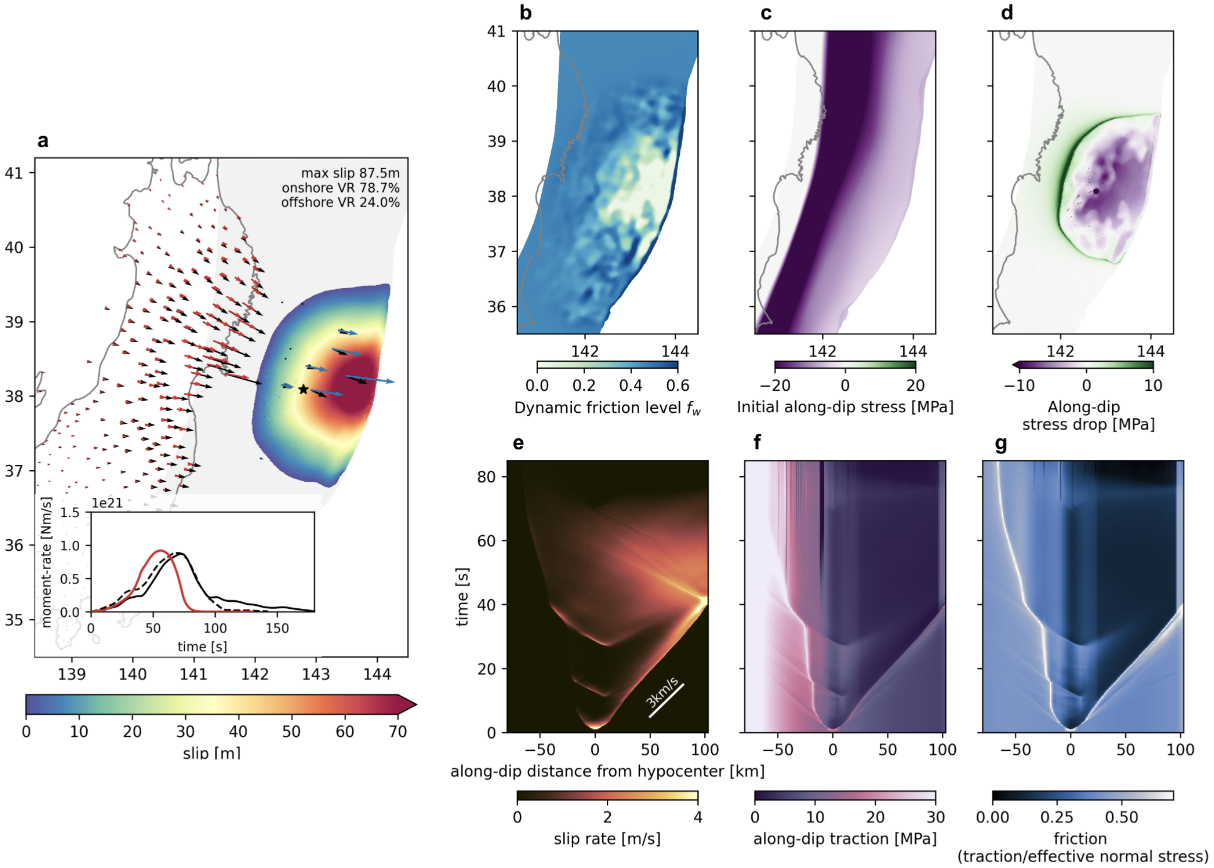

Figure S22: **Dynamic rupture scenario with heterogeneous distribution of fully-weakened dynamic frictional strength and homogeneous, depth-dependent initial stress.** (a) Simulated slip distribution and corresponding geodetic deformation. Red and blue arrows indicate synthetic onshore and offshore displacements, respectively. Black arrows show the observations. The inset compares the simulated moment-rate function (red), with the USGS (solid black), and SCARDEC (dashed black) source model moment-rate functions ([45, 48]). (b) Spatial distribution of the fully-weakened friction coefficient (f_w). (c) Distribution of initial along-dip shear stress. (d) Spatial distribution of the along-dip stress drop. (e-g) Along-dip profiles of slip-rate, along-dip traction, and effective friction coefficient evolution, from left to right, respectively.See also slip-rate evolution in Supplementary Figure S23 and Video S5.

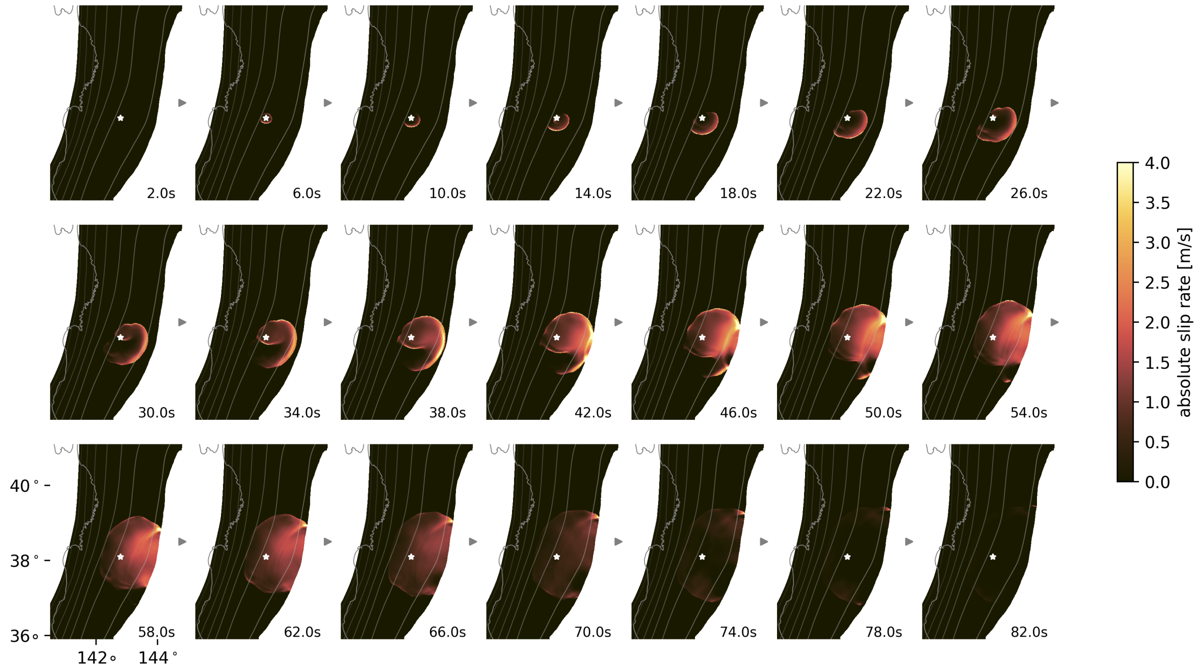

Figure S23: **Slip-rate evolution of the dynamic rupture model with heterogeneous distribution of fully-weakened dynamic frictional strength and homogeneous, depth-dependent initial stress**. Snapshots are shown at 4 s intervals from 2 s to 82 s (left to right, top to bottom). Colors indicate absolute slip rate. Contours outline the slab depth at 10 km intervals. The simulation reproduces slip reactivation, mixed pulse-like and crack-like rupture styles, and large slip to the trench. Compared to the preferred heterogeneous prestress model, the depth-dependent variability of rupture style in this simulation is less clear, and rupture transitions into a pure crack-like style after 50 s simulation time. See also Supplementary Figure S22 and Video S5.

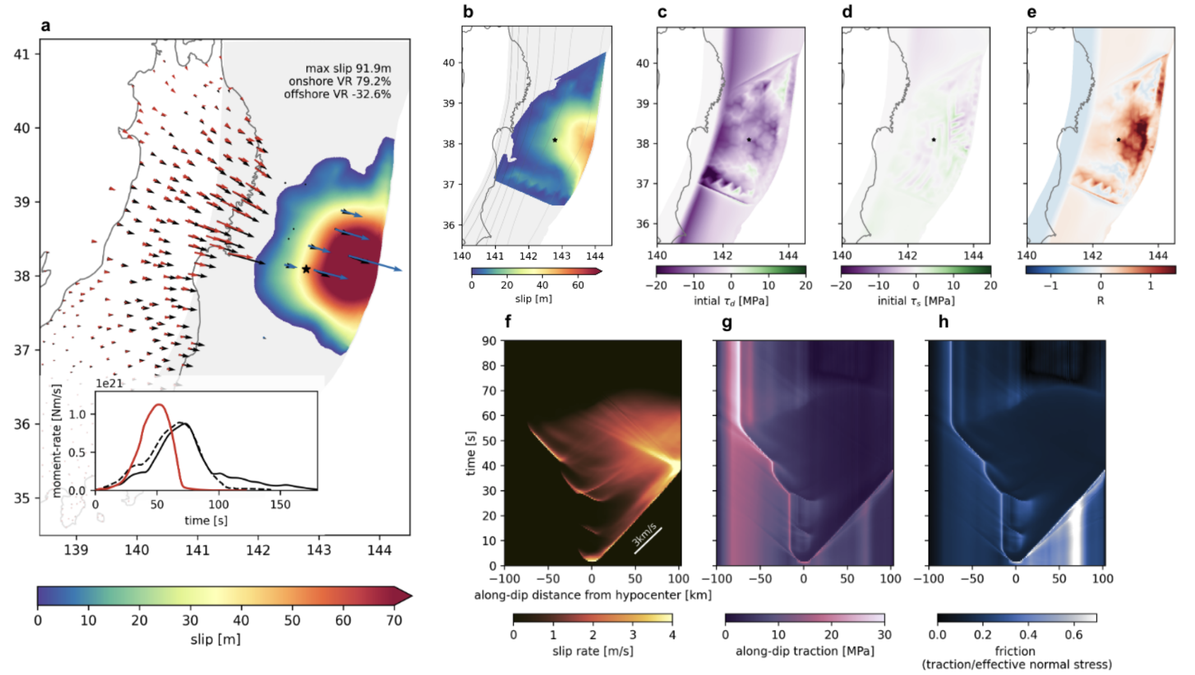

Figure S24: **Dynamic rupture model using the stress-change pattern derived from the finite-fault slip model of Kubota et al. (2022).** (a) Simulated slip distribution and corresponding geodetic deformation. Red and blue arrows indicate synthetic onshore and offshore displacements, respectively. Black arrows show the observations. The inset compares the simulated moment-rate function (red), with the USGS (solid black), and SCARDEC (dashed black) source model moment-rate functions ([45, 48]). (b) Kubota et al., 2022 finite-fault model slip distribution. (c-e) Distribution of initial along-dip shear stress (τ_d), along-strike shear stress (τ_s), and relative prestress ratio (R), from left to right, respectively. (f-h) Along dip profile of slip rate, along-dip traction, and effective friction coefficient, from left to right, respectively. See also Supplementary Figure S25 and Video S6.

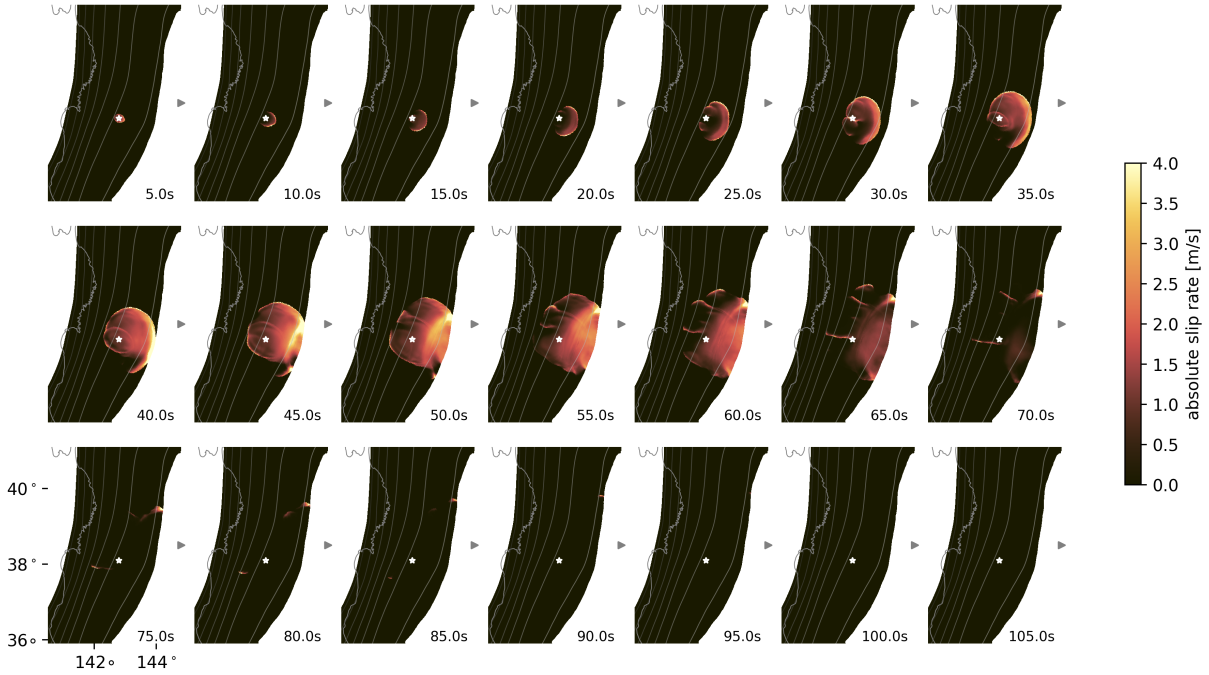

Figure S25: **Slip-rate evolution of dynamic rupture model using stress-change pattern derived from the finite-fault slip model of Kubota et al. (2022).** Snapshots are shown at 5 s intervals from 5 s to 105 s (left to right, top to bottom). Colors indicate absolute slip rate. Contours outline the depth at 10 km intervals. The simulation reproduces repeated slip reactivation and mixed downdip pulse-like and updip crack-like rupture styles. See also Supplementary Figure S11 and Video S4. See also Supplementary Video S5.

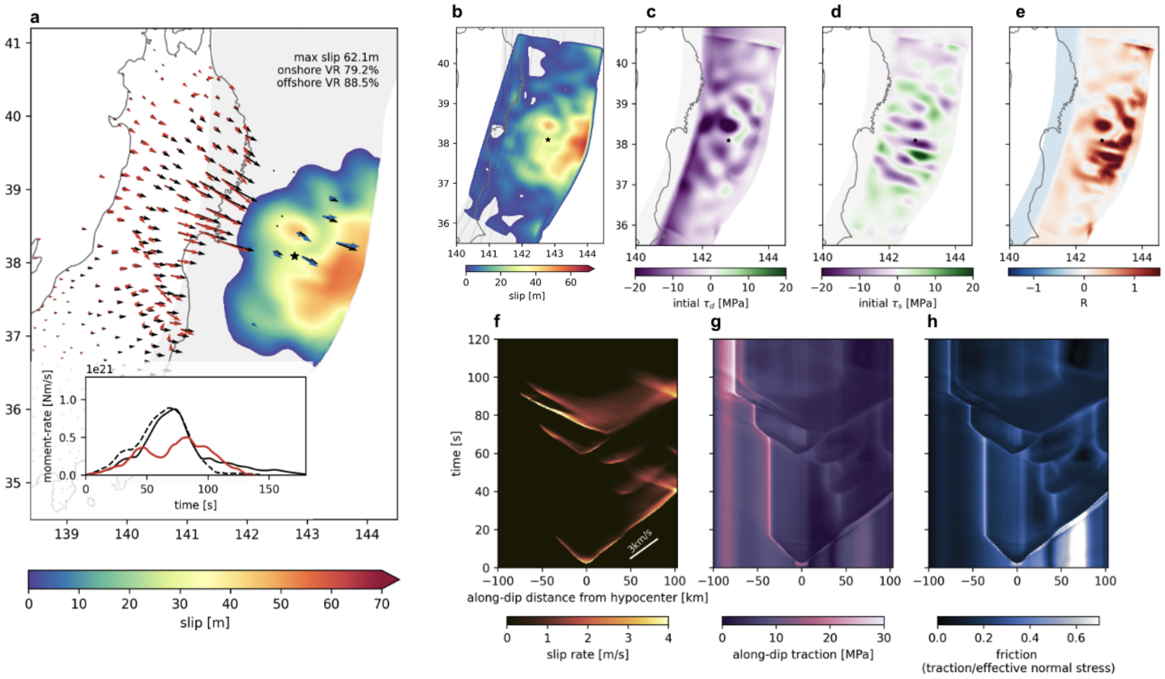

Figure S26: **Dynamic rupture model using stress-change pattern derived from the finite-fault slip model of Melgar & Bock, 2016.** (a) Simulated slip distribution and corresponding geodetic deformation. Red and blue arrows indicate synthetic onshore and offshore deformation, respectively. Black arrows show the observations. The inset compares the simulated moment-rate function (red), with the USGS (solid black), and SCARDEC (dashed black) source model moment-rate functions [48, 61]. (b) Melgar & Bock (2016) finite-fault model slip distribution. (c-e) Distribution of initial along-dip shear stress (τ_d), along-strike shear stress (τ_s), and relative prestress ratio (R), from left to right, respectively. (f-h) Along dip profile of slip rate, along-dip traction, and effective friction coefficient, from left to right, respectively. See also Supplementary Figure S27 and Video S7.

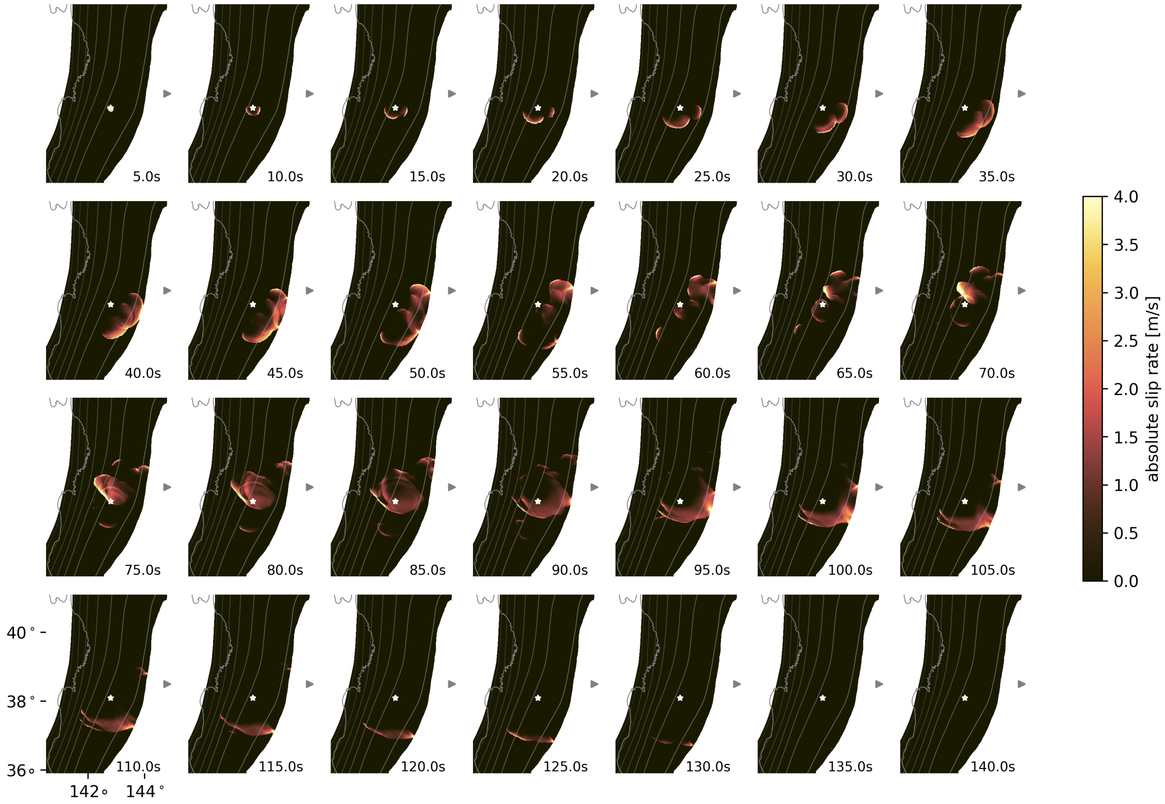

Figure S27: **Slip-rate evolution of dynamic rupture model using stress-change pattern derived from the finite-fault slip model of Melgar & Bock (2016).** Snapshots are shown at 5 s intervals from 5 s to 140 s (left to right, top to bottom). Colors indicate absolute slip rate. Contours outline the slab depth at 10 km intervals. The simulation reproduces repeated slip reactivation and mixed pulse-like and crack-like rupture styles. The strong prestress heterogeneity leads to updip reactivated slip pulses and less clear depth-dependent rupture style variability. See also Supplementary Figure S26 and Video S7.

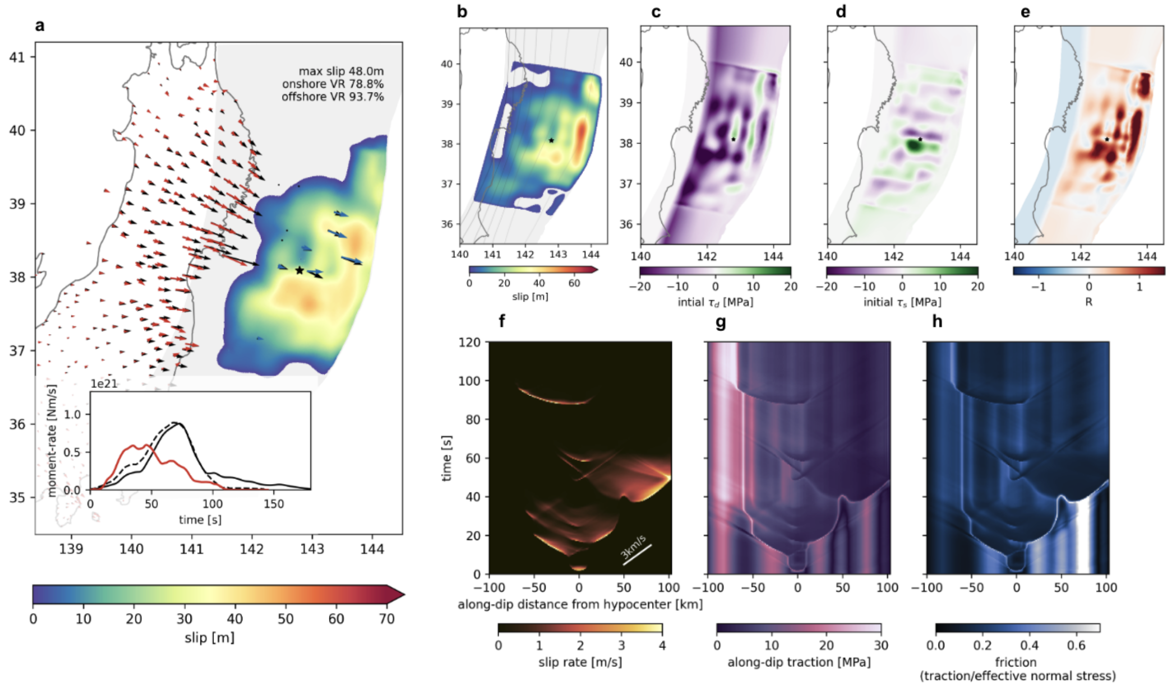

Figure S28: **Dynamic rupture model using stress-change pattern derived from the finite-fault slip model of Yamazaki et al. (2018).** (a) Simulated slip distribution and corresponding geodetic deformation. Red and blue arrows indicate synthetic onshore and offshore deformation, respectively. Black arrows show the observations. The inset compares the simulated moment-rate function (red), with the USGS (solid black), and SCARDEC (dashed black) source model moment-rate functions [48, 61]. (b) Yamazaki et al. (2018) finite-fault model slip distribution. (c-e) Distribution of initial along-dip shear stress (τ_d), along-strike shear stress (τ_s), and relative prestress ratio (R), from left to right, respectively. (f-h) Along dip profile of slip rate, along-dip traction, and effective friction coefficient, from left to right, respectively.See also Supplementary Figure S29 and Video S8.

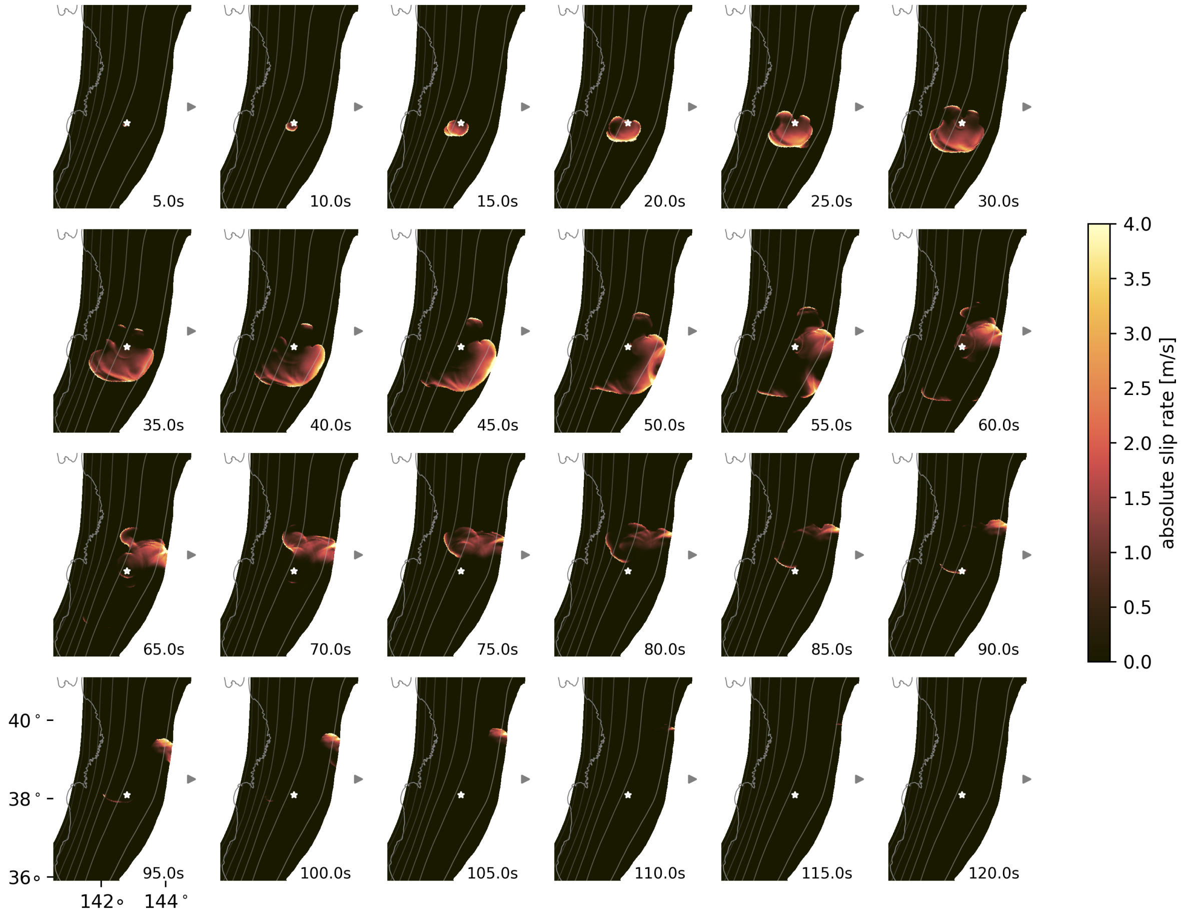

Figure S29: **Slip-rate evolution of dynamic rupture model using stress-change pattern derived from the finite-fault slip model of Yamazaki et al. (2018)**. Snapshots are shown at 5 s intervals from 5 s to 120 s (left to right, top to bottom). Colors indicate absolute slip rate. Contours outline the depth at 10 km intervals. The simulation reproduces repeated slip reactivation and mixed downdip pulse-like and updip crack-like rupture styles. See also Supplementary Figure S28 and Video S8.

Supplementary References

1. Wirth, E. A., Sahakian, V. J., Wallace, L. M. & Melnick, D. The Occurrence and Hazards of Great Subduction Zone Earthquakes. *Nature Reviews Earth & Environment* **3,** 125–140. doi:10.1038/s43017-021-00245-w (2022).
2. Lee, S.-J., Huang, B.-S., Ando, M., Chiu, H.-C. & Wang, J.-H. Evidence of Large Scale Repeating Slip during the 2011 Tohoku-Oki Earthquake: REPEATING SLIP DURING TOHOKU EARTHQUAKE. *Geophysical Research Letters* **38,** n/a–n/a. doi:10.1029/2011GL049580 (2011).
3. Ide, S., Baltay, A. & Beroza, G. C. Shallow Dynamic Overshoot and Energetic Deep Rupture in the 2011 M_w 9.0 Tohoku-Oki Earthquake. *Science* **332,** 1426–1429. doi:10.1126/science.1207020 (2011).
4. Melgar, D. & Bock, Y. Kinematic Earthquake Source Inversion and Tsunami Runup Prediction with Regional Geophysical Data. *Journal of Geophysical Research: Solid Earth* **120,** 3324–3349. doi:10.1002/2014JB011832 (2015).
5. Meng, L., Inbal, A. & Ampuero, J.-P. A Window into the Complexity of the Dynamic Rupture of the 2011 Mw 9 Tohoku-Oki Earthquake: THE 2011 TOHOKU-OKI EARTHQUAKE. *Geophysical Research Letters* **38,** n/a–n/a. doi:10.1029/2011GL048118 (2011).
6. Lay, T. *et al.* Depth-Varying Rupture Properties of Subduction Zone Megathrust Faults. *Journal of Geophysical Research: Solid Earth* **117.** doi:10.1029/2011JB009133 (2012).
7. Fujiwara, T. *et al.* The 2011 Tohoku-Oki Earthquake: Displacement Reaching the Trench Axis. *Science* **334,** 1240–1240. doi:10.1126/science.1211554 (2011).
8. Kodaira, S., Fujiwara, T., Fujie, G., Nakamura, Y. & Kanamatsu, T. Large Coseismic Slip to the Trench During the 2011 Tohoku-Oki Earthquake. *Annual Review of Earth and Planetary Sciences* **48,** 321–343. doi:10.1146/annurev-earth-071719-055216 (2020).
9. Uchida, N. & Bürgmann, R. A Decade of Lessons Learned from the 2011 Tohoku-Oki Earthquake. *Reviews of Geophysics* **59,** e2020RG000713. doi:10.1029/2020RG000713 (2021).
10. Wong, J. W. C., Fan, W. & Gabriel, A.-A. A Quantitative Comparison and Validation of Finite-Fault Models: The 2011 Tohoku-Oki Earthquake. *Journal of Geophysical Research: Solid Earth* **129,** e2024JB029212. doi:10.1029/2024JB029212 (2024).
11. Duan, B. Dynamic Rupture of the 2011 Mw 9.0 Tohoku-Oki Earthquake: Roles of a Possible Subducting Seamount. *Journal of Geophysical Research: Solid Earth* **117.** doi:10.1029/2011JB009124 (2012).
12. Ide, S. & Aochi, H. Historical Seismicity and Dynamic Rupture Process of the 2011 Tohoku-Oki Earthquake. *Tectonophysics. Great Earthquakes along Subduction Zones* **600,** 1–13. doi:10.1016/j.tecto.2012.10.018 (2013).
13. Kozdon, J. E. & Dunham, E. M. Rupture to the Trench: Dynamic Rupture Simulations of the 11 March 2011 Tohoku Earthquake. *Bulletin of the Seismological Society of America* **103,** 1275–1289. doi:10.1785/0120120136 (2013).
14. Huang, Y., Ampuero, J.-P. & Kanamori, H. Slip-Weakening Models of the 2011 Tohoku-Oki Earthquake and Constraints on Stress Drop and Fracture Energy. *Pure and Applied Geophysics* **171,** 2555–2568. doi:10.1007/s00024-013-0718-2 (2014).
15. Sallarès, V. & Ranero, C. R. Upper-Plate Rigidity Determines Depth-Varying Rupture Behaviour of Megathrust Earthquakes. *Nature* **576,** 96–101. doi:10.1038/s41586-019-1784-0 (2019).
16. Galvez, P., Petukhin, A., Irikura, K. & Somerville, P. Dynamic Source Model for the 2011 Tohoku Earthquake in a Wide Period Range Combining Slip Reactivation with the Short-Period Ground Motion Generation Process. *Pure and Applied Geophysics* **177,** 2143–2161. doi:10.1007/s00024-019-02210-7 (2020).

17. Ma, S. Wedge Plasticity and a Minimalist Dynamic Rupture Model for the 2011 MW 9.1 Tohoku-Oki Earthquake and Tsunami. *Tectonophysics* **869,** 230146. doi:10.1016/j.tecto.2023.230146 (2023).

18. Ramos, M. D., Thakur, P., Huang, Y., Harris, R. A. & Ryan, K. J. Working with Dynamic Earthquake Rupture Models: A Practical Guide. *Seismological Research Letters* **93,** 2096–2110. doi:10.1785/0220220022 (2022).

19. Ampuero, J.-P. & Rubin, A. M. Earthquake Nucleation on Rate and State Faults – Aging and Slip Laws. *Journal of Geophysical Research: Solid Earth* **113,** 2007JB005082. doi:10.1029/2007JB005082 (2008).

20. Ke, C.-Y., McLaskey, G. C. & Kammer, D. S. Rupture Termination in Laboratory-Generated Earthquakes. *Geophysical Research Letters* **45.** doi:10.1029/2018GL080492 (2018).

21. Lambert, V., Lapusta, N. & Perry, S. Propagation of Large Earthquakes as Self-Healing Pulses or Mild Cracks. *Nature* **591,** 252–258. doi:10.1038/s41586-021-03248-1 (2021).

22. Dieterich, J. H. Modeling of Rock Friction: 1. Experimental Results and Constitutive Equations. *Journal of Geophysical Research: Solid Earth* **84,** 2161–2168. doi:10.1029/JB084iB05p02161 (1979).

23. Ruina, A. Slip Instability and State Variable Friction Laws. *Journal of Geophysical Research: Solid Earth* **88,** 10359–10370. doi:10.1029/JB088iB12p10359 (1983).

24. Noda, H., Dunham, E. M. & Rice, J. R. Earthquake Ruptures with Thermal Weakening and the Operation of Major Faults at Low Overall Stress Levels. *Journal of Geophysical Research: Solid Earth* **114,** 2008JB006143. doi:10.1029/2008JB006143 (2009).

25. Di Toro, G. *et al.* Fault Lubrication during Earthquakes. *Nature* **471,** 494–498. doi:10.1038/nature09838 (2011).

26. Ujiie, K. *et al.* Low Coseismic Shear Stress on the Tohoku-Oki Megathrust Determined from Laboratory Experiments. *Science* **342,** 1211–1214. doi:10.1126/science.1243485 (2013).

27. Gabriel, A.-A., Ampuero, J.-P., Dalguer, L. A. & Mai, P. M. The Transition of Dynamic Rupture Styles in Elastic Media under Velocity-Weakening Friction. *Journal of Geophysical Research: Solid Earth* **117.** doi:10.1029/2012JB009468 (2012).

28. Ulrich, T., Gabriel, A.-A., Ampuero, J.-P. & Xu, W. Dynamic Viability of the 2016 Mw 7.8 Kaikōura Earthquake Cascade on Weak Crustal Faults. *Nature Communications* **10,** 1213. doi:10.1038/s41467-019-09125-w (2019).

29. Rubino, V., Lapusta, N. & Rosakis, A. J. Intermittent Lab Earthquakes in Dynamically Weakening Fault Gouge. *Nature* **606,** 922–929. doi:10.1038/s41586-022-04749-3 (2022).

30. Kammer, D. S. *et al.* Earthquake Energy Dissipation in a Fracture Mechanics Framework. *Nature Communications* **15,** 4736. doi:10.1038/s41467-024-47970-6 (2024).

31. Nishikawa, T. *et al.* The Slow Earthquake Spectrum in the Japan Trench Illuminated by the S-net Seafloor Observatories. *Science* **365,** 808–813. doi:10.1126/science.aax5618 (2019).

32. Bassett, D., Shillington, D. J., Wallace, L. M. & Elliott, J. L. Variation in Slip Behaviour along Megathrusts Controlled by Multiple Physical Properties. *Nature Geoscience.* doi:10.1038/s41561-024-01617-9 (2025).

33. Nishikawa, T., Ide, S. & Nishimura, T. A Review on Slow Earthquakes in the Japan Trench. *Progress in Earth and Planetary Science* **10,** 1. doi:10.1186/s40645-022-00528-w (2023).

34. Lapusta, N. & Rice, J. R. Nucleation and Early Seismic Propagation of Small and Large Events in a Crustal Earthquake Model. *Journal of Geophysical Research: Solid Earth* **108,** 2001JB000793. doi:10.1029/2001JB000793 (2003).

35. Cattania, C. Complex Earthquake Sequences On Simple Faults. *Geophysical Research Letters* **46,** 10384–10393. doi:10.1029/2019GL083628 (2019).

36. Barbot, S. Slow-Slip, Slow Earthquakes, Period-Two Cycles, Full and Partial Ruptures, and Deterministic Chaos in a Single Asperity Fault. *Tectonophysics* **768,** 228171. doi:10.1016/j.tecto.2019.228171 (2019).

37. Aochi, H. The 1999 Izmit, Turkey, Earthquake: Nonplanar Fault Structure, Dynamic Rupture Process, and Strong Ground Motion. *Bulletin of the Seismological Society of America* **93,** 1249–1266. doi:10.1785/0120020167 (2003).

38. Heidbach, O. *et al.* The World Stress Map database release 2016: Crustal stress pattern across scales. *Tectonophysics* **744,** 484–498 (2018).

39. Uphoff, C. *et al. Extreme Scale Multi-Physics Simulations of the Tsunamigenic 2004 Sumatra Megathrust Earthquake* in *Proceedings of the International Conference for High Performance Computing, Networking, Storage and Analysis* (ACM, Denver Colorado, 2017), 1–16. doi:10.1145/3126908.3126948.

40. Ulrich, T., Gabriel, A.-A. & Madden, E. H. Stress, Rigidity and Sediment Strength Control Megathrust Earthquake and Tsunami Dynamics. *Nature Geoscience* **15,** 67–73. doi:10.1038/s41561-021-00863-5 (2022).

41. Rice, J. R. Heating and Weakening of Faults during Earthquake Slip. *Journal of Geophysical Research: Solid Earth* **111.** doi:10.1029/2005JB004006 (2006).

42. Beeler, N. M., Tullis, T. E. & Goldsby, D. L. Constitutive Relationships and Physical Basis of Fault Strength Due to Flash Heating. *Journal of Geophysical Research: Solid Earth* **113.** doi:10.1029/2007JB004988 (2008).

43. Nielsen, S. & Madariaga, R. On the Self-Healing Fracture Mode. *Bulletin of the Seismological Society of America* **93,** 2375–2388. doi:10.1785/0120020090 (2003).

44. Saffer, D. M. & Marone, C. Comparison of Smectite- and Illite-Rich Gouge Frictional Properties: Application to the Updip Limit of the Seismogenic Zone along Subduction Megathrusts. *Earth and Planetary Science Letters* **215,** 219–235. doi:10.1016/S0012-821X(03)00424-2 (2003).

45. VallÃ, M. A New Database of Source Time Functions (STFs) Extracted from the SCARDEC Method. *Physics of the Earth and Planetary Interiors* (2016).

46. Yagi, Y. & Fukahata, Y. Rupture Process of the 2011 Tohoku-oki Earthquake and Absolute Elastic Strain Release. *Geophysical Research Letters* **38,** n/a–n/a. doi:10.1029/2011GL048701 (2011).

47. Brown, L., Wang, K. & Sun, T. Static Stress Drop in the M_w 9 Tohoku-oki Earthquake: Heterogeneous Distribution and Low Average Value. *Geophysical Research Letters* **42.** doi:10.1002/2015GL066361 (2015).

48. Hayes, G. P. Rapid Source Characterization of the 2011 M w 9.0 off the Pacific Coast of Tohoku Earthquake. *Earth, Planets and Space* **63,** 529–534. doi:10.5047/eps.2011.05.012 (2011).

49. Cochard, T. *et al.* Propagation of Extended Fractures by Local Nucleation and Rapid Transverse Expansion of Crack-Front Distortion. *Nature Physics* **20,** 660–665. doi:10.1038/s41567-023-02365-0 (2024).

50. Heaton, T. H. Evidence for and Implications of Self-Healing Pulses of Slip in Earthquake Rupture. *Physics of the Earth and Planetary Interiors* **64,** 1–20. doi:10.1016/0031-9201(90)90002-F (1990).

51. Perrin, G., Rice, J. R. & Zheng, G. Self-Healing Slip Pulse on a Frictional Surface. *Journal of the Mechanics and Physics of Solids* **43,** 1461–1495. doi:10.1016/0022-5096(95)00036-I (1995).

52. Sun, Y. & Cattania, C. Back-propagating Earthquakes on a Simple Fault. *Authorea Preprints.* doi:10.22541/essoar.173724475.50020741/v1 (2025).

53. Kurahashi, S. & Irikura, K. Short-Period Source Model of the 2011 Mw 9.0 Off the Pacific Coast of Tohoku Earthquake. *Bulletin of the Seismological Society of America* **103,** 1373–1393. doi:10.1785/0120120157 (2013).

54. Dunham, E. M. Dissipative interface waves and the transient response of a three-dimensional sliding interface with Coulomb friction. *Journal of the Mechanics and Physics of Solids* **53,** 327–357 (2005).

55. Vallée, M. *et al.* Self-Reactivated Rupture during the 2019 M = 8 Northern Peru Intraslab Earthquake. *Earth and Planetary Science Letters* **601,** 117886. doi:10.1016/j.epsl.2022.117886 (2023).

56. Ueda, H. *et al.* The Submarine Fault Scarp of the 2011 Tohoku-oki Earthquake in the Japan Trench. *Communications Earth & Environment* **4,** 476. doi:10.1038/s43247-023-01118-4 (2023).

57. Loveless, J. P. & Meade, B. J. Two Decades of Spatiotemporal Variations in Subduction Zone Coupling Offshore Japan. *Earth and Planetary Science Letters* **436,** 19–30. doi:10.1016/j.epsl.2015.12.033 (2016).

58. Zhang, K. *et al.* Complex Tsunamigenic Near-Trench Seafloor Deformation during the 2011 Tohoku–Oki Earthquake. *Nature Communications* **14,** 3260. doi:10.1038/s41467-023-38970-z (2023).

59. Barras, F., Thøgersen, K., Aharonov, E. & Renard, F. How Do Earthquakes Stop? Insights From a Minimal Model of Frictional Rupture. *Journal of Geophysical Research: Solid Earth* **128,** e2022JB026070. doi:10.1029/2022JB026070 (2023).

60. Satake, K., Fujii, Y., Harada, T. & Namegaya, Y. Time and Space Distribution of Coseismic Slip of the 2011 Tohoku Earthquake as Inferred from Tsunami Waveform Data. *Bulletin of the Seismological Society of America* **103,** 1473–1492. doi:10.1785/0120120122 (2013).

61. Vallée, M. Source Time Function Properties Indicate a Strain Drop Independent of Earthquake Depth and Magnitude. *Nature Communications* **4,** 2606. doi:10.1038/ncomms3606 (2013).

62. Yue, H. & Lay, T. Inversion of High-Rate (1 Sps) GPS Data for Rupture Process of the 11 March 2011 Tohoku Earthquake (M_w 9.1): INVERSION OF HIGH-RATE GPS FOR TOHOKU EQ. *Geophysical Research Letters* **38,** n/a–n/a. doi:10.1029/2011GL048700 (2011).

63. Okuwaki, R., Yagi, Y. & Hirano, S. Relationship between High-frequency Radiation and Asperity Ruptures, Revealed by Hybrid Back-projection with a Non-planar Fault Model. *Scientific Reports* **4,** 7120. doi:10.1038/srep07120 (2014).

64. Das, S. & Aki, K. A Numerical Study of Two-Dimensional Spontaneous Rupture Propagation. *Geophysical Journal International* **50,** 643–668. doi:10.1111/j.1365-246X.1977.tb01339.x (1977).

65. Zheng, G. & Rice, J. R. Conditions under Which Velocity-Weakening Friction Allows a Self-Healing versus a Cracklike Mode of Rupture. *Bulletin of the Seismological Society of America* **88,** 1466–1483. doi:10.1785/BSSA0880061466 (1998).

66. Hok, S., Fukuyama, E. & Hashimoto, C. Dynamic Rupture Scenarios of Anticipated Nankai-Tonankai Earthquakes, Southwest Japan. *Journal of Geophysical Research* **116,** B12319. doi:10.1029/2011JB008492 (2011).

67. Oral, E., Ampuero, J. P., Ruiz, J. & Asimaki, D. A Method to Generate Initial Fault Stresses for Physics-Based Ground-Motion Prediction Consistent with Regional Seismicity. *Bulletin of the Seismological Society of America* **112,** 2812–2827. doi:10.1785/0120220064 (2022).

68. Tinti, E. *et al.* Constraining Families of Dynamic Models Using Geological, Geodetic and Strong Ground Motion Data: The Mw 6.5, October 30th, 2016, Norcia Earthquake, Italy. *Earth and Planetary Science Letters* **576** (2021).

69. Yamazaki, Y., Cheung, K. F. & Lay, T. A Self-Consistent Fault Slip Model for the 2011 Tohoku Earthquake and Tsunami. *Journal of Geophysical Research: Solid Earth* **123,** 1435–1458. doi:10.1002/2017JB014749 (2018).

70. Kubota, T., Saito, T. & Hino, R. A New Mechanical Perspective on a Shallow Megathrust Near-Trench Slip from the High-Resolution Fault Model of the 2011 Tohoku-Oki Earthquake. *Progress in Earth and Planetary Science* **9,** 68. doi:10.1186/s40645-022-00524-0 (2022).

71. Ide, S. & Aochi, H. Earthquakes as Multiscale Dynamic Ruptures with Heterogeneous Fracture Surface Energy. *Journal of Geophysical Research: Solid Earth* **110.** doi:10.1029/2004JB003591 (2005).

72. Nielsen, S. B. & Carlson, J. M. Rupture Pulse Characterization: Self-Healing, Self-Similar, Expanding Solutions in a Continuum Model of Fault Dynamics. *Bulletin of the Seismological Society of America* **90,** 1480–1497. doi:10.1785/0120000021 (2000).

73. Ito, Y. *et al.* Frontal Wedge Deformation near the Source Region of the 2011 Tohoku-Oki Earthquake: FRONTAL WEDGE DEFORMATION OF JPN TRENCH. *Geophysical Research Letters* **38,** n/a–n/a. doi:10.1029/2011GL048355 (2011).

74. Moore, J. C., Plank, T. A., Chester, F. M., Polissar, P. J. & Savage, H. M. Sediment Provenance and Controls on Slip Propagation: Lessons Learned from the 2011 Tohoku and Other Great Earthquakes of the Subducting Northwest Pacific Plate. *Geosphere* **11,** 533–541. doi:10.1130/GES01099.1 (2015).

75. Tsuru, T. *et al.* Along-Arc Structural Variation of the Plate Boundary at the Japan Trench Margin: Implication of Interplate Coupling. *Journal of Geophysical Research: Solid Earth* **107,** ESE 11-1-ESE 11–15. doi:10.1029/2001JB001664 (2002).

76. Bassett, D. & Watts, A. B. Gravity Anomalies, Crustal Structure, and Seismicity at Subduction Zones: 2. Interrelationships between Fore-Arc Structure and Seismogenic Behavior. *Geochemistry, Geophysics, Geosystems* **16,** 1541–1576. doi:10.1002/2014GC005685 (2015).

77. Shi, Z. & Ben-Zion, Y. Dynamic Rupture on a Bimaterial Interface Governed by Slip-Weakening Friction. *Geophysical Journal International* **165,** 469–484. doi:10.1111/j.1365-246X.2006.02853.x (2006).

78. Scholz, C. H. The rupture mode of the shallow large-slip surge of the Tohoku-Oki earthquake. *Bulletin of the Seismological Society of America* **104,** 2627–2631 (2014).

79. Noda, H. & Lapusta, N. Stable Creeping Fault Segments Can Become Destructive as a Result of Dynamic Weakening. *Nature* **493,** 518–521. doi:10.1038/nature11703 (2013).

80. Schmedes, J., Archuleta, R. J. & Lavallée, D. Correlation of earthquake source parameters inferred from dynamic rupture simulations. *Journal of Geophysical Research: Solid Earth* **115** (2010).

81. Premus, J., Gallovič, F. & Ampuero, J.-P. Bridging Time Scales of Faulting: From Coseismic to Postseismic Slip of the Mw 6.0 2014 South Napa, California Earthquake. *Science Advances* **8,** eabq2536. doi:10.1126/sciadv.abq2536 (2022).

82. Schliwa, N., Gabriel, A.-A., Premus, J. & Gallovič, F. The Linked Complexity of Coseismic and Postseismic Faulting Revealed by Seismo-Geodetic Dynamic Inversion of the 2004 Parkfield Earthquake. *Journal of Geophysical Research: Solid Earth* **129,** e2024JB029410. doi:10.1029/2024JB029410 (2024).

83. Yao, S. & Yang, H. Rupture Dynamics of the 2012 Nicoya M_w 7.6 Earthquake: Evidence for Low Strength on the Megathrust. *Geophysical Research Letters* **47,** e2020GL087508. doi:10.1029/2020GL087508 (2020).

84. Prada, M. *et al.* The influence of depth-varying elastic properties of the upper plate on megathrust earthquake rupture dynamics and tsunamigenesis. *Journal of Geophysical Research: Solid Earth* **126,** e2021JB022328 (2021).

85. Ramos, M. D. *et al.* Assessing Margin-Wide Rupture Behaviors Along the Cascadia Megathrust With 3-D Dynamic Rupture Simulations. *Journal of Geophysical Research: Solid Earth* **126,** e2021JB022005. doi:10.1029/2021JB022005 (2021).

86. Chan, Y. P. B., Yao, S. & Yang, H. Impact of Hypocenter Location on Rupture Extent and Ground Motion: A Case Study of Southern Cascadia. *Journal of Geophysical Research: Solid Earth* **128,** e2023JB026371. doi:10.1029/2023JB026371 (2023).

87. Wirp, S. A., Gabriel, A.-A., Ulrich, T. & Lorito, S. Dynamic Rupture Modeling of Large Earthquake Scenarios at the Hellenic Arc Toward Physics-Based Seismic and Tsunami Hazard Assessment. *Journal of Geophysical Research: Solid Earth* **129,** e2024JB029320. doi:10.1029/2024JB029320 (2024).

88. Li, D. & Gabriel, A.-A. Linking 3D Long-Term Slow-Slip Cycle Models With Rupture Dynamics: The Nucleation of the 2014 Mw 7.3 Guerrero, Mexico Earthquake. *AGU Advances* **5,** e2023AV000979. doi:10.1029/2023AV000979 (2024).

89. Dumbser, M. & Käser, M. An Arbitrary High-Order Discontinuous Galerkin Method for Elastic Waves on Unstructured Meshes — II. The Three-Dimensional Isotropic Case. *Geophysical Journal International* **167,** 319–336. doi:10.1111/j.1365-246X.2006.03120.x (2006).

90. Heinecke, A. *et al. Petascale High Order Dynamic Rupture Earthquake Simulations on Heterogeneous Supercomputers* in *SC '14: Proceedings of the International Conference for High Performance Computing, Networking, Storage and Analysis* ISSN: 2167-4337 (2014), 3–14. doi:10.1109/SC.2014.6. https://ieeexplore.ieee.org/document/7012188 (2024).

91. Krenz, L. *et al. 3D Acoustic-Elastic Coupling with Gravity: The Dynamics of the 2018 Palu, Sulawesi Earthquake and Tsunami* in *Proceedings of the International Conference for High Performance Computing, Networking, Storage and Analysis* (ACM, St. Louis Missouri, 2021), 1–14. doi:10.1145/3458817.3476173.

92. Pelties, C., de la Puente, J., Ampuero, J.-P., Brietzke, G. B. & Käser, M. Three-Dimensional Dynamic Rupture Simulation with a High-Order Discontinuous Galerkin Method on Unstructured Tetrahedral Meshes. *Journal of Geophysical Research: Solid Earth* **117.** doi:10.1029/2011JB008857 (2012).

93. Pelties, C., Gabriel, A.-A. & Ampuero, J.-P. Verification of an ADER-DG Method for Complex Dynamic Rupture Problems. *Geoscientific Model Development* **7,** 847–866. doi:10.5194/gmd-7-847-2014 (2014).

94. Harris, R. A. *et al.* A Suite of Exercises for Verifying Dynamic Earthquake Rupture Codes. *Seismological Research Letters* **89,** 1146–1162. doi:10.1785/0220170222 (2018).

95. Taufiqurrahman, T., Gabriel, A.-A., Ulrich, T., Valentová, L. & Gallovič, F. Broadband Dynamic Rupture Modeling With Fractal Fault Roughness, Frictional Heterogeneity, Viscoelasticity and Topography: The 2016 M_{w} 6.2 Amatrice, Italy Earthquake. *Geophysical Research Letters* **49,** e2022GL098872. doi:10.1029/2022GL098872 (2022).

96. Breuer, A. & Heinecke, A. *Next-Generation Local Time Stepping for the ADER-DG Finite Element Method* in *2022 IEEE International Parallel and Distributed Processing Symposium (IPDPS)* (IEEE, Lyon, France, 2022), 402–413. doi:10.1109/IPDPS53621.2022.00046.

97. Koketsu, K., Miyake, H., Tanaka, Y., *et al.* A proposal for a standard procedure of modeling 3-D velocity structures and its application to the Tokyo metropolitan area, Japan. *Tectonophysics* **472,** 290–300 (2009).

98. Koketsu, K., Miyake, H. & Suzuki, H. *Japan integrated velocity structure model version 1* in *Proceedings of the 15th world conference on earthquake engineering* **1** (2012), 4.

99. Group, G. C. *GEBCO 2024 Grid* 2024. doi:doi:10.5285/1c44ce99-0a0d-5f4f-e063-7086abc0ea0f.

100. Fukuyama, E. Automated seismic moment tensor determination by using on-line broadband seismic waveforms [in Japanese with English abstract]. *J. Seismol. Soc. Jpn.* **51,** 149 (1998).

101. Day, S. M., Dalguer, L. A., Lapusta, N. & Liu, Y. Comparison of Finite Difference and Boundary Integral Solutions to Three-Dimensional Spontaneous Rupture. *Journal of Geophysical Research: Solid Earth* **110.** doi:10.1029/2005JB003813 (2005).

102. Goldsby, D. L. & Tullis, T. E. Flash Heating Leads to Low Frictional Strength of Crustal Rocks at Earthquake Slip Rates. *Science* **334,** 216–218. doi:10.1126/science.1207902 (2011).

103. Brodsky, E. E. *et al.* The State of Stress on the Fault Before, During, and After a Major Earthquake. *Annual Review of Earth and Planetary Sciences* **48,** 49–74. doi:10.1146/annurev-earth-053018-060507 (2020).

104. Ujiie, K. & Tsutsumi, A. High-Velocity Frictional Properties of Clay-Rich Fault Gouge in a Megasplay Fault Zone, Nankai Subduction Zone. *Geophysical Research Letters* **37.** doi:10.1029/2010GL046002 (2010).

105. Viesca, R. C. & Garagash, D. I. Ubiquitous Weakening of Faults Due to Thermal Pressurization. *Nature Geoscience* **8,** 875–879. doi:10.1038/ngeo2554 (2015).

106. Bizzarri, A. & Cocco, M. A Thermal Pressurization Model for the Spontaneous Dynamic Rupture Propagation on a Three-dimensional Fault: 1. Methodological Approach. *Journal of Geophysical Research: Solid Earth* **111,** 2005JB003862. doi:10.1029/2005JB003862 (2006).

107. Schmitt, S. V., Segall, P. & Dunham, E. M. Nucleation and Dynamic Rupture on Weakly Stressed Faults Sustained by Thermal Pressurization. *Journal of Geophysical Research: Solid Earth* **120,** 7606–7640. doi:10.1002/2015JB012322 (2015).

108. Wang, Y. & Day, S. M. Seismic Source Spectral Properties of Crack-like and Pulse-like Modes of Dynamic Rupture. *Journal of Geophysical Research: Solid Earth* **122,** 6657–6684. doi:10.1002/2017JB014454 (2017).

109. Perry, S. M., Lambert, V. & Lapusta, N. Nearly Magnitude-Invariant Stress Drops in Simulated Crack-Like Earthquake Sequences on Rate-and-State Faults with Thermal Pressurization of Pore Fluids. *Journal of Geophysical Research: Solid Earth* **125,** e2019JB018597. doi:10.1029/2019JB018597 (2020).

110. Palgunadi, K. H., Gabriel, A.-A., Garagash, D. I., Ulrich, T. & Mai, P. M. Rupture Dynamics of Cascading Earthquakes in a Multiscale Fracture Network. *Journal of Geophysical Research: Solid Earth* **129,** e2023JB027578. doi:10.1029/2023JB027578 (2024).

111. Fulton, P. M. *et al.* Low Coseismic Friction on the Tohoku-Oki Fault Determined from Temperature Measurements. *Science* **342,** 1214–1217. doi:10.1126/science.1243641 (2013).

112. Dunham, E. M., Belanger, D., Cong, L. & Kozdon, J. E. Earthquake Ruptures with Strongly Rate-Weakening Friction and Off-Fault Plasticity, Part 1: Planar Faults. *Bulletin of the Seismological Society of America* **101,** 2296–2307. doi:10.1785/0120100075 (2011).

113. Lambert, V. & Lapusta, N. Absolute Stress Levels in Models of Low-Heat Faults: Links to Geophysical Observables and Differences for Crack-like Ruptures and Self-Healing Pulses. *Earth and Planetary Science Letters* **618,** 118277. doi:10.1016/j.epsl.2023.118277 (2023).

114. Dieterich, J. H. & Kilgore, B. D. Direct Observation of Frictional Contacts: New Insights for State-Dependent Properties. *Pure and Applied Geophysics PAGEOPH* **143,** 283–302. doi:10.1007/BF00874332 (1994).

115. Dieterich, J. H. Earthquake Nucleation on Faults with Rate-and State-Dependent Strength. *Tectonophysics* **211,** 115–134. doi:10.1016/0040-1951(92)90055-B (1992).

116. Ikari, M. J. & Kopf, A. J. Cohesive Strength of Clay-Rich Sediment. *Geophysical Research Letters* **38.** doi:10.1029/2011GL047918 (2011).

117. Ikari, M. J., Kameda, J., Saffer, D. M. & Kopf, A. J. Strength Characteristics of Japan Trench Borehole Samples in the High-Slip Region of the 2011 Tohoku-Oki Earthquake. *Earth and Planetary Science Letters* **412,** 35–41. doi:10.1016/j.epsl.2014.12.014 (2015).

118. Madden, E. H., Ulrich, T. & Gabriel, A.-A. The State of Pore Fluid Pressure and 3-D Megathrust Earthquake Dynamics. *Journal of Geophysical Research: Solid Earth* **127,** e2021JB023382. doi:10.1029/2021JB023382 (2022).

119. Saffer, D. M. & Tobin, H. J. Hydrogeology and Mechanics of Subduction Zone Forearcs: Fluid Flow and Pore Pressure. *Annual Review of Earth and Planetary Sciences* **39,** 157–186. doi:10.1146/annurev-earth-040610-133408 (2011).

120. Jamali Hondori, E. & Park, J.-O. Connection between High Pore-Fluid Pressure and Frictional Instability at Tsunamigenic Plate Boundary Fault of 2011 Tohoku-Oki Earthquake. *Scientific Reports* **12,** 12556. doi:10.1038/s41598-022-16578-5 (2022).

121. Hardebeck, J. L. & Okada, T. Temporal Stress Changes Caused by Earthquakes: A Review. *Journal of Geophysical Research: Solid Earth* **123,** 1350–1365. doi:10.1002/2017JB014617 (2018).

122. Hardebeck, J. L. & Loveless, J. P. Creeping Subduction Zones Are Weaker than Locked Subduction Zones. *Nature Geoscience* **11,** 60–64. doi:10.1038/s41561-017-0032-1 (2018).

123. Andrews, D. J. Rupture Dynamics with Energy Loss Outside the Slip Zone. *Journal of Geophysical Research: Solid Earth* **110.** doi:10.1029/2004JB003191 (2005).

124. Wollherr, S., Gabriel, A.-A. & Uphoff, C. Off-Fault Plasticity in Three-Dimensional Dynamic Rupture Simulations Using a Modal Discontinuous Galerkin Method on Unstructured Meshes: Implementation, Verification and Application. *Geophysical Journal International* **214,** 1556–1584. doi:10.1093/gji/ggy213 (2018).

125. Templeton, E. L. & Rice, J. R. Off-Fault Plasticity and Earthquake Rupture Dynamics: 1. Dry Materials or Neglect of Fluid Pressure Changes. *Journal of Geophysical Research: Solid Earth* **113.** doi:10.1029/2007JB005529 (2008).

126. Gabriel, A.-A., Ampuero, J.-P., Dalguer, L. A. & Mai, P. M. Source Properties of Dynamic Rupture Pulses with Off-fault Plasticity. *Journal of Geophysical Research: Solid Earth* **118,** 4117–4126. doi:10.1002/jgrb.50213 (2013).

127. Ma, S. A Physical Model for Widespread Near-Surface and Fault Zone Damage Induced by Earthquakes. *Geochemistry, Geophysics, Geosystems* **9.** doi:10.1029/2008GC002231 (2008).

128. Ma, S. & Nie, S. Dynamic Wedge Failure and Along-Arc Variations of Tsunamigenesis in the Japan Trench Margin. *Geophysical Research Letters* **46,** 8782–8790. doi:10.1029/2019GL083148 (2019).

129. Gallovič, F., Valentová, Ľ., Ampuero, J.-P. & Gabriel, A.-A. Bayesian Dynamic Finite-Fault Inversion: 1. Method and Synthetic Test. *Journal of Geophysical Research: Solid Earth* **124,** 6949–6969. doi:10.1029/2019JB017510 (2019).

130. Yang, H., Yao, S., He, B. & Newman, A. V. Earthquake rupture dependence on hypocentral location along the Nicoya Peninsula subduction megathrust. *Earth and Planetary Science Letters* **520,** 10–17. doi:https://doi.org/10.1016/j.epsl.2019.05.030 (2019).

131. Glehman, J. *et al.* Partial Ruptures Governed by the Complex Interplay between Geodetic Slip Deficit, Rigidity, and Pore Fluid Pressure in 3D Cascadia Dynamic Rupture Simulations (2024).

132. Guatteri, M. & Spudich, P. What can strong-motion data tell us about slip-weakening fault-friction laws? *Bulletin of the Seismological Society of America* **90,** 98–116 (2000).

133. Weng, H. & Yang, H. Constraining Frictional Properties on Fault by Dynamic Rupture Simulations and Near-Field Observations. *Journal of Geophysical Research: Solid Earth* **123,** 6658–6670. doi:10.1029/2017JB015414 (2018).

134. Jia, Z. *et al.* The Complex Dynamics of the 2023 Kahramanmaraş, Turkey, M_w 7.8-7.7 Earthquake Doublet. *Science* **381,** 985–990. doi:10.1126/science.adi0685 (2023).

135. Hayek, J. N. *et al.* Non-Typical Supershear Rupture: Fault Heterogeneity and Segmentation Govern Unilateral Supershear and Cascading Multi-Fault Rupture in the 2021 7.4 Maduo Earthquake. *Geophysical Research Letters* **51,** e2024GL110128. doi:https://doi.org/10.1029/2024GL110128 (2024).

136. Taufiqurrahman, T. *et al.* Dynamics, Interactions and Delays of the 2019 Ridgecrest Rupture Sequence. *Nature* **618,** 308–315. doi:10.1038/s41586-023-05985-x (2023).

137. Galvez, P., Ampuero, J.-P., Dalguer, L. A., Somala, S. N. & Nissen-Meyer, T. Dynamic Earthquake Rupture Modelled with an Unstructured 3-D Spectral Element Method Applied to the 2011 M9 Tohoku Earthquake. *Geophysical Journal International* **198,** 1222–1240. doi:10.1093/gji/ggu203 (2014).

138. Galvez, P., Dalguer, L. A., Ampuero, J.-P. & Giardini, D. Rupture Reactivation during the 2011 M_w 9.0 Tohoku Earthquake: Dynamic Rupture and Ground-Motion Simulations. *Bulletin of the Seismological Society of America* **106,** 819–831. doi:10.1785/0120150153 (2016).

139. Huang, Y., Meng, L. & Ampuero, J.-P. A Dynamic Model of the Frequency-Dependent Rupture Process of the 2011 Tohoku-Oki Earthquake. *Earth, Planets and Space* **64,** 1061–1066. doi:10.5047/eps.2012.05.011 (2012).

140. Hayek, J. N. *et al.* Non-Typical Supershear Rupture: Fault Heterogeneity and Segmentation Govern Unilateral Supershear and Cascading Multi-Fault Rupture in the 2021 Mw ${M}_{w}$7.4 Maduo Earthquake. *Geophysical Research Letters* **51,** e2024GL110128. doi:10.1029/2024GL110128 (2024).

141. Weng, H. & Ampuero, J.-P. The Dynamics of Elongated Earthquake Ruptures. *Journal of Geophysical Research: Solid Earth* **124,** 8584–8610. doi:10.1029/2019JB017684 (2019).

142. Day, S. M., Yu, G. & Wald, D. J. Dynamic stress changes during earthquake rupture. *Bulletin of the Seismological Society of America* **88,** 512–522. doi:10.1785/BSSA0880020512 (1998).

143. Tinti, E., Spudich, P. & Cocco, M. Earthquake Fracture Energy Inferred from Kinematic Rupture Models on Extended Faults. *Journal of Geophysical Research: Solid Earth* **110,** 2005JB003644. doi:10.1029/2005JB003644 (2005).

144. Causse, M., Dalguer, L. A. & Mai, P. M. Variability of dynamic source parameters inferred from kinematic models of past earthquakes. *Geophysical Journal International* **196,** 1754–1769. doi:10.1093/gji/ggt478 (2014).

145. Koper, K. D., Hutko, A. R. & Lay, T. Along-Dip Variation of Teleseismic Short-Period Radiation from the 11 March 2011 Tohoku Earthquake (Mw 9.0). *Geophysical Research Letters* **38.** doi:10.1029/2011GL049689 (2011).

146. Yagi, Y., Nakao, A. & Kasahara, A. Smooth and Rapid Slip near the Japan Trench during the 2011 Tohoku-oki Earthquake Revealed by a Hybrid Back-Projection Method. *Earth and Planetary Science Letters* **355–356,** 94–101. doi:10.1016/j.epsl.2012.08.018 (2012).

147. Yao, H., Shearer, P. M. & Gerstoft, P. Compressive Sensing of Frequency-Dependent Seismic Radiation from Subduction Zone Megathrust Ruptures. *Proceedings of the National Academy of Sciences* **110,** 4512–4517. doi:10.1073/pnas.1212790110 (2013).

148. Cocco, M. *et al.* Fracture Energy and Breakdown Work During Earthquakes. *Annual Review of Earth and Planetary Sciences* **51,** 217–252. doi:10.1146/annurev-earth-071822-100304 (2023).

149. Gabriel, A.-A., Garagash, D. I., Palgunadi, K. H. & Mai, P. M. Fault Size–Dependent Fracture Energy Explains Multiscale Seismicity and Cascading Earthquakes. *Science* **385,** eadj9587. doi:10.1126/science.adj9587 (2024).

150. Lambert, V. & Lapusta, N. Rupture-Dependent Breakdown Energy in Fault Models with Thermo-Hydro-Mechanical Processes. *Solid Earth* **11,** 2283–2302. doi:10.5194/se-11-2283-2020 (2020).

151. Gabriel, A.-A. *et al. SeisSol* version v1.3.2. 2025. doi:10.5281/zenodo.15685917. https://doi.org/10.5281/zenodo.15685917.

152. Sagiya, T. A decade of GEONET: 1994-2003 The continuous GPS observation in Japan and its impact on earthquake studies. *Earth, planets and space* **56,** xxix–xli (2004).

153. Oeser, J., Bunge, H.-P. & Mohr, M. *Cluster design in the earth sciences tethys* in *International conference on high performance computing and communications* (2006), 31–40.

154. Käser, M. & Dumbser, M. An Arbitrary High-Order Discontinuous Galerkin Method for Elastic Waves on Unstructured Meshes — I. The Two-Dimensional Isotropic Case with External Source Terms. *Geophysical Journal International* **166,** 855–877. doi:10.1111/j.1365-246X.2006.03051.x (2006).

155. Lay, T., Ammon, C. J., Kanamori, H., Xue, L. & Kim, M. J. Possible Large Near-Trench Slip during the 2011 M w 9.0 off the Pacific Coast of Tohoku Earthquake. *Earth, Planets and Space* **63,** 687–692. doi:10.5047/eps.2011.05.033 (2011).

156. Ide, S. Estimation of Radiated Energy of Finite-Source Earthquake Models. *Bulletin of the Seismological Society of America* **92,** 2994–3005. doi:10.1785/0120020028 (2002).

157. Aochi, H. & Ide, S. Conceptual Multi-Scale Dynamic Rupture Model for the 2011 off the Pacific Coast of Tohoku Earthquake. *Earth, Planets and Space* **63,** 761–765. doi:10.5047/eps.2011.05.008 (2011).

158. Lay, T. A Review of the Rupture Characteristics of the 2011 Tohoku-oki Mw 9.1 Earthquake. *Tectonophysics* **733,** 4–36. doi:10.1016/j.tecto.2017.09.022 (2018).

159. Wang, K. *et al.* Learning from Crustal Deformation Associated with the M9 2011 Tohoku-oki Earthquake. *Geosphere* **14,** 552–571. doi:10.1130/GES01531.1 (2018).

160. Fujii, Y., Satake, K., Sakai, S., Shinohara, M. & Kanazawa, T. Tsunami Source of the 2011 off the Pacific Coast of Tohoku Earthquake. *Earth, Planets and Space* **63,** 815–820. doi:10.5047/eps.2011.06.010 (2011).

161. Sun, T., Wang, K., Fujiwara, T., Kodaira, S. & He, J. Large Fault Slip Peaking at Trench in the 2011 Tohoku-oki Earthquake. *Nature Communications* **8,** 14044. doi:10.1038/ncomms14044 (2017).

162. Hossen, M. J., Cummins, P. R., Dettmer, J. & Baba, T. Tsunami Waveform Inversion for Sea Surface Displacement Following the 2011 Tohoku Earthquake: Importance of Dispersion and Source Kinematics. *Journal of Geophysical Research: Solid Earth* **120,** 6452–6473. doi:10.1002/2015JB011942 (2015).

163. Dettmer, J. *et al.* Tsunami Source Uncertainty Estimation: The 2011 Japan Tsunami. *Journal of Geophysical Research: Solid Earth* **121,** 4483–4505. doi:10.1002/2015JB012764 (2016).

164. Andrews, D. J. Rupture Velocity of Plane Strain Shear Cracks. *Journal of Geophysical Research* **81,** 5679–5687. doi:10.1029/JB081i032p05679 (1976).

165. Hu, F., Oglesby, D. D. & Chen, X. The Sustainability of Free-Surface-Induced Supershear Rupture on Strike-Slip Faults. *Geophysical Research Letters* **46,** 9537–9543. doi:10.1029/2019GL084318 (2019).
