# Supplementary material for: Dynamic restrengthening and fault heterogeneity explain megathrust earthquake complexity
Source: Nat Commun. 2026 Apr 27;17:5777. doi: 10.1038/s41467-026-71722-3 (PMC13323747; doi:10.1038/s41467-026-71722-3)
Supplement: Supplementary file 2 — Description of Additional Supplementary File [file 41467_2026_71722_MOESM2_ESM.pdf]

Description of Additional Supplementary Files

- **Supplementary Video S1:** Preferred dynamic rupture model

- **Supplementary Video S2:** Dynamic rupture model with prestress heterogeneity and simple reactivation rupture style

- Supplementary Video S3: Dynamic rupture model with homogeneous regional prestress condition

- **Supplementary Video S4:** Dynamic rupture model with prestress heterogeneity and multiscale heterogeneity in the state-evolution distance

- Supplementary Video S5: Dynamic rupture model with heterogeneous distribution of fully-weakened dynamic frictional strength and homogeneous depthdependent initial stress

- Supplementary Video S6: Dynamic rupture model using the stress-change pattern derived from the finite-fault model of Kubota et al. (2022)

- Supplementary Video S7: Dynamic rupture model using the stress-change pattern derived from the finite-fault model of Melgar et al. (2015)

- Supplementary Video S8: Dynamic rupture model using the stress-change pattern derived from the finite-fault model of Yamazaki et al. (2018)
