# Supplementary material for: Dynamic restrengthening and fault heterogeneity explain megathrust earthquake complexity
Source: Nat Commun. 2026 Apr 27;17:5777. doi: 10.1038/s41467-026-71722-3 (PMC13323747; doi:10.1038/s41467-026-71722-3)
Supplement: Supplementary file 11 — Transparent Peer Review file [file 41467_2026_71722_MOESM11_ESM.pdf]

nature portfolio

Peer Review File

Dynamic restrengthening and fault heterogeneity explain megathrust earthquake complexity

Corresponding Author: Mr Jeremy Wing Ching Wong

Parts of this Peer Review File have been redacted as indicated to remove third-party material.

This file contains all reviewer reports in order by version, followed by all author rebuttals in order by version.

Version 0:

Reviewer comments:

Reviewer #1

(Remarks to the Author)
The 3D dynamical modeling of the 2011 Tohoku earthquake rupture presented in this study is an important update to the many studies of the rupture process of this Mw 9.1 earthquake. While there have been a few prior 2D dynamical models published, characterizing some of the features of the rupture influenced by dynamic rupture, this is the most comprehensive dynamic modeling of which I am aware, and the code and its implementation are impressive. The paper repeatedly makes the point that some of the observed rupture complexity previously documented for this event, such as depth-varying frequency dependence of seismic radiation, likely re-rupturing of some portions of the large-slip zone, and rupture extension with large fault slip all the way to the trench through a shallow potentially velocity strengthening regime, can in general be accounted for by spatially variable initial stress heterogeneity and rapid frictional re-strengthening of the slip zone. In this regard, the paper is strong and the conclusions are important arguments favoring dynamical rupture complexity being important in general. A major weakness of the argumentation that ensues is in the assertion that the frictional properties of the megathrust can be simply depth-dependent, i.e., without frictional asperities. This is intrinsically incompatible with the assumption that the initial stress heterogeneity is the result of some prior process, which is unspecified, that does not involve frictional heterogeneity along strike. I think this argumentation is just distracting and it is not an important addition to the study and I think it should be removed.

The most demanding aspect of dynamical modeling of large earthquake ruptures is that the initial stress conditions on the fault must be specified, in addition to multiple additional parameters that underlie simple regional maximum stress orientations, frictional laws for rapid weakening and strengthening, anelastic laws for the shallow environment, etc. The strategy of most dynamic rupture models has been to use independently-determined coseismic slip models obtained previously from various seismic, geodetic, and tsunami observations to specify associated prestress on the fault and in the surrounding medium in some fashion for the dynamical model. That approach is embraced here by drawing from a previous JGR study a median model from 32 published slip models (which intrinsically is explicitly not optimized to fit any particular data set), inferring stress associated with that slip model, and adding it to the much simpler depth-dependent maximum stress orientations from the tectonic configuration. Then you allow dynamic failure of the fault with the many specified parameters, and you get a model that basically MUST look a lot like the median slip model that was used to set up the initial boundary conditions. Rather circular indeed; you had to know the answer to get a result, but still a sensible approach to parameterizing the model so that it comes out "OK", and one can then focus on dynamical rupture complexity that may accompany the rupture simulations under various weighting of the background and heterogeneous stress fields that drive the rupture. The problem, for me, is in then arguing that the heterogeneous stress inferred from slip models does not reflect frictional heterogeneity on the fault; no accounting is made for why the stress heterogeneity is configured the way that it is, and it is very different from the background tectonics stress. Is it the relic of residual stress from prior failures or is it a reflection of variable frictional properties, as might be expected for some 'asperity' on the fault surface? Since this slip-inferred heterogenous initial slip totally controls the configuration of the preferred model (rupture of just the background tectonic stress leads to whole-segment ruptures which violate the observations), it is highly misleading in my opinion to dismiss the most logical cause for the stress heterogeneity imposed in the initial conditions without providing a viable alternative. While dynamics of the rupture are the result of complex failure of the initial stress state, I see no basis for arguing that it is not from laterally heterogeneous coupling, as indicated by variable interseismic locking measurements, afterslip patterns and by persistent localized slip heterogeneity in many large rupture repeats. The heterogenous pre-stress does not

just come about magically and controls the overall event completely.

Re-rupture of the hypocentral region for the 2011 event was perhaps first invoked in the Science paper by Ide et al. (2011). It is interesting to see the multiple rupture front failures that occur in the preferred model. A few of the many seismic studies of this event show rupture time history maps (e.g., Ide et al., 2011) or plots with subfault moment rate functions that indicate up to two moment release pulses from subfaults in the central portion of the fault (e.g., Yue and Lay, 2011), which provide direct support for multiple failures of the central slip zone, but only two pulses are apparent in those cases, not 4 to 5. It would be useful to compile the documentation of subfault moment rate histories to compare with the calculations for the preferred model here; the discussion relative to prior work is qualitative, and the reader should be shown what evidence there is from kinematic models for this complexity and its temporal pattern. As might be expected given the median slip model used to compute the pre-stress, the preferred model gives a mediocre fit to the on-shore and poor fit to the off-shore geodetic data, a pretty poor fit to the average moment rate function, and no prediction at all for other data, such a tsunami data, which cause the median model to have very high variance in the near-trench environment in the published JGR paper where the 32 models are considered. One must recognize that the median of 32 model derived from different data sets, some of which have no resolution of far off-shore slip, is potentially quite biased. Perhaps it would be good to present results in this study using the median of just the joint models in the JGR paper; that would convey the uncertainty in dynamic effects associated with uncertainty in the pre-stress imposed in the dynamic model.

In general, I found the writing clear and the model set-up and rupture behavior thorough and impressively complete. With suitable revision to address the concerns raised above, this will be a good contribution for Nature Communications.

Reviewer #2

(Remarks to the Author)
Comments to the author:
In this paper, the authors describe a sophisticated model with physical constraints that models the 2011 Tohoku-Oki earthquake. Previous models exist, but authors argue that their starting parameters are more realistic as they use realistic megathrust geometry and don’t need frictional asperities or other artifacts that previous work has used. Instead, they use rapid frictional strengthening that causes multiple separate slip pulses. The authors “preferred model” fits the data and observed rupture complexity well. The modelling work has been carried out well, and there are no wrong statements in my eyes. However, both the description of the model(s?) and the interpretation of the results lack clarity, making it difficult to decipher the scientific contribution of this paper.

General comments:
Maybe not fit for a relatively short format paper such as Nat Comm. The many references to additional figures and additional methods are really necessary to understand the science. I honestly got lost switching between main text, methods at the bottom of the main text, referring to figures in the supplementary file and for example truly have difficulty finding out what models figures S7 and S8 refer to. Unless the main text can be restructured in a way that it is more logical to follow and at least the basics can be understood without the supplementary information in the methods and separate supplementary file, I would not recommend this article for publication, despite that fact that I think it has a significant scientific contribution.

The introduction is quite unclear when reading it for the first time. It mentions a lot of different topics, but does not explain how they are related to the research. This makes it confusing to read.
Especially the parts about stress heterogeneities should be worded more carefully, because on the one hand you describe this model is better than previous ones because it does not rely on prescribed frictional or stress heterogeneities, but then the incorporation of what you describe as ‘realistic’ stress heterogeneities is quite a vital part of the model. I guess you can try to get ‘realistic’ stress heterogeneities, but you still never know how well this represents the actual stress state of the megathrust.

The paper would benefit from a specific section with a short description of the model set-up in the main paper. It is quite unclear how many models you ran, how many variables you varied and to what degree and how you select a “preferred model”. Was the preferred model selected because it fits the measured seismic data the best, or because it shows the same “complexities”. This is not trivial, because many different models might show the same complexities for different reasons.

Related to this, is that it would help to 1) get better definitions of alpha and R0, 2) describe how they are varied and 3) specifically state how they influence the model outcomes. As it does seem to me that these are the important variables that are varied. Although I’m unsure how this relates to the rapid frictional healing, which is stated as an important parameter that is different in these models compared to other work. But this does not come back in the model description in the main paper. It is quite confusing that in figure 1 R0 varies from 0 to 1, and in figure 6 R0 is only varied from 0.12 to 0.20.

The fact that slip becomes pulse-like when the model prescribes rapid re-strengthening should not be surprising. An explanation to what degree the input parameters of the dynamic weakening are responsible for the observed behavior would be good. Especially as this is one of the main distinguishing factors between this model and other models.

In general there is very limited (or no) comparison between the models in this work and previous models, although there are several papers (some cited in this manuscript) that have also attempted to model this same earthquake.

The methods section at the bottom of the main paper contains a lot more information than just the methods and is almost a

separate paper. I have not provided detailed comments on this section of the manuscript.

Minor comments:
Lines 26-32: all true, but the link between the earthquake complexity and the larger than expected hazard should be specifically mentioned.

Lines 40-45: also all true, but not really related to the question at hand?
Lines 45-47: If this is already known, than it does not have to be investigated with these models any more. I think I understand where you want to go with this paragraph, explaining that you try to explain the Tohoku-Oki EQ complexity with dynamic weakening/strengthening. But it would be nice if I wouldn't have to guess where you are going with this paper, but if you could make it more obvious. Same is basically true for the entire introduction. After re-reading it after I've read the entire paper twice, it is clear to me what you are trying to say.

Lines 76-83: From this paragraph, it sounds as if you do vary the initial stress and fault strength conditions, but that is not clear from the rest of the paper.

Lines 85-87: the descriptions/definitions of alpha and R0 are not quite clear to me. Especially R0, as fault strength and maximum stress drop are quite vague terms. Also it is defined in line 84/85 as: "regionally constrained ratio of fault strength over maximum possible stress drop" and in figure caption 1c as: "maximum possible stress drop over frictional stress drop". That does not sound as the same definition to me.

Line 101: pulse-like and crack-like slip should should be defined. With just the description pulse-like I immediately thought of a wrinkle-pulse as described in Scholz 2014 ("The rupture mode of the shallow large-slip surge of the Tohoku-Oki Earthquake"), but these kind of pulses generally only propagate updip, not downdip as described in this model. Also, as Scholz' paper describes a different model that explains the "complexities" of the Tohoku-Oki earthquake, the authors might consider briefly discussing why their model is better than this explanation.

Lines 94-99 and lines 101-111: the description of the "preferred model" result is split over two separate sections, "Results" and "Dynamic rupture …", with some repetition between the two. Re-structuring this into one clearly defined section (similar as one section with the model description) would help tremendously for ease of understanding.

Line 112: "Our model explains the observed contrast …" but this explanation only works if you can actually explain why downdip propagation is pulse-like and updip propagation is crack-like, which is not obvious from the manuscript up to this point, or at all. It just states that is does happen, which is not an explanation.

Line 142: "... High-frequency radiation inversely correlates with total slip." yet the sentences above seem to suggest that pulse-like slip has higher frequency content than crack-like slip. So this seems to contradict your own statement.

Line 130-148: along with a better definition of crack-like and pulse-like as suggested above, it would help to explain here how these different slip modes related to seismic radiation frequency content. Especially as the amplitude and frequency content of the slip speed content seems to be used as well as the frequency and amplitude of the seismic radiation, which makes it confusing in places which one is referred to. E.G. in line 146 it is the frequency of seismic radiation, but in line 147 the slip is occurring at low frequencies, so what is frequencies here?

Line 159: "dynamic frictional weakening sustains shallow rupture propagation". As far as I know, the physical mechanisms that are thought to be responsible for dynamic weakening only occur in lithified rocks. Is there any evidence dynamic frictional weakening occurs in shallow sediments?

Line 190-192: The objective of the paper that is described here "how rupture evolution changes with varying R0 and alpha" does not reflect the rest of the paper. If this is the objective, a large part of the paper should be rewritten.

Lines 192-195: Again, it would be good to know why exactly the preferred model is preferred.

Line 196 and figure 6: How is S defined and what does it mean?

Line 210: Well yes, you get pulse-like ruptures when you have rapid dynamic weakening and strengthening. If you model it to get pulses, you will see pulses. How exactly does this contribute to the debate whether earthquakes are predominantly cracklike or pulselike?

Line 216-218: Sure, your simulations can be extended in a hundred different ways, but either leave this out, or specify why these specific attributes would be beneficial for the scope of this work.

Captions of supplementary videos do not match the video files. For one the right and center are switched at least in video S1, and has shear traction instead of stress drop. Video S3 has mud, which I guess might mean effective friction coefficient? Why are shear stresses all negative, and do they increase? Also an annotation in the video of the starting conditions, or at least a descriptive title would be really helpful, because when I open all three it is difficult to keep track of which one is which.

Table 1: depth I assume is the range between this and the depth above? Depth ranges (0-3, 3-18) would have been clearer

Comments to the author:
In this paper, the authors describe a sophisticated model with physical constraints that models the 2011 Tohoku-Oki earthquake. This has been done before, but authors argue that their starting parameters are more realistic as they use realistic megathrust geometry and don't need frictional asperities or other artifacts that previous work has used. Instead, they use rapid frictional strengthening that causes multiple separate slip pulses. The authors "prefered model" fits the data and observed rupture complexity well. The modelling work has been carried out well, and there are no wrong statements in my eyes. However, both the description of the model(s?) and the interpretation of the results lack clarity, making it difficult to decipher the scientific contribution of this paper.

General comments:
Maybe not fit for a relatively short format paper such as Nat Comm. The many references to additional figures and additional methods are really necessary to understand the science. I honestly got lost switching between main text, methods at the bottom of the main text, referring to figures in the supplementary file and for example truly have difficulty finding out what models figures S7 and S8 refer to. Unless the main text can be restructured in a way that it is more logical to follow and at least the basics can be understood without the supplementary information in the methods and separate supplementary file, I would not recommend this article for publication, despite that fact that I think it has a significant scientific contribution.

The introduction is quite unclear when reading it for the first time. It mentions a lot of different topics, but does not explain how they are related to the research. This makes it confusing to read.
Especially the parts about stress heterogeneities should be worded more carefully, because on the one hand you describe this model is better than previous ones because it does not rely on prescribed frictional or stress heterogeneities, but then the incorporation of what you describe as 'realistic' stress heterogeneities is quite a vital part of the model. I guess you can try to get 'realistic' stress heterogeneities, but you still never know how well this represents the actual stress state of the megathrust.

The paper would benefit from a specific section with a short description of the model set-up in the main paper. It is quite unclear how many models you ran, how many variables you varied and to what degree and how you select a "preferred model". Was the preferred model selected because it fits the measured seismic data the best, or because it shows the same "complexities". This is not trivial, because many different models might show the same complexities for different reasons.

Related to this, is that it would help to 1) get better definitions of alpha and R0, 2) describe how they are varied and 3) specifically state how they influence the model outcomes. As it does seem to me that these are the important variables that are varied. Although I'm unsure how this relates to the rapid frictional healing, which is stated as an important parameter that is different in these models compared to other work. But this does not come back in the model description in the main paper. It is quite confusing that in figure 1 R0 varies from 0 to 1, and in figure 6 R0 is only varied from 0.12 to 0.20.

The fact that slip becomes pulse-like when the model prescribes rapid re-strengthening should not be surprising. An explanation to what degree the input parameters of the dynamic weakening are responsible for the observed behavior would be good. Especially as this is one of the main distinguishing factors between this model and other models.

The methods section at the bottom of the main paper contains a lot more information than just the methods and is almost a separate paper. I have not provided detailed comments on this section of the manuscript.

Minor comments:
Lines 26-32: all true, but the link between the earthquake complexity and the larger than expected hazard should be specifically mentioned.

Lines 40-45: also all true, but not really related to the question at hand?
Lines 45-47: If this is already known, than it does not have to be investigated with these models any more. I think I understand where you want to go with this paragraph, explaining that you try to explain the Tohoku-Oki EQ complexity with dynamic weakening/strengthening. But it would be nice if I wouldn't have to guess where you are going with this paper, but if you could make it more obvious. Same is basically true for the entire introduction. After re-reading it after I've read the entire paper twice, it is clear to me what you are trying to say.

Lines 76-83: From this paragraph, it sounds as if you do vary the initial stress and fault strength conditions, but that is not clear from the rest of the paper.

Lines 85-87: the descriptions/definitions of alpha and R0 are not quite clear to me. Especially R0, as fault strength and

maximum stress drop are quite vague terms. Also it is defined in line 84/85 as: “regionally constrained ratio of fault strength over maximum possible stress drop” and in figure caption 1c as: “maximum possible stress drop over frictional stress drop”. That does not sound as the same definition to me.

Line 101: pulse-like and crack-like slip should should be defined. With just the description pulse-like I immediately thought of a wrinkle-pulse as described in Scholz 2014 (“The rupture mode of the shallow large-slip surge of the Tohoku-Oki Earthquake”), but these kind of pulses generally only propagate updip, not downdip as described in this model. Also, as Scholz’ paper describes a different model that explains the “complexities” of the Tohoku-Oki earthquake, the authors might consider briefly discussing why their model is better than this explanation.

Lines 94-99 and lines 101-111: the description of the “preferred model” result is split over two separate sections, “Results” and “Dynamic rupture …”, with some repetition between the two. Re-structuring this into one clearly defined section (similar as one section with the model description) would help tremendously for ease of understanding.

Line 112: “Our model explains the observed contrast …” but this explanation only works if you can actually explain why downdip propagation is pulse-like and updip propagation is crack-like, which is not obvious from the manuscript up to this point, or at all. It just states that is does happen, which is not an explanation.

Line 142: “... High-frequency radiation inversely correlates with total slip.” yet the sentences above seem to suggest that pulse-like slip has higher frequency content than crack-like slip. So this seems to contradict your own statement.

Line 130-148: along with a better definition of crack-like and pulse-like as suggested above, it would help to explain here how these different slip modes related to seismic radiation frequency content. Especially as the amplitude and frequency content of the slip speed content seems to be used as well as the frequency and amplitude of the seismic radiation, which makes it confusing in places which one is referred to. E.G. in line 146 it is the frequency of seismic radiation, but in line 147 the slip is occurring at low frequencies, so what is frequencies here?

Line 159: “dynamic frictional weakening sustains shallow rupture propagation”. As far as I know, the physical mechanisms that are thought to be responsible for dynamic weakening only occur in lithified rocks. Is there any evidence dynamic frictional weakening occurs in shallow sediments?

Line 190-192: The objective of the paper that is described here “how rupture evolution changes with varying R0 and alpha” does not reflect the rest of the paper. If this is the objective, a large part of the paper should be rewritten.

Lines 192-195: Again, it would be good to know why exactly the preferred model is preferred.

Line 196 and figure 6: How is S defined and what does it mean?

Line 210: Well yes, you get pulse-like ruptures when you have rapid dynamic weakening and strengthening. If you model it to get pulses, you will see pulses. How exactly does this contribute to the debate whether earthquakes are predominantly cracklike or pulselike?

Line 216-218: Sure, your simulations can be extended in a hundred different ways, but either leave this out, or specify why these specific attributes would be beneficial for the scope of this work.

Captions of supplementary videos do not match the video files. For one the right and center are switched at least in video S1, and has shear traction instead of stress drop. Video S3 has mud, which I guess might mean effective friction coefficient? Why are shear stresses all negative, and do they increase? Also an annotation in the video of the starting conditions, or at least a descriptive title would be really helpful, because when I open all three it is difficult to keep track of which one is which.

Table 1: depth I assume is the range between this and the depth above? Depth ranges (0-3, 3-18) would have been clearer

Reviewer #3

(Remarks to the Author)
In this study, the authors conduct 3-D dynamic rupture simulations for the 2011 Tohoku Mw9.0 earthquake through considering the heterogeneous stress condition (with constraints from fault geometry and the stress drop pattern) and the rate- and state friction law (with strong weakening at high slip rate). The rupture model recovers the first-order rupture characteristics, including slip pattern, rupture duration, rupture speed, and moment. The authors claim that the preferred

model displays complex depth-dependent radiation (pulse or crack) and rupture reactivations as observed in the 2011 Tohoku earthquake. The topic of this study is intriguing, and the manuscript is well written. However, I have two major concerns regarding the reliability of the rupture model and the depth-dependent radiation, respectively. More validations are needed to support current conclusions. Therefore, I would recommend a major revision in this round. My comments are as below.

Major concerns:

1. The 2011 Tohoku Mw9.0 earthquake was well-recorded by the observational networks in Japan and worldwide, and many static and kinematic models have been obtained based on those data. Currently, the dynamic model in this study is only validated with static displacement data. Since this study focuses on the dynamic rupture processes of the 2011 Tohoku Mw9.0 earthquake, I would suggest validating the model with more seismic waveforms, such as local strong-motion data, and showing to which degree this model can capture the rupture processes.

Particularly, the authors suggest multiple rupture fronts rather than a smooth rupture front expanding (e.g., Figs. 6a and 6b). Given very different rupture processes, the two models should produce different ground motions at local strong motion stations. Then a comparison between the two models and the real data will help to validate the multiple rupture fronts.

Another related comment is that the variance reductions in the static displacement fitting are 77% and 55% onshore and offshore, respectively, which are not very high. Could the authors comment on the fitting performance? How reliable is the final slip distribution in this model?

2. The second major concern is regarding the high-frequency and low-frequency radiation in the model. Here the authors claim that the model reproduces the depth-dependent radiation pattern, as shown in figure 4. However, the shallow parts feature high peak slip rates (~ 5 m/s in Fig. 2c) and sharp slip rate rises (Fig. 4a), which can contribute to the high-frequency radiation. I feel like the current conclusion is not fully convincing to me. Here are my suggestions on conducting the radiation analysis in a more rigorous way.

i. The panel 4a shows the slip rate evolution at some points on the fault at different depths. However, the locations of those sampling points are unknown. In addition, the slip rate at depths shallower than 10 km is missing, which should host significant fault slip and radiation.

ii. In figure 4b, the spectra are normalized. Given the large slip at shallow depth, the amplitudes of the low-frequency spectral plateau should be much higher at shallow depths. Therefore, the normalization may cause apparently weak high-frequency radiation for shallow depths and bias the comparison of high-frequency radiation intensity at different depths. I would suggest the authors compare them without normalization (maybe taking the spectral ratio can better illustrate it).

iii. In panels c-e, the authors choose the period of 10s to filter the slip rates. To my best knowledge, high-frequency BP analyses are usually conducted in the frequency band of 0.5-2 Hz, such as in Lay et al. (2012 JGR) and Meng et al. (2011 GRL) for the Tohoku event. To make a fair comparison, I would suggest using a similar bandpass filter to show the pattern or show it in multiple frequency bands.

Minor comments:
1. Figure 1a: the 3-D view of the fault plane is a little bit hard to read. Could the authors refine the design of this panel?
2. Figure 5: it is hard to tell the location of the transition from rate-weakening to rate-strengthening. Changing the colorbar may help.

Version 1:

Reviewer comments:

Reviewer #1

(Remarks to the Author)
The authors have addressed the points that I made in my original review quite thoroughly. The manuscript is rich in information and the simulations are very impressive. My main concern about dynamical modeling is that it is so complex (many highly uncertain parameters and physical processes as well as uncertain prestress) that fitting of all types of data is not yet really viable, and the preferred model here remains very limited in even predicting the static geodetic offsets and gives only a first-order fit to the moment rate function, but that is the state of the art. I think the inclusion of additional heterogeneous prestress models in the revision is helpful; there is quite a strong sensitivity of the resulting models, even though the qualitatively complex ruptures do have some comparable features. This study is a very informative examination of some of the complex representations of prestress and frictional behavior that are intrinsic to dynamic modeling, but it remains unclear to me how to progress to models which actually match observations at the level of simple (possibly physically unrealistic) kinematic inversions. Clearly much is yet to be done. But this is an excellent study to guide the community. I recommend publication as is.

Thorne Lay

Reviewer #2

(Remarks to the Author)
The scientific merit of this work that I expected to be there after the first review still stands. It is a significant contribution to our understanding of megathrust earthquakes and the mechanisms behind it. I therefore think it worthy of publication in Nature Communications.
The main difference between this version and the previous version is in how well the work is explained. The authors also did incorporate some new sections and additional models. The main issue with the previous version was the lack of clarity on what exactly was done and how to interpret the results. So this mostly structural change and improved explanation is exactly what the manuscript needed. The paper is now a standalone paper that can be understood (mostly) without reading any supplementary information, which is exactly how it should be. Because some parts of the manuscript became a lot clearer to me, I do have some additional comments for further improvements, but these are mostly minor.

Response to previous comments:
- Most of my previous comments are resolved. The manuscript benefits from the additional length, which does justice to the complexity of the model.
- There is a big improvement in clarity, much easier to understand now. I appreciate the restructuring of the introduction, the added sections to the discussion and the additions of Table 1 and the list of supplementary figures.
- For one thing, it is now clear to me that the authors talk about dynamic (co-seismic) frictional weakening, not changes in rate-state friction parameters. It would help if authors can consistently call it dynamic weakening, which is the common term for this effect. It is (especially in the introduction and end of the paper) sometimes called " fast-velocity weakening" or "rapid velocity-weakening rate and state friction" (e.g. lines 48, 62, 76, 90, 381, also in methods line 470). This is confusing, especially as the fault is actually v-strengthening (positive a-b) on part of the fault surface. It would help if the authors can consistently call this dynamic (or co-seismic) weakening. The same with the coseismic restrengthening, which is the correct term, but sometimes it is called healing (e.g. subtitle line 148). Healing is a time-dependent process that is not incorporated into the model and not what the authors mean. It is clear to me now that the authors mean the rapid recovery in friction coefficient as the fault velocity drops, but calling it healing is confusing.
- One of my other major points was a lack of description of alpha and R0. This has much improved as well. I think I understand alpha now, it is a amplification factor of spatial heterogeneities from the mean slip model, where the location of heterogeneities comes directly from the model. So the location of asperities is not varied in this part, although with the new alternative models, there is variation in locations of the stress heterogeneities. This could be clearer in the main text, but is understandable to me from the methods now. I am still a little unsure where the actual numbers for R0 come from and how much the pre-stress levels actually vary in MPa's.

- Line 225-226 "expected behavior of clay-rich sediments: I'm glad the authors could correct me on my previous point 2.20 that sediments also have dynamic weakening. I learned something new today 😊. The text is now too specific though, because it only talks about clay-rich sediments. But most of your model is at depths (and stresses) where rocks are lithified. The value for f_w you use also better fits with the large amount of work that exists for lithified rock samples. On a side note, I do wonder if the large amount of fluids in the shallower parts might increase the amount of thermal pressurization hence dynamic weakening.

Other comments:
Major:
- From figure 1b it is clear how the 'normal' RSF formulation is varied, but this is not so well explained in the main text
- It is also not well explained in the main text if the full RS formulation is used, with state evolution etc. This does become clear from the methods, but a short sentence with reference to the methods would be nice.
- Why is slip accelerating near the trench? This should be unexpected as a-b becomes positive there? There is a section about large slip amount, off-fault plasticity and negative stress drop. But especially the acceleration of slip puzzles me. Can the authors comment on this too (would fit quite well in that section).
- Line 167: "Rapid coseismic restrengthening emerges as the principal mechanism controlling repeated reactivation: There is rapid coseismic restrengthening both updip and downdip, so this does not explain why there is only pulse-like behaviour downdip.
- Line 287 "self-healing": how is self-healing visible in the models? Is this the co-seismic increase of friction as rupture speed drops? Is it really healing?
- Line 344 "additional frictional heterogeneity", does this mean you have the frictional heterogeneity on top of the prestress heterogeneity? This is not clear from the main text.
- I do believe the methods could be a lot more to the point and with a lot less references. E.g. the depth-dependent effective normal stress, it would suffice to say this is based on a lithostatic gradient with a hydrostatic gradient of 0.9. Might be nice to add where you get the densities from, but all other references are unnecessary. Other section might be shortened similarly. This would make it easier to focus on the specifics that matter, like your model description.

Minor:
- In figure 3b, it seems to be the distance on the x axis is quite arbitrary, and the right edge is the trench? Would be nice if this could be annotated, easier for people who see the figure for the first time.
- Line 165 "slower apparent rupture speed": it seems to me the actual rupture speed is similar, but it is intermittent rather than continuous. I wonder if "slower apparent rupture speed" the right way of wording this.
- Line 269-271 "We next investigate .. ambient stress level": this is a bit confusing. It reads like: We measure a and b, which are alpha and R0, which represent b and a (reverse order). So which is which does not become clear from this sentence. Would be much clearer if in l 270 could be rewritten like: "depends on the amplitude of stress heterogeneity (alpha) and

relative prestress level (R0), by systematically varying both"
- Line 276 “Equation 16”, can you add this is in Methods? Because there is still methods and supplementary information.
- Line 401-403 “Similar rupture complexities … future megathrust earthquakes”. This is not mentioned in the discussion and should not be a conclusion of this work.
- SM1, SM2, and SM3 are not referred to at all. Are those necessary? I do think they could be incorporated in the methods in a shorter form.
- Supplementary figure S19 is a bit confusing, because the dynamic stress drop is velocity-dependent, not slip-dependent. By plotting this with slip on the x-axis, it almost looks like a slip-weakening mechanism, which is not what you use.
-

Reviewer #3

(Remarks to the Author)
I appreciate the authors' efforts in addressing my comments. In particular, I appreciate the new Figure 9, which is informative. The physical phenomenon raised in this study is interesting.

As the authors set the model particularly following the properties of the 2011 event, especially the initial stress condition, I suppose one of the targets of this study is to provide a reasonable rupture model for this event. However, the updated results of the frequency analysis and the comparison with the data are not yet sufficient to fully support the argument that the presented models really capture the physics of the 2011 Tohoku earthquake. I would suggest being more conservative/cautious about statements related to comparisons with the 2011 event and clarifying related limitations clearly. Here are my detailed comments.

1.I think the most important argument of this study is "the multiple rupture fronts emerging downdip are responsible for some intriguing observations in the 2011 Tohoku earthquake." I agree on the point that the presented model can explain the re-rupture process at the hypocenter region, which has been reported in previous literature. However, whether this mechanism can explain the depth-dependent radiation remains unclear to me. As shown in new Figures 4c and S6, the peak slip rate is comparable at shallow and deep portions at high frequency with just slight dependence on depth, which appears inconsistent with the reasonable trend of enhanced high-frequency energy shown in the spectra in Figure 4b. One potential explanation is that Figure 4b only captures the on-fault stations along the hypocentral dip profile. If the authors set the stations at other locations, such as with the latitude of ~39°, will the trend in Figure 4b still exist? I think this is very important to argue not only based on one profile but for the whole rupture. The authors argue with a paper (Fang et al., 2018).However, I think the depth-dependent trend is the same in most papers. So, it is important to argue for the trend over the whole rupture.

2. In this revision, the authors provide the comparison between the model synthetics and the strong motion data in Figure S8. I agree that the multiple-front rupture produces better fitting on the MYG006 and MYG014. But it appears to me that the fitting is overall not sufficient, as it cannot catch the long-period trends or amplitudes. Therefore, I have concerns about the argument in lines 253-254: "In addition, our model generates ground motions that can qualitatively fit the strong-ground motion records near the main rupture area (Supplementary Fig. S8)." I understand that for dynamic rupture models at such a large scale, it is very challenging to fit the data. However, such a level of fit may raise concerns for a 3-D model for a particular earthquake (with pre-constrained initial stress condition), which leads to questions about whether this model can really capture the rupture process to some degree.

3. Line 120 should be Figure S12.

Version 2:

Reviewer comments:

Reviewer #2

(Remarks to the Author)
The authors have been very thorough in addressing all my comments both in their response and in the corresponding changes to the manuscript. I would therefore recommend to accept this article as is.

The manuscript has much improved in readability since the first version. It is now much easier to understand also for people without an extensive modelling background (like me). The scientific contribution and added value for the community were there from the start.

Aagje Eijsink

Reviewer #3

(Remarks to the Author)
My comments have been well addressed in this round. I recommend it for publication in Nature Communications.

Open Access This Peer Review File is licensed under a Creative Commons Attribution 4.0 International License, which permits use, sharing, adaptation, distribution and reproduction in any medium or format, as long as you give appropriate credit to the original author(s) and the source, provide a link to the Creative Commons license, and indicate if changes were made.
In cases where reviewers are anonymous, credit should be given to 'Anonymous Referee' and the source.
The images or other third party material in this Peer Review File are included in the article's Creative Commons license, unless indicated otherwise in a credit line to the material. If material is not included in the article's Creative Commons license and your intended use is not permitted by statutory regulation or exceeds the permitted use, you will need to obtain permission directly from the copyright holder.
To view a copy of this license, visit https://creativecommons.org/licenses/by/4.0/

We thank Editor and three anonymous reviewers for the constructive evaluation of our manuscript.

In response to their comments, we have performed 28 new simulations. First, we have performed a new ensemble of four dynamic rupture simulations that account for multiscale frictional heterogeneity in state-evolution distance. Second, we performed another ensemble of six dynamic rupture simulations exploring the role of large-scale heterogeneity in the dynamic friction level. Third, we performed 18 new dynamic rupture simulations based on three different stress-change patterns inferred from three finite-fault slip models, those of Melgar et al. (2014), Yamazaki et al. (2018), and Kubota et al. (2022), which were not considered in our original manuscript. These newly added simulations support the robustness of our interpretation that the identified dynamic processes instead of particular heterogeneity patterns drive the rupture complexity.

We have also added a new analysis of modeled and observed ground motions. We have revised three main text figures and added 16 new supplementary figures, as well as five new supplementary animations. We updated the Zenodo repository with our newly added simulation input files.

We slightly revised the manuscript title to reflect our more general analysis. Nature Communications allows for a longer manuscript than Nature Geosciences, where our original

manuscript was submitted. Thus, we have extended the revised manuscript, including the Introduction, Results, and Discussion sections, and moved some of our Methods and Supplementary materials into the main text. This allows us to better clarify our model assumptions and address comments raised by the reviewers, including the assumed initial friction and stress heterogeneities. In response to the reviewers, we have added 9 references to better acknowledge previous work. We now slightly exceed the recommended maximum number of references for Nature Communications, as the Tohoku-Oki earthquake is among the best-studied events.

Please find below our response to the reviewers' comments. Reviewer's comments are in black, our responses are in blue, and changes to the manuscript are in red. The line numbers refer to the "clean" version of the revised manuscript.

Thank you for considering our revised manuscript for publication in Nature Communications.

Jeremy Wing Ching Wong, Alice-Agnes Gabriel, Wenyuan Fan

Reviewer #1:

R1.1 The 3D dynamical modeling of the 2011 Tohoku earthquake rupture presented in this study is an important update to the many studies of the rupture process of this Mw 9.1 earthquake. While there have been a few prior 2D dynamical models published, characterizing some of the features of the rupture influenced by dynamic rupture, this is the most comprehensive dynamic modeling of which I am aware, and the code and its implementation are impressive. The paper repeatedly makes the point that some of the observed rupture complexity previously documented for this event, such as depth-varying frequency dependence of seismic radiation, likely re-rupturing of some portions of the large-slip zone, and rupture extension with large fault slip all the way to the trench through a shallow potentially velocity strengthening regime, can in general be accounted for by spatially variable initial stress heterogeneity and rapid frictional re-strengthening of the slip zone. In this regard, the paper is strong and the conclusions are important arguments favoring dynamical rupture complexity being important in general.

We thank the reviewer for the detailed summary.

A major weakness of the argumentation that ensues is in the assertion that the frictional properties of the megathrust can be simply depth-dependent, i.e., without frictional asperities. This is intrinsically incompatible with the assumption that the initial stress heterogeneity is the result of some prior process, which is unspecified, that does not involve frictional heterogeneity along strike. I think this argumentation is just distracting and it is not an important addition to the study and I think it should be removed.

We thank the reviewer for this thoughtful comment. We agree that subduction zones likely host multiple sources of heterogeneity. The heterogeneous prestress used in our original models may serve as a proxy for other forms of fault heterogeneity, such as variations in frictional strength. In response to this comment, we performed two additional sets of simulations with frictional heterogeneities, totaling 10 new dynamic rupture simulations. We discuss the preferred scenario for each ensemble, which is defined as the model that yields the highest variance reduction relative to the geodetic observations and the SCARDEC seismic moment-rate function (Vallée and Douet, 2016).

The first new set of four simulations incorporates multiscale heterogeneity in the frictional state-evolution distance, following Ide & Aochi, 2005 (the preferred model out of these 4 simulations is shown in new Supplementary Figures S11-12, and Video S4). The second set of six simulations maps the heterogeneity informed from the median slip distributions onto the dynamic friction level while maintaining a uniform initial shear stress, following the approach of Tinti et al. (2021) (Supplementary Fig. S13, the preferred model is shown in the new Supplementary Figure S14-15 and Video S5).

Both new simulations with frictional heterogeneity yield similar levels of rupture complexity as the preferred model, including repeated reactivation, mixed pulse- and crack-like rupture modes, and large shallow slip. These results are discussed in a **new Discussion section:** ***Frictional***

heterogeneity and illustrated in newly added supplementary Figures S11-S15 and Videos S4 and S5.

We also revised the title and abstract to reflect our more general findings (see tracked changes). We have revised our Introduction (L47-56) to remove any potentially misleading statements.

References:
Ide, S., & Aochi, H. (2005). Earthquakes as multiscale dynamic ruptures with heterogeneous fracture surface energy. *Journal of Geophysical Research: Solid Earth,* 110(B11).

Tinti, E., Casarotti, E., Ulrich, T., Taufiqurrahman, T., Li, D., & Gabriel, A. A. (2021). Constraining families of dynamic models using geological, geodetic and strong ground motion data: The Mw 6.5, October 30th, 2016, Norcia earthquake, Italy. *Earth and Planetary Science Letters*, 576, 117237.

Vallée, M., & Douet, V. (2016). A new database of source time functions (STFs) extracted from the SCARDEC method. *Physics of the Earth and Planetary Interiors*, 257, 149-157.

R1.2 The most demanding aspect of dynamical modeling of large earthquake ruptures is that the initial stress conditions on the fault must be specified, in addition to multiple additional parameters that underlie simple regional maximum stress orientations, frictional laws for rapid weakening and strengthening, anelastic laws for the shallow environment, etc. The strategy of most dynamic rupture models has been to use independently-determined coseismic slip models obtained previously from various seismic, geodetic, and tsunami observations to specify associated prestress on the fault and in the surrounding medium in some fashion for the dynamical model. That approach is embraced here by drawing from a previous JGR study a median model from 32 published slip models (which intrinsically is explicitly not optimized to fit any particular data set), inferring stress associated with that slip model, and adding it to the much simpler depth-dependent maximum stress orientations from the tectonic configuration. Then you allow dynamic failure of the fault with the many specified parameters, and you get a model that basically MUST look a lot like the median slip model that was used to set up the initial boundary conditions. Rather circular indeed; you had to know the answer to get a result, but still a sensible approach to parameterizing the model so that it comes out "OK", and one can then focus on dynamical rupture complexity that may accompany the rupture simulations under various weighting of the background and heterogeneous stress fields that drive the rupture. The problem, for me, is in then arguing that the heterogeneous stress inferred from slip models does not reflect frictional heterogeneity on the fault; no accounting is made for why the stress heterogeneity is configured the way that it is, and it is very different from the background tectonics stress. Is it the relic of residual stress from prior failures or is it a reflection of variable frictional properties, as might be expected for some 'asperity' on the fault surface? Since this slip-inferred heterogenous initial slip totally controls the configuration of the preferred model (rupture of just the background tectonic stress leads to whole-segment ruptures which violate

the observations), it is highly misleading in my opinion to dismiss the most logical cause for the stress heterogeneity imposed in the initial conditions without providing a viable alternative. While dynamics of the rupture are the result of complex failure of the initial stress state, I see no basis for arguing that it is not from laterally heterogeneous coupling, as indicated by variable interseismic locking measurements, afterslip patterns and by persistent localized slip heterogeneity in many large rupture repeats. The heterogenous pre-stress does not just come about magically and controls the overall event completely.

We thank the reviewer for this constructive comment, which we have addressed by adding a new Discussion section clarifying the origin and role of prestress heterogeneity (see L. 283-292). We also mention alternative approaches to constrain prestress for dynamic rupture simulations (L. 284-286). We agree that defining initial stress conditions is one of the most important aspects of initializing dynamic rupture modeling (e.g., Ramos et al., 2022). Prestress heterogeneity may arise from many factors, such as past ruptures on the same or nearby faults, fault geometric complexities, local variations in fault strength or pore-fluid pressure, or spatial variations in tectonic loading. The coseismic slip distribution reflects such heterogeneities and has therefore been widely used to constrain the initial stress distribution for dynamic rupture models (e.g., Weng and Yang, 2018; Guatteri and Spudich, 2000; Tinti et al. 2021, Jia et al. 2023, Hayek et al., 2024). However, the "*dynamic viability*" of a given finite-fault slip model, that is, whether its slip distribution is physically consistent with earthquake dynamics, is not guaranteed.

It is important to clarify that prestress heterogeneity modulates but does not uniquely control rupture dynamics (L287-292). Prestress heterogeneity alone cannot determine the final slip distribution, and some finite slip models may be dynamically implausible (e.g., Tinti et al., 2021). Dynamic rupture evolution is governed by the nonlinear interaction between frictional evolution, stress redistribution, and seismic wave propagation (e.g., Kammer et al., 2024), rather than by static initial conditions alone, including prestress.

This is illustrated by our newly added dynamic rupture simulation that uses the stress-change pattern derived from the finite-fault slip model of Kubota et al. (2022) (see also response R1.4). Despite a patch of pronounced high prestress in the south (New Supplementary Figure S23), the resulting rupture does not reproduce the southern slip extension observed in the slip inversion. Instead, dynamic rupture preferentially propagates updip and northward, resulting in a smoother slip distribution (Fig. S23a) and premature spontaneous rupture arrest (e.g., Fig. S24).

In response to **R1.4** below, we discuss the new dynamic rupture scenarios using different prestress heterogeneity patterns derived from three published finite-fault models.

<u>References</u>:

Guatteri, M., & Spudich, P. (2000). What can strong-motion data tell us about slip-weakening fault-friction laws?. *Bulletin of the Seismological Society of America*, 90(1), 98-116.

Hayek, J. N., Marchandon, M., Li, D., Pousse-Beltran, L., Hollingsworth, J., Li, T., & Gabriel, A. A. (2024). Non-Typical Supershear Rupture: Fault Heterogeneity and Segmentation Govern Unilateral Supershear and Cascading Multi-Fault Rupture in the 2021 Mw 7.4 Maduo Earthquake. *Geophysical Research Letters*, 51(20), e2024GL110128.

Jia, Z., Jin, Z., Marchandon, M., Ulrich, T., Gabriel, A. A., Fan, W., ... & Fialko, Y. (2023). The complex dynamics of the 2023 Kahramanmaraş, Turkey, M w 7.8-7.7 earthquake doublet. *Science*, 381(6661), 985-990.

Kammer, D. S., McLaskey, G. C., Abercrombie, R. E., Ampuero, J. P., Cattania, C., Cocco, M., ... & Tinti, E. (2024). Earthquake energy dissipation in a fracture mechanics framework. *Nature communications*, 15(1), 4736.

Kubota, T., Saito, T., & Hino, R. (2022). A new mechanical perspective on a shallow megathrust near-trench slip from the high-resolution fault model of the 2011 Tohoku-Oki earthquake. *Progress in Earth and Planetary Science*, 9(1), 68.

Ramos, M. D., Thakur, P., Huang, Y., Harris, R. A., & Ryan, K. J. (2022). Working with dynamic earthquake rupture models: A practical guide. *Seismological Society of America*, 93(4), 2096-2110.

Tinti, E., Casarotti, E., Ulrich, T., Taufiqurrahman, T., Li, D., & Gabriel, A. A. (2021). Constraining families of dynamic models using geological, geodetic and strong ground motion data: The Mw 6.5, October 30th, 2016, Norcia earthquake, Italy. *Earth and Planetary Science Letters*, 576, 117237.

Weng, H., & Yang, H. (2018). Constraining frictional properties on fault by dynamic rupture simulations and near-field observations. *Journal of Geophysical Research: Solid Earth*, 123(8), 6658-6670.

R1.3 Re-rupture of the hypocentral region for the 2011 event was perhaps first invoked in the Science paper by Ide et al. (2011). It is interesting to see the multiple rupture front failures that occur in the preferred model. A few of the many seismic studies of this event show rupture time history maps (e.g., Ide et al., 2011) or plots with subfault moment rate functions that indicate up to two moment release pulses from subfaults in the central portion of the fault (e.g., Yue and Lay, 2011), which provide direct support for multiple failures of the central slip zone, but only two pulses are apparent in those cases, not 4 to 5. It would be useful to compile the documentation of subfault moment rate histories to compare with the calculations for the preferred model here; the discussion relative to prior work is qualitative, and the reader should be shown what evidence there is from kinematic models for this complexity and its temporal pattern.

We thank the reviewer for this helpful suggestion. We have added a quantitative discussion in comparison to previous work (L. 244-255). We added a new comparison of the along-dip

slip-rate evolution between our preferred dynamic rupture model and the finite-fault model of Melgar et al, (2015) in the **new Discussion section: *Comparison of simulated rupture reactivation with observations*** and in the new Figure 8. The comparison reveals first-order similarities, with major rupture episodes propagating updip during the first 40s and subsequently back-propagating downdip between 50 and 80s. When low-pass filtered at 30s, consistent with the spectral decay of finite-fault slip-rate functions, our simulated slip-rate evolution closely reproduces the inverted patterns.

The finite-fault model of Melgar et al. (2015) resolves only two to three rupture episodes in the hypocentral region, consistent with previous kinematic studies by Ide et al. 2011 and Yue and Lay 2011. This simpler representation of re-rupturing likely reflects the limited frequency range of the seismic data used in the inversion (typically up to 0.1 Hz), the use of long and simplified basis slip-rate functions (e.g., triangular functions of 10-20s, and smoothing regularization applied in most kinematic models (e.g., Ide et al., 2011, Yue & Lay, 2011, Bletery et al., 2014, Melgar & Bock, 2015). These factors reduce sensitivity to short-duration, high-frequency slip pulses at depth. The slip-rate spectra of Melgar & Bock 2015 decay strongly above the 30s period (Rebuttal Letter Figure R1). This spectral decay is much lower than the dominant frequency range of the down-dip episodic slip pulses (< 10s) in our dynamic rupture model (see Figure 4 and our response to **R3.8**). Low-pass filtering our dynamic rupture model yields similar results as observed in Melgar et al. (2015). In summary, multiple episodes of slip pulses at depth may not be uniquely resolved in common finite-fault inversions. In addition, our model provides a reasonable fit to the strong-ground motion records near the main rupture area (Supplementary Fig. S13). We included these results in a new section of the Discussion, in L. 244-254.

References:
Bletery, Q., Sladen, A., Delouis, B., Vallée, M., Nocquet, J. M., Rolland, L., & Jiang, J. (2014). A detailed source model for the Mw9. 0 Tohoku‐Oki earthquake reconciling geodesy, seismology, and tsunami records. Journal of Geophysical Research: Solid Earth, 119(10), 7636-7653.

Ide, S., Baltay, A., & Beroza, G. C. (2011). Shallow dynamic overshoot and energetic deep rupture in the 2011 M w 9.0 Tohoku-Oki earthquake. Science, 332(6036), 1426-1429.

Melgar, D., & Bock, Y. (2015). Kinematic earthquake source inversion and tsunami runup prediction with regional geophysical data. Journal of Geophysical Research: Solid Earth, 120(5), 3324-3349.

Yue, H., & Lay, T. (2011). Inversion of high‐rate (1 sps) GPS data for rupture process of the 11 March 2011 Tohoku earthquake (Mw 9.1). Geophysical Research Letters, 38(7).

REDACTED

R1.4 As might be expected given the median slip model used to compute the pre-stress, the preferred model gives a mediocre fit to the on-shore and poor fit to the off-shore geodetic data, a pretty poor fit to the average moment rate function, and no prediction at all for other data, such a tsunami data, which cause the median model to have very high variance in the near-trench environment in the published JGR paper where the 32 models are considered. One must recognize that the median of 32 models derived from different data sets, some of which have no resolution of far off-shore slip, is potentially quite biased. Perhaps it would be good to present results in this study using the median of just the joint models in the JGR paper; that would convey the uncertainty in dynamic effects associated with uncertainty in the pre-stress imposed in the dynamic model.

We thank the reviewer for highlighting the limitations of the median slip distribution, and we agree on the associated uncertainty in prestress heterogeneity, particularly near the trench. To address this, we conducted three additional sets of simulations using stress-change patterns informed from the finite-fault models of Melgar & Bock (2014), Yamazaki et al. (2018), and Kubota et al. (2022). All three models include tsunami datasets, with the first two jointly constrained by seismic, geodetic, and tsunami data. We include this comparison in the **new Disucssion section: *Dynamic rupture scenarios with prestress heterogeneity informed by alternative finite-fault slip models*** (L 294-313).

We perform six simulations per finite fault model, using the same approach as for the preferred model by mapping the corresponding stress change patterns onto the initial stress distribution. Within each ensemble, we vary the level of initial stress heterogeneity,**α,** and the regional tectonic stress, defined by $\mathbf{R_0}$, and identify alternative 'preferred' models as the ones that yield the highest variance reduction relative to the geodetic observations and SCARDEC moment-rates (Vallée and Douet, 2016). We detail these new models in a **new Methods section: *Dynamic rupture scenarios with prestress heterogeneity informed by alternative finite-fault slip models*** (L.653 - 659). In a new Discussion section of the main text, we discuss the 'preferred' scenario for each ensemble per finite-fault model.

Despite their contrasting prestress distributions, all three dynamic rupture simulations (new Supplementary Figures S21-28, Videos S6-8) reproduce comparable rupture characteristics of the preferred, median slip distribution-informed dynamic rupture model, including rupture reactivation, large shallow slip near the trench, and spontaneous rupture arrest. Notably, the joint inversion models of Melgar et al. (2016) and Yamazaki et al. (2018) show more complex slip distributions (see also Wong et al., 2024) and correspondingly result in stronger prestress heterogeneity. These dynamic rupture models produce more complex rupture evolution with multiple reactivation and predominantly pulse-like rupture across all depths.

These new simulations demonstrate that the rupture complexities identified in our preferred model arise robustly from the underlying dynamic processes and are consistent across different heterogeneous prestress configurations, informed by different finite fault models.

In general, I found the writing clear and the model set-up and rupture behavior thorough and impressively complete. With suitable revision to address the concerns raised above, this will be a good contribution for Nature Communications.

We thank the reviewer again for the constructive and thoughtful comments.

Reviewer #2 (Remarks to the Author):

Comments to the author:

R2.1 In this paper, the authors describe a sophisticated model with physical constraints that models the 2011 Tohoku-Oki earthquake. Previous models exist, but authors argue that their starting parameters are more realistic as they use realistic megathrust geometry and don't need frictional asperities or other artifacts that previous work has used. Instead, they use rapid frictional strengthening that causes multiple separate slip pulses. The authors "preferred model" fits the data and observed rupture complexity well. The modelling work has been carried out well, and there are no wrong statements in my eyes. However, both the description of the model(s?) and the interpretation of the results lack clarity, making it difficult to decipher the scientific contribution of this paper.

We thank the reviewer for the constructive comments. In the revised manuscript, we clarified the model description and highlighted the novelty better, and added 12 additional references to previous work. We note that our previous manuscript was submitted to Nature Geoscience, limiting references to 60. We have also moved key aspects of the model parameterization, previously located in the Methods and Supplementary Materials, into the main text to provide a clearer introduction to the model used in this study (see our responses below to your comments **R2.5–R2.6**). We reorganized and extended the Discussion into subsections and added systematic comparisons of alternative dynamic rupture models to strengthen the interpretation of our results.

General comments:
R2.2 Maybe not fit for a relatively short format paper such as Nat Comm. The many references to additional figures and additional methods are really necessary to understand the science. I honestly got lost switching between main text, methods at the bottom of the main text, referring to figures in the supplementary file and for example truly have difficulty finding out what models figures S7 and S8 refer to. Unless the main text can be restructured in a way that it is more logical to follow and at least the basics can be understood without the supplementary information in the methods and a separate supplementary file, I would not recommend this article for publication, despite the fact that I think it has a significant scientific contribution.

We thank the reviewer for this important comment. Our original manuscript was written for the more restrictive format of Nature Geoscience. In the revision, we have adapted it to the longer format allowed by Nature Communications.

We revised the *Results* section to include a concise description of the key methodological steps used to obtain the preferred model (L106-138). We increased the length of the paper from 2649 to 4710 words, better aligning with Nature Communications guidelines, allowing for up 5000 words of the main text. We revised the captions of all Figures to state which models are shown explicitly. Finally, we compiled a new overview list in the Supplementary Materials listing all

models explored in this study together with their associated figures and videos, to facilitate navigation and cross-referencing.

R2.3 The introduction is quite unclear when reading it for the first time. It mentions a lot of different topics, but does not explain how they are related to the research. This makes it confusing to read.

Thank you for the comments. We have streamlined the *Introduction* and better clarified how each topic raised connects to our study (see tracked changes). We now better highlight the novelty of our simulations in the *Introduction*, focusing on two main topics: the first model accounting for fast velocity weakening rate-and-state friction and observationally informed spatial heterogeneity (see L37–56).

R2.4 Especially the parts about stress heterogeneities should be worded more carefully, because on the one hand you describe this model is better than previous ones because it does not rely on prescribed frictional or stress heterogeneities, but then the incorporation of what you describe as 'realistic' stress heterogeneities is quite a vital part of the model. I guess you can try to get 'realistic' stress heterogeneities, but you still never know how well this represents the actual stress state of the megathrust.

We agree with the reviewer that the "true" stress state remains largely unconstrained, given the non-uniqueness of inversions and the resolving limits of existing observation networks. We have revised the manuscript to highlight and address the uncertainties in the inferred heterogeneity patterns and initial stress conditions (see the **new Discussion section: *Dynamic rupture scenarios with prestress heterogeneity informed by alternative finite-fault slip models*** *, L293–312*). We explored three new sets of simulations using prestress patterns inferred from the finite-fault models of Melgar & Bock (2015), Yamazaki et al. (2018), and Kubota et al. (2022) (New Supplementary Figs. S23-S28, Videos S6-S8). Despite their contrasting prestress heterogeneity patterns, all three models reproduce key rupture characteristics of the preferred, median slip distribution-informed dynamic rupture model, supporting our interpretations of the rupture complexity. Please also refer to our response to **R1.4** above.

R2.5 The paper would benefit from a specific section with a short description of the model set-up in the main paper. It is quite unclear how many models you ran, how many variables you varied and to what degree and how you select a "preferred model". Was the preferred model selected because it fits the measured seismic data the best, or because it shows the same "complexities". This is not trivial, because many different models might show the same complexities for different reasons.

Thank you for this suggestion. We moved the relevant materials from the *Methods* section to the main text. The revised *Results* section now includes the number of models explored, the parameter range explored, and the step size used in the grid search to select the preferred model (L106–124). To clarify the conditions controlling the rupture complexities, we have restructured the Discussion with a newly added model comparison that accounts for different

heterogeneity patterns and frictional asperities to identify the control mechanisms of the complexities (see our response in **R1.1** and **R1.4**).

R2.6 Related to this, is that it would help to 1) get better definitions of alpha and R0, 2) describe how they are varied and 3) specifically state how they influence the model outcomes. As it does seem to me that these are the important variables that are varied. Although I'm unsure how this relates to the rapid frictional healing, which is stated as an important parameter that is different in these models compared to other work. But this does not come back in the model description in the main paper. It is quite confusing that in figure 1 R0 varies from 0 to 1, and in figure 6 R0 is only varied from 0.12 to 0.20.

Thank you for the related comments. Together with our response to **R2.5** above, we have improved the definitions of $\boldsymbol{\alpha}$ and $\mathbf{R_0}$, clarified how they are varied in the model ensemble, and stated more explicitly how they influence the dynamic rupture outcomes (L.106–L115). We also now more clearly distinguish between the input scalar $\mathbf{R_0}$ and the spatially variable relative prestress ratio **R** shown in Figure 1. We added a new conceptual illustration in Supplementary Fig. S19 (see our response **R1.1** above).

We now clarify in the main text how the friction law governs rapid weakening and healing (L. 85–L97). The fast velocity-weakening rate-and-state friction law used here includes an inverse proportionality of frictional strength and slip rate, representing thermal weakening processes due to thermal effects that can operate on natural faults at high coseismic slip rates (Rice, 2006; Beeler et al., 2008). Dynamic rupture simulations governed by this friction law can take many rupture styles, including sub- or supershear crack-like and pulse-like rupture styles (e.g., Gabriel et al., 2012).

References:

Rice, J. R. (2006). Heating and weakening of faults during earthquake slip. *Journal of Geophysical Research: Solid Earth*, 111(B5).

Beeler, N. M., Tullis, T. E., & Goldsby, D. L. (2008). Constitutive relationships and physical basis of fault strength due to flash heating. *Journal of Geophysical Research: Solid Earth,* 113(B1).

Gabriel, A.-A., J.-P. Ampuero, L. A. Dalguer, P. M. Mai (2012), "The transition of dynamic rupture modes in elastic media", *J. Geophys. Res.*, 117(B9), 01480227, doi:10.1029/2012JB009468

R2.7 The fact that slip becomes pulse-like when the model prescribes rapid re-strengthening should not be surprising. An explanation to what degree the input parameters of the dynamic weakening are responsible for the observed behavior would be good. Especially as this is one of the main distinguishing factors between this model and other models.

We thank the reviewer for this comment. We agree that the parameters of our friction law can be tuned to describe the weakening mechanism between the two extreme behaviors, slip-weakening and velocity-weakening (e.g., Ampuero & Ben-Zion, 2008). In this work, we

follow previous laboratory and modeling studies in setting V_w= 0.1m/s, and f_w = 0.1 (Di Toro et al., 2011; Goldsby & Tullis, 2011; Uijie et al., 2013; Gabriel et al., 2012; Ulrich et al., 2019), and we now clarify in the main text that these frictional parameters are held fixed across all ensemble simulations (L101–102).

R2.8 In general there is very limited (or no) comparison between the models in this work and previous models, although there are several papers (some cited in this manuscript) that have also attempted to model this same earthquake.

In response to **R2.7,** we added a **new Discussion section:** ***Comparison to previous models of the Tohoku-Oki earthquake*** highlighting the difference with previous modeling studies.

R2.9 The methods section at the bottom of the main paper contains a lot more information than just the methods and is almost a separate paper. I have not provided detailed comments on this section of the manuscript.

Following the Reviewer's suggestions in **R2.5** and **R2.6,** we moved some parts of the previous Methods section into the main text (L85 –124).

Minor comments:
R2.10 Lines 26-32: all true, but the link between the earthquake complexity and the larger than expected hazard should be specifically mentioned.

Thank you for the comment. We have now removed this statement as quantifying hazard is not a direct focus of our study.

R2.11 Lines 40-45: also all true, but not really related to the question at hand?

Thank you for the comment. We have revised the Introduction overall to better align it with the scope of our paper (see tracked changes).

R2.12 Lines 45-47: If this is already known, than it does not have to be investigated with these models any more. I think I understand where you want to go with this paragraph, explaining that you try to explain the Tohoku-Oki EQ complexity with dynamic weakening/strengthening. But it would be nice if I wouldn't have to guess where you are going with this paper, but if you could make it more obvious. Same is basically true for the entire introduction. After re-reading it after I've read the entire paper twice, it is clear to me what you are trying to say.

Thank you also for this comment. In the revised Introduction, we have added more explicit statements regarding the addressed research gaps and the novelty of this study (see tracked changes in *Introduction*).

R2.13 Lines 76-83: From this paragraph, it sounds as if you do vary the initial stress and fault strength conditions, but that is not clear from the rest of the paper.

We revised this to focus on the initial stress to avoid confusion.

R2.14 Lines 85-87: the descriptions/definitions of alpha and R0 are not quite clear to me. Especially R0, as fault strength and maximum stress drop are quite vague terms. Also it is defined in line 84/85 as: “regionally constrained ratio of fault strength over maximum possible stress drop” and in figure caption 1c as: “maximum possible stress drop over frictional stress drop”. That does not sound as the same definition to me.

We unified the definition and clarified the differences between $\mathbf{R_0}$ and R value in the *Results* section (L112 – 115), see our response to **R2.5** and **R2.6**.

R2.15 Line 101: pulse-like and crack-like slip should be defined. With just the description pulse-like I immediately thought of a wrinkle-pulse as described in Scholz 2014 (“The rupture mode of the shallow large-slip surge of the Tohoku-Oki Earthquake”), but these kind of pulses generally only propagate updip, not downdip as described in this model. Also, as Scholz’ paper describes a different model that explains the “complexities” of the Tohoku-Oki earthquake, the authors might consider briefly discussing why their model is better than this explanation.

We thank the reviewer for this comment. We now define pulse-like and crack-like rupture explicitly in the Results section (L140–L142). Our simulation reproduce both updip and downdip rupture propagation with varying rupture speed, which is comparable to the observation (see our response to **R1.3**) and previous model studies (see our response to **R2.8**), and therefore distinct from the shallow, updip-propagating wrinkle pulses discussed by Scholz (2014).

We agree with the reviewer and Scholz (2014) that it would be interesting to study bimaterial effects and their potential contribution to the rupture dynamics of the Tohoku-Oki earthquake. Our present model employs a 1D velocity structure and thus does not resolve the material contrast across the plate interface. We now state this explicitly in the Model Limitations section (L365–367) and identify the inclusion of bimaterial effects as an important target for future 3D simulations.

<u>**References**</u>:
Scholz, C. H. (2014). The rupture mode of the shallow large-slip surge of the Tohoku-Oki earthquake. *Bulletin of the Seismological Society of America*, 104(5), 2627-2631.

R2.16 Lines 94-99 and lines 101-111: the description of the “preferred model” result is split over two separate sections, “Results” and “Dynamic rupture …”, with some repetition between the two. Re-structuring this into one clearly defined section (similar as one section with the model description) would help tremendously for ease of understanding.

Thank you for the comment. We have removed repetition to make the structure and flow clearer.

R2.17 Line 112: “Our model explains the observed contrast …” but this explanation only works if you can actually explain why downdip propagation is pulse-like and updip propagation is crack-like, which is not obvious from the manuscript up to this point, or at all. It just states that is does happen, which is not an explanation.

Thank you for the comment. We added an explanation in Lines 179–182.

R2.18 Line 142: “... High-frequency radiation inversely correlates with total slip.” yet the sentences above seem to suggest that pulse-like slip has higher frequency content than crack-like slip. So this seems to contradict your own statement.

We appreciate the reviewer’s detailed reading. There is no contradiction: our results show that the downdip pulse-like rupture is associated with higher-frequency radiation but with less total slip, whereas the shallow crack-like rupture produces larger slip with a lower frequency content. We clarified this in the Results section L173–179.

R2.19 Line 130-148: along with a better definition of crack-like and pulse-like as suggested above, it would help to explain here how these different slip modes related to seismic radiation frequency content. Especially as the amplitude and frequency content of the slip speed content seems to be used as well as the frequency and amplitude of the seismic radiation, which makes it confusing in places which one is referred to. E.G. in line 146 it is the frequency of seismic radiation, but in line 147 the slip is occurring at low frequencies, so what is frequencies here?

Thank you for the comments. In response to Reviewer 3’s comment **R3.7 (**below**)**, we updated the comparison of power spectral density between shallow and deep slip-rate functions, without normalization, to better compare their amplitude across different frequency ranges. The comparison (Updated Fig 4) illustrates that downdip pulse-like ruptures have higher frequency content than the updip large slip crack-like rupture. We also added the frequency range in L191–193.

R2.20 Line 159: “dynamic frictional weakening sustains shallow rupture propagation”. As far as I know, the physical mechanisms that are thought to be responsible for dynamic weakening only occur in lithified rocks. Is there any evidence dynamic frictional weakening occurs in shallow sediments?

Laboratory friction experiments on fault gouge recovered from the toe of the frontal prism in the area of large shallow slip during the 2011 Tohoku earthquake show pronounced dynamic weakening and low coseismic shear stress, demonstrating that dynamic frictional weakening can operate in shallow, poorly lithified sediments (Ujiie et al., 2013). We now state this explicitly in the revised manuscript (L212–215).

<u>References:</u>

Ujiie, K., Tanaka, H., Saito, T., Tsutsumi, A., Mori, J. J., Kameda, J., ... & Expedition 343 and 343T Scientists. (2013). Low coseismic shear stress on the Tohoku-Oki megathrust determined from laboratory experiments. *Science,* 342(6163), 1211-1214.

R2.21 Line 190-192: The objective of the paper that is described here “how rupture evolution changes with varying R0 and alpha” does not reflect the rest of the paper. If this is the objective, a large part of the paper should be rewritten.

We thank the reviewer for pointing out this confusing phrasing. To avoid confusion, we have revised the text in L256-258.

R2.22 Lines 192-195: Again, it would be good to know why exactly the preferred model is preferred.

As detailed in our response to the Reviewer’s comment **R2.5**, we have improved the description of the preferred model in the Results section.

R2.23 Line 196 and figure 6: How is S defined and what does it mean?

We have now clarified that S refers to the Seismic Ratio (Das & Aki, 1977), defined as the ratio of initial strength excess to maximum dynamic stress drop, as is now defined in Equation 16. We added the equation reference to the Figure 6 caption and L263–265.

Reference:
Das, S., & Aki, K. (1977). A numerical study of two-dimensional spontaneous rupture propagation. *Geophysical Journal International*, 50(3), 643-668.

R2.24 Line 210: Well yes, you get pulse-like ruptures when you have rapid dynamic weakening and strengthening. If you model it to get pulses, you will see pulses. How exactly does this contribute to the debate whether earthquakes are predominantly cracklike or pulselike?

As noted in our previous responses (**R2.7 and R2.8**), we agree that rapid dynamic weakening and restrengthening can lead to pulse-like rupture, however, in agreement with previous work, the rupture style is not prescribed: depending on prestress and heterogeneity, the same setup can produce crack-like rupture, too.

R2.25 Line 216-218: Sure, your simulations can be extended in a hundred different ways, but either leave this out, or specify why these specific attributes would be beneficial for the scope of this work.

As detailed in our response **R2.15** above, we have revised the Discussion in these lines.

R2.26 Captions of supplementary videos do not match the video files. For one the right and center are switched at least in video S1, and has shear traction instead of stress drop. Video S3 has mud, which I guess might mean effective friction coefficient? Why are shear stresses all negative, and do they increase? Also an annotation in the video of the starting conditions, or at least a descriptive title would be really helpful, because when I open all three it is difficult to keep track of which one is which.

Thank you for alerting us to this important typo, and we apologize for the resulting confusion. We have improved the videos accordingly. See also our response to **R2.2** above.

R2.27 Table 1: depth I assume is the range between this and the depth above? Depth ranges (0-3, 3-18) would have been clearer.

Thank you for the suggestion! We added the depth ranges to the “Values” column in Table 1.

Reviewer #3 (Remarks to the Author):

R3.1
In this study, the authors conduct 3-D dynamic rupture simulations for the 2011 Tohoku Mw9.0 earthquake through considering the heterogeneous stress condition (with constraints from fault geometry and the stress drop pattern) and the rate- and state friction law (with strong weakening at high slip rate). The rupture model recovers the first-order rupture characteristics, including slip pattern, rupture duration, rupture speed, and moment. The authors claim that the preferred model displays complex depth-dependent radiation (pulse or crack) and rupture reactivations as observed in the 2011 Tohoku earthquake. The topic of this study is intriguing, and the manuscript is well written. However, I have two major concerns regarding the reliability of the rupture model and the depth-dependent radiation, respectively. More validations are needed to support current conclusions. Therefore, I would recommend a major revision in this round. My comments are as below.

We thank Reviewer 3 for the comprehensive review and the constructive comments and suggestions.

Major concerns:

R3.2
1. The 2011 Tohoku Mw9.0 earthquake was well-recorded by the observational networks in Japan and worldwide, and many static and kinematic models have been obtained based on those data. Currently, the dynamic model in this study is only validated with static displacement data. Since this study focuses on the dynamic rupture processes of the 2011 Tohoku Mw9.0 earthquake, I would suggest validating the model with more seismic waveforms, such as local strong-motion data, and showing to which degree this model can capture the rupture processes.

Thank you for the suggestion. We now include a comparison with regional strong-motion records (new Supplementary Fig. S12, and L279–281). Our models are not inversions. We present an ensemble of forward models, designed to analyze the physical mechanisms controlling rupture complexity. Thus, waveform matching is used for verification and not as an optimization target.

Supplementary Fig. S12 compares observed ground motions with synthetics from the preferred model with multiple reactivation and from an alternative, simpler pulse-crack model (see Fig. 8c). Synthetic seismograms from the preferred model show multiple move-out branches that are consistent with the observed waveforms, whereas the simple rupture model produces a single dominant phase. In particular, at stations MYG014 and MYG017 near the main slip patch, the preferred model reproduces the observed waveform complexity more faithfully than the simple pulse-crack scenario.

R3.3

Particularly, the authors suggest multiple rupture fronts rather than a smooth rupture front expanding (e.g., Figs. 6a and 6b). Given very different rupture processes, the two models should produce different ground motions at local strong motion stations. Then a comparison between the two models and the real data will help to validate the multiple rupture fronts.

Thank you for the suggestion! We have added the suggested comparison in the new Supplementary Figure S8, see also our previous response to **R3.2**.

R3.4
Another related comment is that the variance reductions in the static displacement fitting are 77% and 55% onshore and offshore, respectively, which are not very high. Could the authors comment on the fitting performance? How reliable is the final slip distribution in this model?

We thank the reviewer for raising this point. The preferred forward model does not maximize variance reduction by construction. Our approach is not an inversion or a kinematic source model, but a set of spontaneous dynamic rupture experiments designed to investigate mechanisms that generate rupture complexity, as now better clarified in our manuscript (see our response **R3.2** above).

Also above, in response to **R1.4**, we explain that we additionally ran simulations based on three different finite-fault slip models (Melgar et al., 2016; Yamazaki et al., 2018; and Kubota et al., 2022) to sample the range of observationally inferred heterogeneity. Despite these distinct input heterogeneity patterns, all dynamic models produce comparable rupture complexity, including multiple reactivations and large shallow slip. Thus, the preferred slip distribution should be viewed as one dynamically self-consistent realization that fits the first-order static and tsunami constraints, rather than a unique high-precision reconstruction of the actual slip, and the key rupture features discussed in the paper are robust to variations in the underlying slip heterogeneity.

R3.5
2. The second major concern is regarding the high-frequency and low-frequency radiation in the model. Here the authors claim that the model reproduces the depth-dependent radiation pattern, as shown in figure 4. However, the shallow parts feature high peak slip rates (~ 5 m/s in Fig. 2c) and sharp slip rate rises (Fig. 4a), which can contribute to the high-frequency radiation. I feel like the current conclusion is not fully convincing to me. Here are my suggestions on conducting the radiation analysis in a more rigorous way.

We thank the reviewer for the detailed comments and constructive suggestions on the seismic radiation analysis. We agree that shallow, high peak slip rates and sharp slip-rate onsets can contribute to high-frequency radiation. Our revised analysis now quantifies the depth dependence of radiated energy and frequency content more rigorously and separates the contributions from shallow and deep fault segments (see responses **R3.6** and **R3.7**, below).

Most back-projection studies point to dominant downdip high-frequency radiation, while Fang et al. (2018) infer limited updip radiation during the first half of the rupture. This is consistent with our preferred model, in which an updip-leading rupture front with short rise time produces modest early high-frequency radiation, followed by stronger downdip radiation from deeper pulse-like slip. We have updated the manuscript to clarify this interpretation and to document the revised radiation analysis.

Reference:
Fang, H., Yao, H., & Zhang, H. (2018). Earthquake rupture imaging with the wavelet domain compressive sensing: methodology and application to the 2011 Tohoku earthquake. *Geophysical Journal International*, 215(3), 2060-2070.

R3.6
i. The panel 4a shows the slip rate evolution at some points on the fault at different depths. However, the locations of those sampling points are unknown. In addition, the slip rate at depths shallower than 10 km is missing, which should host significant fault slip and radiation.

Thank you for this careful check. The slip-rate time series in Fig. 4a are sampled at receivers along the same along-dip profile through the hypocenter shown in Fig. 3. In the revised figure, we also include three additional slip-rate histories from depths shallower than 10 km and their corresponding spectra in Fig. 4b. Along this profile, receivers are spaced at 10 km intervals, so that the sampled slip-rate functions and spectra systematically cover the shallow to deep portions of the megathrust.

R3.7
ii. In figure 4b, the spectra are normalized. Given the large slip at shallow depth, the amplitudes of the low-frequency spectral plateau should be much higher at shallow depths. Therefore, the normalization may cause apparently weak high-frequency radiation for shallow depths and bias the comparison of high-frequency radiation intensity at different depths. I would suggest the authors compare them without normalization (maybe taking the spectral ratio can better illustrate it).

We appreciate this comment. In the revised Figure 4b, we now show absolute power spectral densities without normalization, allowing a direct comparison of spectral amplitudes with depth. The updated spectra confirm that shallow segments have higher low-frequency plateaus, consistent with their larger slip, whereas the deeper pulse-like rupture exhibits relatively stronger high-frequency content. Figure 4 has been updated accordingly, with additional shallow slip-rate functions in panel (a) and the unnormalized spectral density comparison in panel (b).

R3.8
iii. In panels c-e, the authors choose the period of 10s to filter the slip rates. To my best knowledge, high-frequency BP analyses are usually conducted in the frequency band of 0.5-2 Hz, such as in Lay et al. (2012 JGR) and Meng et al. (2011 GRL) for the Tohoku event. To make

a fair comparison, I would suggest using a similar bandpass filter to show the pattern or showing it in multiple frequency bands.

We thank the reviewer for pointing out the mismatch in the frequency range. To enable a direct comparison with previous back-projection studies (e.g., Lay et al., 2012; Meng et al., 2011), we now compute and show band-pass–filtered slip-rate and radiation patterns in three period bands: 10-2 s (0.1-0.5 Hz), 2-0.5 s (0.5-2 Hz), and a high-pass filter above 0.5 s (>2 Hz). The new Supplementary Figure S6 demonstrates that downdip pulse-like rupture dominates the 0.5–2 Hz band used in most back-projection analyses, whereas the shallow updip rupture contributes mainly at longer periods, consistent with our interpretation of depth-dependent radiation.

R3.9
Minor comments:
1. Figure 1a: the 3-D view of the fault plane is a little bit hard to read. Could the authors refine the design of this panel?

We have increased the contrast between the fault plane and the surrounding domain. We have also updated the cross-section for better separation of the fault plane.

R3.10
2. Figure 5: it is hard to tell the location of the transition from rate-weakening to rate-strengthening. Changing the colorbar may help.

We have updated the colormap to increase the contrast between rate-weakening and rate-strengthening.

. In response to their remaining comments, wehave further clarified the scope and limitations o f our modeling approach, added a few references, extended the discussion of limitations, and strengthened the analysis of depth-dependent rupture behavior through two new supplementary figures and minor text revisions.

Please find below our point-by-point responses to the reviewers' comments. Reviewer's comments are in black, our responses are in blue, the main text is in red, and **the corresponding edited text is in bold red**. The line numbers refer to the "clean" version of the revised manuscript.

Thank you for considering our manuscript for publication in Nature Communications.

Jeremy Wing Ching Wong, Alice-Agnes Gabriel, Wenyuan Fan

REVIEWER COMMENTS

Reviewer #1 (Remarks to the Author):

R1.1 The authors have addressed the points that I made in my original review quite thoroughly. The manuscript is rich in information and the simulations are very impressive. My main concern about dynamical modeling is that it is so complex (many highly uncertain parameters and physical processes as well as uncertain prestress) that fitting of all types of data is not yet really viable, and the preferred model here remains very limited in even predicting the static geodetic offsets and gives only a first-order fit to the moment rate function, but that is the state of the art. I think the inclusion of additional heterogeneous prestress models in the revision is helpful; there is quite a strong sensitivity of the resulting models, even though the qualitatively complex ruptures do have some comparable features. This study is a very informative examination of some of the complex representations of prestress and frictional behavior that are intrinsic to dynamic modeling, but it remains unclear to me how to progress to models which actually match observations at the level of simple (possibly physically unrealistic) kinematic inversions. Clearly much is yet to be done. But this is an excellent study to guide the community. I recommend publication as is.

Thorne Lay

We thank Dr. Lay for his careful assessment and for emphasizing both the strengths and current limits of physics-based dynamic rupture modeling. We note that our manuscript includes a "Limitations" section in the Discussion detailing many of these aspects.

We have now explicitly clarified (in L400-404) that the goal of this study is not to perform an inversion nor to achieve a perfect fit to all observations, but to identify dynamically viable rupture scenarios that capture the key source characteristics and underlying physical mechanisms. We also highlight in L394-395 that extending fault slip modeling beyond the dynamic rupture (coseismic) timescale in future work may help to better constrain dynamic parameters.

Discussion - limitations (L400-404)

The goal of this study is to identify dynamically viable rupture scenarios that reproduce the key source characteristics and illuminate the governing physical mechanisms, rather than to perform an inversion of all available observations. However, characterizing dynamic parameters, including shear stress and frictional properties, remains challenging because of strong trade-offs (e.g., Schmedes et al., 2010). Whereas this work focuses on coseismic dynamic rupture, future work may integrate observations across time-scales to help constrain the governing faulting conditions (Premus et al., 2022; Schliwa et al., 2024).

References:
Schmedes, J., Archuleta, R.J., Lavallee, D., 2010. Correlation of earthquake source parameters inferred from dynamic rupture simulations. J. Geophys. Res. 115,

B03304. doi:10.1029/2009JB006689.

Premus, J., Gallovič, F., & Ampuero, J. P. (2022). Bridging time scales of faulting: From coseismic to postseismic slip of the M w 6.0 2014 South Napa, California earthquake. Science advances, 8(38), eabq2536.

Schliwa, N., Gabriel, A. A., Premus, J., & Gallovič, F. (2024). The linked complexity of coseismic and postseismic faulting revealed by seismo-geodetic dynamic inversion of the 2004 Parkfield earthquake. Journal of Geophysical Research: Solid Earth, 129(12), e2024JB029410.

Reviewer #2 (Remarks to the Author):

R2.1 The scientific merit of this work that I expected to be there after the first review still stands. It is a significant contribution to our understanding of megathrust earthquakes and the mechanisms behind it. I therefore think it worthy of publication in Nature Communications.
The main difference between this version and the previous version is in how well the work is explained. The authors also did incorporate some new sections and additional models. The main issue with the previous version was the lack of clarity on what exactly was done and how to interpret the results. So this mostly structural change and improved explanation is exactly what the manuscript needed. The paper is now a standalone paper that can be understood (mostly) without reading any supplementary information, which is exactly how it should be. Because some parts of the manuscript became a lot clearer to me, I do have some additional comments for further improvements, but these are mostly minor.

Response to previous comments:

- Most of my previous comments are resolved. The manuscript benefits from the additional length, which does justice to the complexity of the model.There is a big improvement in clarity, much easier to understand now. I appreciate the restructuring of the introduction, the added sections to the discussion and the additions of Table 1 and the list of supplementary figures.

We thank Reviewer 2 for the detailed comments on the manuscript's clarity and structure.

R2.2 - For one thing, it is now clear to me that the authors talk about dynamic (co-seismic) frictional weakening, not changes in rate-state friction parameters. It would help if authors can consistently call it dynamic weakening, which is the common term for this effect. It is (especially in the introduction and end of the paper) sometimes called " fast-velocity weakening" or "rapid velocity-weakening rate and state friction" (e.g. lines 48, 62, 76, 90, 381, also in methods line 470). This is confusing, especially as the fault is actually v-strengthening (positive a-b) on part of the fault surface. It would help if the authors can consistently call this dynamic (or co-seismic) weakening. The same with the coseismic restrengthening, which is the correct term, but sometimes it is called healing (e.g. subtitle line 148). Healing is a time-dependent process that is not incorporated into the model and not what the authors mean. It is clear to me now that the

authors mean the rapid recovery in friction coefficient as the fault velocity drops, but calling it healing is confusing.

Thank you for the comment. We fully agree that our mixed terminology could cause confusion between rate-and-state evolution and fast-velocity-weakening behavior. We revised the manuscript to use “dynamic weakening” for the rapid coseismic reduction in frictional strength at high slip rates, and “coseismic frictional restrengthening” for the rapid recovery of friction coefficient as slip rate decreases. We avoid the term “healing” where it could be interpreted as time-dependent interseismic strengthening. However, when describing pulse-like rupture, we clarify that “self-healing” refers to the established term of self-healing front of a slip pulse (Perrin et al., 1995; Nielsen & Madariaga, 2003) and is distinct from long-term frictional healing (L145–147). Throughout the manuscript, we have now also unified the wording to “fast velocity-weakening rate-and-state friction”. We have updated the subsection title from “*Dynamic rupture reactivation driven by rapid coseismic **frictional healing***” to “*Dynamic rupture reactivation driven by rapid coseismic **frictional restrengthening***”.

Results: Dynamic rupture reactivation driven by rapid coseismic frictional restrengthening
L145 - 148
Crack-like rupture involves prolonged local slip duration (rise time) comparable to the total event duration. In contrast, self-healing pulse-like rupture is characterized by short rise times **and a “healing” front following the rupture front driven by rapid coseismic fault strength recovery** (Heaton, 1990**; Perrin et al., 1995;** Nielsen & Madariaga 2003).

Newly added References:
Perrin, G., Rice, J. R., & Zheng, G. (1995). Self-healing slip pulse on a frictional surface. Journal of the Mechanics and Physics of Solids, 43(9), 1461-1495.

R2.3 - One of my other major points was a lack of description of alpha and R0. This has much improved as well. I think I understand alpha now, it is a amplification factor of spatial heterogeneities from the mean slip model, where the location of heterogeneities comes directly from the model. So the location of asperities is not varied in this part, although with the new alternative models, there is variation in locations of the stress heterogeneities. This could be clearer in the main text, but is understandable to me from the methods now. I am still a little unsure where the actual numbers for R0 come from and how much the pre-stress levels actually vary in MPa's.

We appreciate the comment. We added the range of initial shear stress levels corresponding to the R_0 range in L629.

Methods - Ambient prestress L629:
We systematically vary R_0 from 0.1 to 0.2 to identify the preferred rupture model. This range corresponds to an initial shear stress of 3.12–4.05 MPa at hypocentral depth.

R2.4 - Line 225-226 “expected behavior of clay-rich sediments: I’m glad the authors could correct me on my previous point 2.20 that sediments also have dynamic weakening. I learned something new today 😊. The text is now too specific though, because it only talks about clay-rich sediments. But most of your model is at depths (and stresses) where rocks are lithified. The value for f_w you use also better fits with the large amount of work that exists for lithified rock samples. On a side note, I do wonder if the large amount of fluids in the shallower parts might increase the amount of thermal pressurization hence dynamic weakening.

We appreciate this suggestion. We revised the relevant sentence to make a more general statement about shallow fault materials and to avoid implying that the entire shallow segment behaves as unlithified sediment. The revised text now references both borehole-derived fault gouge measurements and laboratory constraints on lithified rocks and rock gouges (L226-231). We also expanded the Discussion to acknowledge additional processes that may contribute to rupture complexity beyond our simplified frictional and structural assumptions (L388).

Results - Large slip to trench: L226-231
The velocity-strengthening friction (<9 km depth) and fast velocity-weakening friction adopted in our model **are motivated by** laboratory friction measurements on borehole-recovered fault gouge samples from the shallow high-slip region of the Tohoku-Oki rupture (Ujiie et al., 2013) and **by laboratory constraints indicating that**
clay-rich lithified rock and rock gouges commonly exhibit velocity-strengthening to transitional behavior at low effective normal stress (Saffer et al., 2003). **This assumption is intended as a generalized representation of shallow fault materials and does not imply that the entire shallow megathrust segment behaves as unlithified sediment.**

Discussion - Limitation L388:
Whereas these simplifications enable systematic exploration of how frictional and prestress parameters control dynamic rupture, additional processes proposed to influence rupture complexity in large megathrust earthquakes are not represented here, including along-strike variations in shallow material properties and sediment heterogeneity (Moore et al., 2015), accretionary wedge geometry (Tsuru et al., 2002), interface roughness and upper-plate structural heterogeneity (Bassett et al., 2015), bimaterial effects across the plate interface (Shi et al., 2006; Scholz, 2014; Kozdon & Dunham 2013), **or spatial variations in pore-fluid pressure (Noda & Lapusta, 2013).** Incorporating these 3D effects is an important direction for future work.

References:
Noda, H., & Lapusta, N. (2013). Stable creeping fault segments can become destructive as a result of dynamic weakening. *Nature*, *493*(7433), 518-521.

Other comments:
R2.5 From figure 1b it is clear how the 'normal' RSF formulation is varied, but this is not so well explained in the main text

We expanded the main text model description to define the prescribed depth profile of the rate-and-state parameter (a−b) shown in Figure 1b and to clarify how it transitions from velocity strengthening in the shallow wedge to velocity weakening in the seismogenic zone and back to velocity strengthening downdip (see L101–105).

Results (Introducing model setup): L101-105
We prescribe a depth-dependent profile of the rate-and-state friction parameters a-b (Figure 1b). The shallow megathrust is assigned velocity-strengthening friction (a−b>0), consistent with laboratory constraints for **clay-rich lithified rock and rock gouges** at low slip velocities (Saffer et al., 2003). **The seismogenic zone (10-45 km depth) is assigned velocity-weakening friction (a−b<0). At the downdip limit of the seismogenic zone (> 45 km), we revert to velocity-strengthening friction, consistent with inferences from repeating earthquakes and slow-slip events (Uchida et al., 2021). For more details, see the Methods section "Fault Friction".**

R2.6 It is also not well explained in the main text if the full RS formulation is used, with state evolution etc. This does become clear from the methods, but a short sentence with reference to the methods would be nice.

Thank you for this comment. Typically, dynamic rupture simulations include the full RS formulation. We have clarified in the main text that our simulations use the full rate-and-state friction formulation (L88), including state evolution, and we added an explicit pointer to the Methods (Eqs. 5-6). We also clarified how the friction law behaves across slip-rate regimes. At low slip rates, it follows standard rate-and-state friction, and as the slip rate approaches/exceeds Vw, it transitions to fast velocity-weakening behavior (L95-97).

Results (Introducing friction): L88 - 97:
We use the full rate-and-state friction formulation, including state evolution (Methods Sec. "Fault friction", Equations. 5-6). It depends on slip rate V and transitions from a low-velocity steady-state friction coefficient $f_{LV,\,ss}(V)$ to a fully weakened level f_w once V exceeds the onset of weakening velocity V_w.
…
where f_0 is the reference friction coefficient, V_0 is the reference velocity, a is the direct-effect parameter, and b is the state-evolution parameter.
At low slip-rates ($V<<V_w$), friction follows the classical rate-and-state friction law, whereas for high slip rates ($V>=V_w$), an additional fast velocity-weakening term is activated, producing a rapid reduction in frictional strength.

R2.7 Why is slip accelerating near the trench? This should be unexpected as a-b becomes positive there? There is a section about large slip amount, off-fault plasticity and negative stress drop. But especially the acceleration of slip puzzles me. Can the authors comment on this too (would fit quite well in that section).

Thank you for the questions. We clarified this point in L225-228. Although our model incorporates velocity strengthening (a-b)>0 in the shallow segment, that property governs frictional evolution at low slip rates and during stable, steady-state sliding. This does not preclude transient slip acceleration during dynamic rupture.

In our model, strong wave-mediated dynamic stressing, from both free-surface-reflected rupture fronts and from updip-propagating rupture fronts reactivated downdip, increases shear traction on the shallow interface and drives slip rates toward Vw. Reaching Vw activates additional fast-velocity weakening, which produces a second acceleration phase (Fig.3b, Supplementary Figure S5a).

Results - Large slip to the trench L225-228
In our model, dynamic frictional weakening effectively sustains shallow rupture propagation, enabling up to 50m of slip near the trench. **Wave-mediated dynamic stressing, including both free-surface-reflected rupture fronts and updip-propagating fronts reactivated downdip, increases shear traction and slip rate on the shallow interface. As shallow slip rates approach and eventually exceed V_w, fast-velocity weakening friction is activated, producing a second acceleration phase (Fig. 3b, Supplementary Figure S5a).**

R2.8 Line 167: “Rapid coseismic restrengthening emerges as the principal mechanism controlling repeated reactivation: There is rapid coseismic restrengthening both updip and downdip, so this does not explain why there is only pulse-like behaviour downdip.

Thanks for the question. We better clarify this in L176-179. The sustained updip rupture limits the formation of a healing front and coseismic restrengthening, thereby leading to the depth-dependent rupture behavior. Thus, a key difference is not whether coseismic restrengthening occurs, but whether it is sufficient to arrest slip. Updip, sustained crack-like slip and continued dynamic loading keep shear traction above fault strength, so healing fronts are not sustained, and pulse-like rupture does not develop. Downdip, restrengthening more readily arrests slip, allowing self-healing pulses and repeated reactivation.

Results - Dynamic rupture reactivation driven by rapid coseismic frictional restrengthening L176-179:
Notably, all secondary ruptures are pulse-like in the downdip direction and coalesce with the free-surface reflected front into crack-like ruptures in the updip region.
The sustained crack-like slip updip and continued dynamic stressing limit the formation of healing fronts, and pulse-like rupture does not develop there. Thus, a key difference is not whether coseismic restrengthening occurs, but whether it is sufficient to arrest

dynamic slip. Downdip, restrengthening more readily arrests slip, allowing self-healing pulses and repeated reactivation.

R2.9 Line 287 “self-healing”: how is self-healing visible in the models? Is this the co-seismic increase of friction as rupture speed drops? Is it really healing?

As noted above in response to R2.2, the “self-healing” refers to the well-established term of the self-healing front of a slip pulse (Perrin et al., 1996, Nielsen & Madariaga, 2003). We have further clarified this in L298.

Discussion - Impact of prestress on dynamic rupture complexity L298:
In contrast, heterogeneous stress conditions in our models cause locally low prestress regions, particularly near the hypocenter and downdip, which facilitate the formation of self-healing pulse-like ruptures (Zheng & Rice, 1998; Perrin et al., 1996).
Subsequent slip arrest at depth reaccumulates shear stress along previously ruptured segments (Nielsen & Madariaga, 2003), thereby enabling reactivation.

R2.10 Line 344 “additional frictional heterogeneity”, does this mean you have the frictional heterogeneity on top of the prestress heterogeneity? This is not clear from the main text.

Good question. In these simulations we impose heterogeneity in the rate-and-state state-evolution distance L in addition to (i.e., superimposed on) the prestress heterogeneity of the preferred model. We updated the text accordingly in L350.

Discussion - Frictional heterogeneity L350:
We superimpose heterogeneity in the state-evolution distance L (0.3-1.0 m) onto the preferred model, which includes prestress heterogeneity. Despite this added frictional heterogeneity in L, the simulated dynamic rupture evolution remains qualitatively consistent with the preferred model.

R2.11 I do believe the methods could be a lot more to the point and with a lot less references. E.g. the depth-dependent effective normal stress, it would suffice to say this is based on a lithostatic gradient with a hydrostatic gradient of 0.9. Might be nice to add where you get the densities from, but all other references are unnecessary. Other section might be shortened similarly. This would make it easier to focus on the specifics that matter, like your model description.

We note that earlier revision rounds requested additional citations to better contextualize our parameter choices and to fully acknowledge previous work. Pore fluid pressure controls the frictional strength and governs rupture dynamics (Madden et al., 2020). Direct observations from Saffer& Tobin (2011) and Jamali Hondori & Park (2022), and inferences from focal mechanisms by Hardeback & Loveless (2018), support elevated pore-pressure conditions in the Japan Trench Subduction zone. We use the same 1D velocity structure (Supplementary Table S2)

used in the simulation to compute the density and depth-dependent effective normal stress, and added the reference in L543.

Methods - Depth-dependent effective normal stress L534 - 545:
Pore fluid pressure plays an important role in controlling the effective normal stress and thus the shear stress conditions and frictional strength governing earthquake rupture dynamics (e.g., Madden et al., 2022). **Drilling observations and seismic reflection studies (e.g., Saffer & Tobin, 2011; Jamali Hondori & Park, 2022) as well as stress orientation analyses (Hardebeck & Loveless, 2018) suggest elevated pore-fluid pressure in the Japan subduction zone, with measurements within the accretionary wedge and along the shallow fault interface reaching 80-95\% of lithostatic stress.**
We assume that pore fluid pressure reaches 90% of lithostatic stress in all layers.

The lithostatic stress is defined as $P_{litho}(z) = \int_0^z \rho_i g h_i dz$, where the subscript i refers to the respective layer in the velocity model **(Supplementary Table S2)** and g = 9.81 m/s^2 is the gravitational acceleration. This assumption results in depth-dependent effective normal stress and relatively low fault strength everywhere (Supplementary Fig. S1c).

Minor:

R2.12- In figure 3b, it seems to be the distance on the x axis is quite arbitrary, and the right edge is the trench? Would be nice if this could be annotated, easier for people who see the figure for the first time.

Thank you for this suggestion. We added the trench annotation in Figure 3b.

R2.13 - Line 165 “slower apparent rupture speed”: it seems to me the actual rupture speed is similar, but it is intermittent rather than continuous. I wonder if “slower apparent rupture speed” the right way of wording this.

Thank you for this comment. We agree and have clarified the wording in L162 to distinguish the apparent rupture velocity from the fault-local rupture speed of each reactivated front.

Results - Dynamic rupture reactivation driven by rapid coseismic frictional restrengthening L162:
Figure 3b shows how episodic pulse-like rupture reactivation successively extends the rupture duration downdip, producing a slower apparent rupture speed (1.5km/s), **even though each reactivated front locally propagates at typical rupture speeds (2.5~km/s), respectively.**

R2.14 - Line 269-271 “We next investigate .. ambient stress level”: this is a bit confusing. It reads like: We measure a and b, which are alpha and R0, which represent b and a (reverse order). So which is which does not become clear from this sentence. Would be much clearer if

in l 270 could be rewritten like: "depends on the amplitude of stress heterogeneity (alpha) and relative prestress level (R0), by systematically varying both"

Thank you for pointing this out. We have updated it accordingly.

R2.15 - Line 276 “Equation 16”, can you add this is in Methods? Because there is still methods and supplementary information.

Thank you for this comment. The ‘S ratio’ equation is already listed in Methods. We now added a reference to the Methods section in L286.

Discussion - Impact of prestress on dynamic rupture complexity L286:
We find that our preferred rupture style requires low fault strength compared to the dynamic stress drop, corresponding to a low seismic S ratio (Das & Aki, 1977) (Equation 16, ratio of initial strength excess over dynamic stress drop, **see Methods Sec.``Prestress**"). The S ratio measures the balance between fault proximity to failure and the available stress release during dynamic weakening. Lower effective normal stress and fault strength or higher initial shear stress favor reactivation-dominated scenarios.

R2.16 - Line 401-403 “Similar rupture complexities … future megathrust earthquakes”. This is not mentioned in the discussion and should not be a conclusion of this work.

We moved this section to the discussion on rupture complexity (L264-266).

Discussion - Comparison of simulated rupture reactivation with observations L264-266:
Strong-ground motion move-out patterns and finite-fault slip models of the Tohoku-Oki earthquake provide evidence for multiple rupture episodes (Ide & Beroza, 2011; Lee et al., 2011; Yue & Lay 2011; Melgar & Bock, 2015).
Similar rupture complexities have been documented in other large megathrust earthquakes, including the 2010 Mw 8.8 Chile earthquake (Okuwaki et al., 2014) and the 2019 Mw 8.0 Northern Peru earthquake (Vallee et al., 2023).

R2.17 - SM1, SM2, and SM3 are not referred to at all. Are those necessary? I do think they could be incorporated in the methods in a shorter form.

Supplementary Materials on mesh quality, nucleation, and dynamic stress drop provide key technical details for our dynamic rupture simulations. We have now added explicit pointers to these sections in the main text. In the Results section, **L77** on mesh resolution now references **SM1: Mesh quality**. The discussion of the moment-rate release for the preferred model in **L133** now references **SM2: Nucleation**. The dynamic stress drop of the preferred model in **L138** now references **SM3: Dynamic stress drop**.

R2.18 - Supplementary figure S19 is a bit confusing, because the dynamic stress drop is velocity-dependent, not slip-dependent. By plotting this with slip on the x-axis, it almost looks like a slip-weakening mechanism, which is not what you use.

Thank you for the comment. We agree that it is interesting to also show the frictional evolution against the slip rate. We therefore revised Supplementary Figure S20 to add a new panel showing the same schematic frictional evolution as a function of slip rate V. We accordingly revised the caption to clarify that the slip-based panel is shown only as an illustrative trajectory along a representative fault-point history and should not be interpreted as a slip-weakening mechanism.

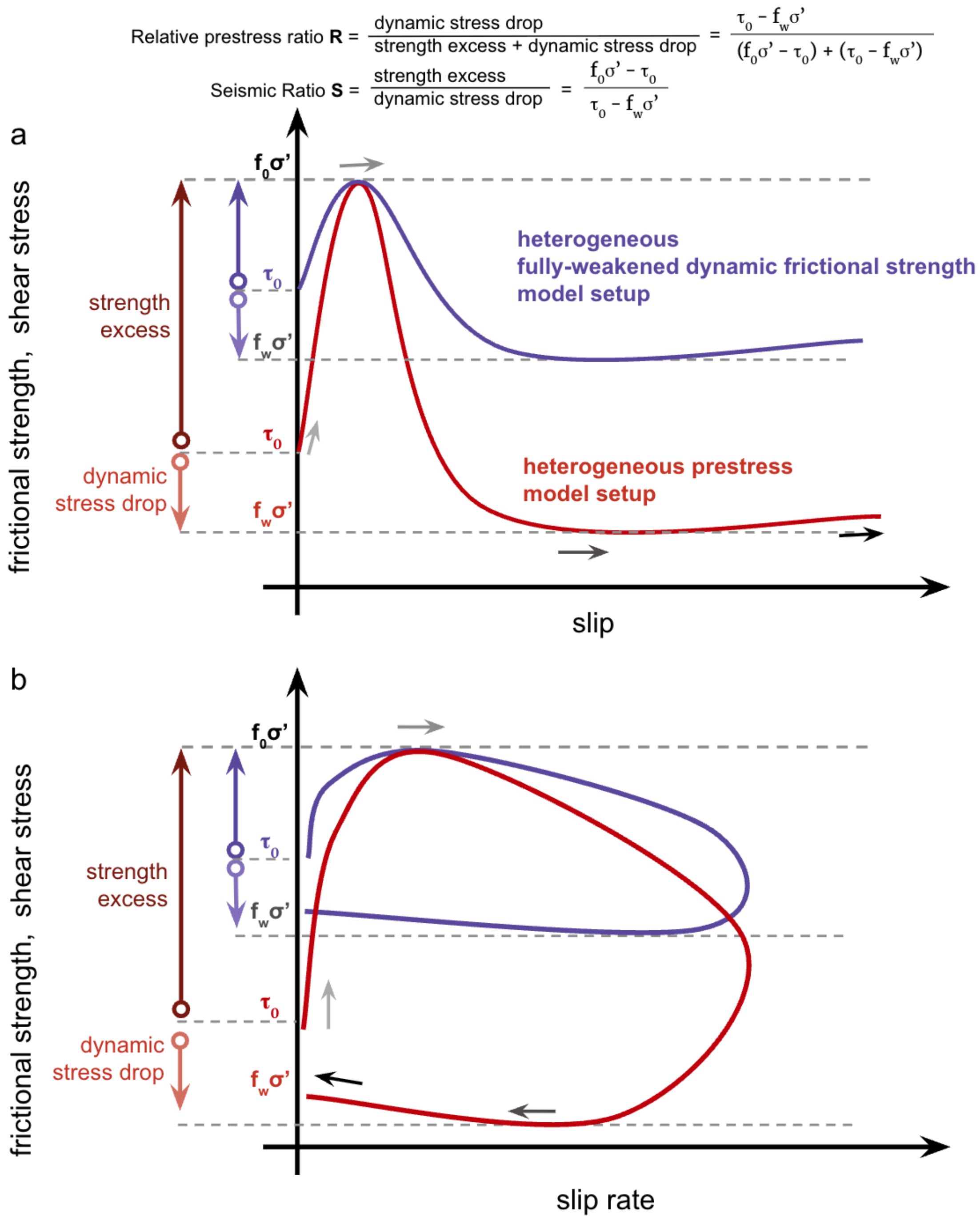

Updated Supplementary Figure S20. **Modeled fault-local frictional evolution in two alternative model setups with heterogeneity either in friction or in prestress. a) friction evolution with slip. b) frictional evolution with slip rate.** The purple curve shows the frictional strength evolution for the setup with a heterogeneous fully-weakened dynamic frictional strength. The red curve shows the frictional strength evolution for the preferred model with heterogeneous prestress. The grey arrows in both panels show the stages of frictional evolution correspondingly. The darker arrows in the y-axis labels indicate the frictional strength excess, and the lighter arrows indicate the dynamic stress drop. We added the definitions of the seismic

ratio S and relative prestress level R. Given the same dynamic stress drop value, the heterogeneous fully-weakened dynamic frictional strength model has a lower ratio of strength excess to dynamic stress drop (S ratio, equation 16) than the heterogeneous prestress model.

Reviewer #3 (Remarks to the Author):

R3.1 I appreciate the authors' efforts in addressing my comments. In particular, I appreciate the new Figure 9, which is informative. The physical phenomenon raised in this study is interesting.

We thank Reviewer 3 for the positive feedback!

R3.2 As the authors set the model particularly following the properties of the 2011 event, especially the initial stress condition, I suppose one of the targets of this study is to provide a reasonable rupture model for this event. However, the updated results of the frequency analysis and the comparison with the data are not yet sufficient to fully support the argument that the presented models really capture the physics of the 2011 Tohoku earthquake. I would suggest being more conservative/cautious about statements related to comparisons with the 2011 event and clarifying related limitations clearly. Here are my detailed comments.

Thank you for the detailed comments. Following the comments in R3.3 and R3.4, we added two new supplementary figures (S7 and S10) to improve the frequency analysis of slip rate on the fault across different profiles and off-fault strong-ground-motion synthetics. Both comparisons support our modelled rupture behavior. In this revision, we further clarified the goal of our studies (see R1.1) and conditions governing the depth-dependent rupture behavior (see R2.8). We also acknowledged our limitations and highlighted other proposed mechanisms (R3.3). Please see our detailed response below.

R3.3 1.I think the most important argument of this study is "the multiple rupture fronts emerging downdip are responsible for some intriguing observations in the 2011 Tohoku earthquake." I agree on the point that the presented model can explain the re-rupture process at the hypocenter region, which has been reported in previous literature. However, whether this mechanism can explain the depth-dependent radiation remains unclear to me. As shown in new Figures 4c and S6, the peak slip rate is comparable at shallow and deep portions at high frequency with just slight dependence on depth, which appears inconsistent with the reasonable trend of enhanced high-frequency energy shown in the spectra in Figure 4b. One potential explanation is that Figure 4b only captures the on-fault stations along the hypocentral dip profile. If the authors set the stations at other locations, such as with the latitude of ~39°, will the trend in Figure 4b still exist? I think this is very important to argue not only based on one profile but for the whole rupture. The authors argue with a paper (Fang et al., 2018). However, I think the depth-dependent trend is the same in most papers. So, it is important to argue for the trend over the whole rupture.

Thank you for raising this point. To test robustness, we added a new supplementary figure (Supplementary Fig. S7) that compares multiple dip profiles spanning the rupture from north to south, including profiles outside the hypocentral region (e.g., around ~39°N). Across these profiles, the modeled rupture systematically transitions from short-duration, pulse-like slip at depth to longer-duration, crack-like slip updip, with local rise time decreasing with depth and fault activation duration increasing toward the trench.

This systematic change in the local source time function shifts spectral energy toward higher frequencies at depth, consistent with the trend shown in Fig. 4b. We acknowledge in L194-201 that, despite the rise-time contrast, the initial rupture-front passage can produce a pronounced slip-rate peak at many depths, so shallow portions can still contribute appreciable high-frequency energy in our simulations. We now state this explicitly, beyond the imposed depth dependence in (a−b) and effective normal stress, we do not include additional shallow fault-zone processes that would further suppress rupture-front slip-rate peaks and high-frequency radiation near the trench. We therefore interpret rupture reactivation and the emergence of multiple rupture fronts as one contributor to the observed depth-dependent radiation, but not a complete explanation on its own (L387-391).

Results - Downdip slip pulses and updip crack-like rupture L194-201:
These depth-dependent rupture styles become particularly evident during 30-80s rupture time, when multiple reactivated rupture fronts are present, and the deeper ruptures manifest discrete short-duration pulses (Fig. 4a, Supplementary Fig. S5). **Additional profiles from north to south show consistent behavior, with rupture durations systematically increasing towards shallower depth (Supplementary Fig. S7).** This rupture style variability influences the associated seismic radiation. Figure 4b shows slip-rate amplitude spectra, revealing that shallow crack-like rupture episodes are relatively depleted in high-frequency energy than the deeper pulse-like rupture portions. **However, northern profiles exhibit less contrast in seismic radiation due to a strong peak at the rupture front across all depths (Supplementary Fig. S7). This behavior reflects an intrinsic limitation of our model setup, which does not prescribe any depth-dependent frictional variations that control rupture tip behavior (Huang et al., 2014).**

Discussion - Comparison with previous modelling studies L387-391:
While our simulations reproduce pronounced downdip complexity and a systematic reduction of rise time at depth, back-projection studies of the 2011 Tohoku-Oki earthquake show comparatively weak high-frequency radiation from the shallow rupture (Meng et al., 2011; Lay et al., 2012). Reconciling the discrepancy with our models may require additional mechanisms that may damp shallow peak slip-rates and high-frequency radiation, such as inelastic yielding of the accretionary wedge (Ma, 2023; Ulrich et al., 2022) and depth-dependent frictional variations (Huang et al., 2014).

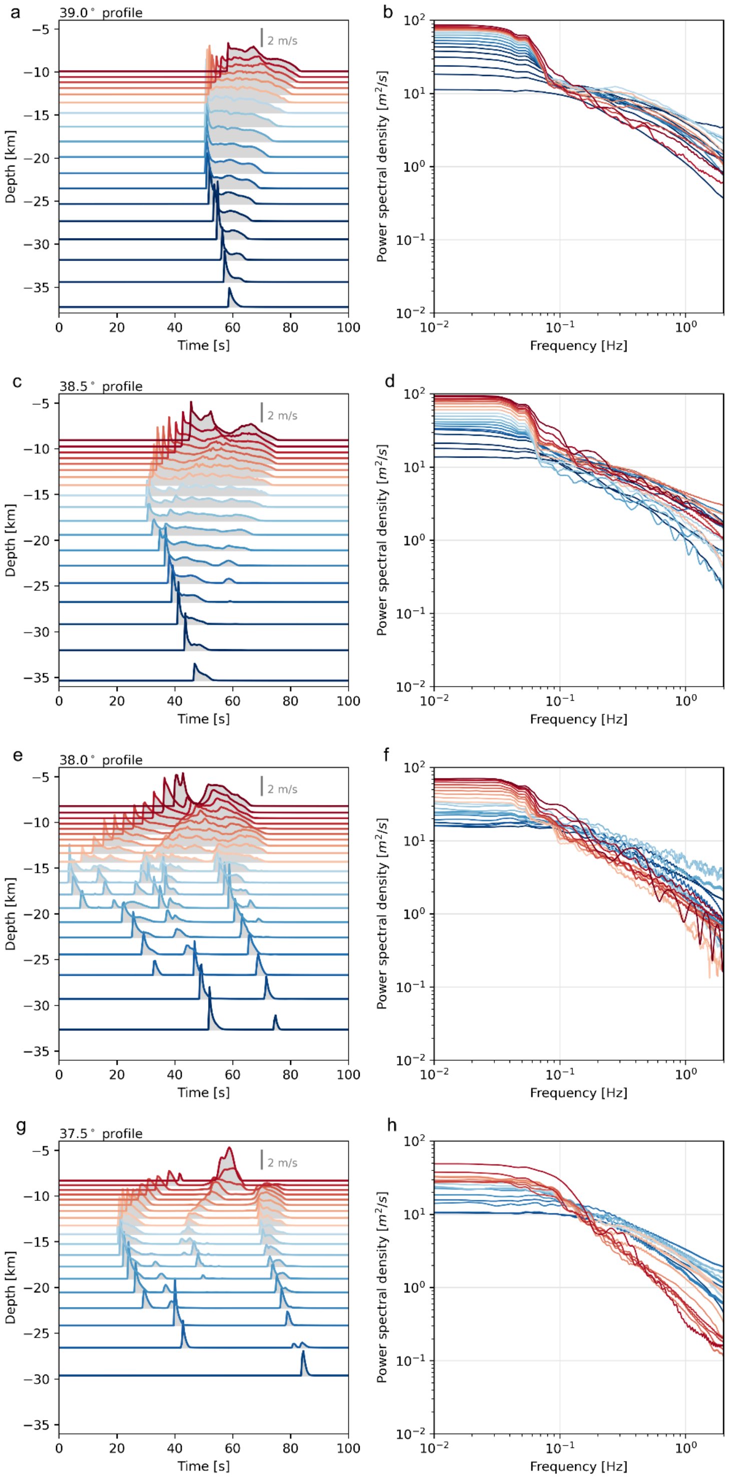

New Supplementary Figure S7: Along-dip slip-rate time histories and corresponding power spectral densities for the preferred model. Left column (a,c,e,g): slip-rate evolution with depth along four dip profiles. Right column (b,d,f,h): power spectral density of the slip-rate time series for each profile. Across all profiles, local slip duration (rise time) increases toward shallower depths; the contrast is most pronounced along the 38.0° profile.

R3.4 2. In this revision, the authors provide the comparison between the model synthetics and the strong motion data in Figure S8. I agree that the multiple-front rupture produces better fitting on the MYG006 and MYG014. But it appears to me that the fitting is overall not sufficient, as it cannot catch the long-period trends or amplitudes. Therefore, I have concerns about the argument in lines 253-254: "In addition, our model generates ground motions that can qualitatively fit the strong-ground motion records near the main rupture area (Supplementary Fig. S8)." I understand that for dynamic rupture models at such a large scale, it is very challenging to fit the data. However, such a level of fit may raise concerns for a 3-D model for a particular earthquake (with pre-constrained initial stress condition), which leads to questions about whether this model can really capture the rupture process to some degree.

Thank you for this comment. We have revised the statement in the main text to be more precise, and we expanded the supplementary material to evaluate the comparison in the frequency domain. Specifically, we added a new Supplementary Figure S10 comparing power spectra between observations and synthetics. This analysis shows that our simulations reproduce the observed spectral levels and overall spectral shape up to ~0.5 Hz at the examined K-NET stations, while discrepancies remain at longer periods and in absolute amplitude, consistent with the simplified structural assumptions (1D velocity model, smoothed geometry, and the fact that we do not perform an inversion).

We have changed the text in Lines 275-277 to "In addition, the preferred model reproduces key qualitative features of strong-motion records near the main rupture area, **particularly the relative timing and spectral content up to 0.5 Hz, although long-period trends and absolute amplitudes are not fully captured (Supplementary Figs. S9–S10).**"

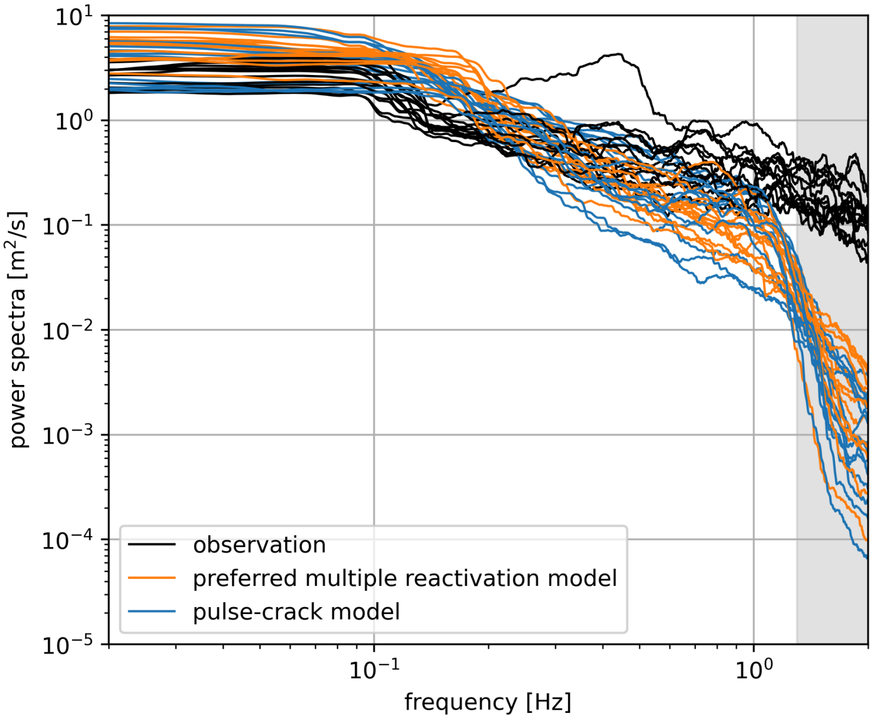

New supplementary Figure S10: Comparison of power spectra of modeled waveforms at onshore strong-motion K-net stations with observations (black), preferred model (orange), pulse-crack model (blue). The shaded area shows the effective maximum frequency resolved in the simulated seismic wavefield at 1.5 Hz.

R3.5 3. Line 120 should be Figure S12.
Thank you for the comment, we corrected the Figure reference.
